# Machine Learning‐Driven Grayscale Digital Light Processing for Mechanically Robust 3D‐Printed Gradient Materials

**DOI:** 10.1002/adma.202504075

**Published:** 2025-07-16

**Authors:** Jisoo Nam, Boxin Chen, Miso Kim

**Affiliations:** ^1^ Department of Mechanical Engineering Korea Advanced Institute of Science and Technology (KAIST) Daejeon 34141 Republic of Korea; ^2^ School of Advanced Materials Science and Engineering Sungkyunkwan University (SKKU) Suwon 16419 Republic of Korea

**Keywords:** 3D printing, dynamic bond, gradient structure, grayscale digital light processing, machine learning, multi‐objective optimization, polyurethane acrylate

## Abstract

Grayscale digital light processing (g‐DLP) is gaining recognition for its capability to create material property gradients within a single resin system, enabling programmable mechanical responses, enhanced shape accuracy, and improved toughness. However, research on the mechanical robustness of g‐DLP is constrained by the limited range of tailorable properties in photocurable resins and insufficient exploration of structural optimization for complex geometries. This study presents a synergistic g‐DLP strategy that integrates the synthesis of dynamic bond‐controlled polyurethane acrylate (PUA) with a machine learning‐based multi‐objective optimization, enabling mechanically robust 3D‐printed gradient materials. A PUA‐based resin system is developed that expands the achievable elastic modulus from 8.3 MPa to 1.2 GPa, while maintaining superior damping performance, making it suitable for diverse applications. Furthermore, a multi‐objective Bayesian optimization framework is constructed to efficiently identify optimal gradient structures, reducing strain concentrations and controlling effective stiffness. This approach is applicable to various 3D and arbitrary geometries, achieving a significant strain concentration reduction of up to 83% and demonstrating delayed crack initiation. By combining the developed material with this optimization framework, a versatile platform is established for creating mechanically robust g‐DLP printed components, applicable in areas ranging from biomimetic artificial cartilage to automotive energy‐absorbing structures.

## Graphical abstract

A machine learning‐driven grayscale digital light processing (g‐DLP) platform integrates optimization of gradient structures and photocurable resin materials with extensive tunability. The optimized gradient structure, fabricated from developed polyurethane acrylates with enhanced damping and customizable stiffness, reduces stress concentration, confirming the mechanical robustness of the g‐DLP platform. This technology offers versatile solutions for applications, ranging from biomedical to automotive.

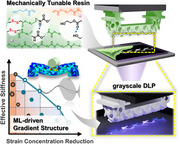

## 1 Introduction

Digital light processing (DLP) is a photopolymerization‐based 3D printing method recognized for its versatile material selection and rapid, high‐resolution capabilities.^[^ ^1^ ^]^ In DLP, the mechanical tunability of photocurable resins is crucial for optimizing properties to meet specific application demands.^[^ ^2^, ^3^, ^4^, ^5^ ^]^ This tunability is achieved by precisely controlling material compositions and ratios, enabling the development of hydrogels for medical applications and ionic or hyperelastic elastomers for soft robotics.^[^ ^6^, ^7^, ^8^ ^]^ Resin formulation for DLP typically involves blending various acrylate monomers and oligomers, with careful adjustments to the ratios and compositions of monomers and crosslinkers.^[^ ^9^, ^10^, ^12^, ^13^, ^14^ ^]^ Polyurethane‐based acrylates exhibit highly tunable mechanical properties, controlled by the composition of the polyol and chain extenders used during synthesis.^[^ ^12^, ^13^ ^]^ Among these properties, viscoelasticity plays a critical role in enabling rate‐dependent energy dissipation, thereby enhancing the energy absorption capacity of architected materials.^[^ ^11^, ^15^, ^16^, ^17^ ^]^ However, these materials encounter challenges in DLP processes, particularly in balancing viscoelastic damping and elastic modulus, which are often in a trade‐off relationship. Dynamic covalent bonds allow for energy dissipation without significantly increasing polymer chain length, thus maintaining the low viscosity required for DLP‐compatible polyurethane acrylate resins designed for self‐healing and recyclable applications.^[^ ^5^, ^11^, ^12^, ^18^, ^19^ ^]^ However, the elastic modulus in these formulations is typically limited to several MPa to a few tens of MPa, restricting their use in applications that require mechanically reinforced materials.

An alternative method for achieving mechanical tunability in photocurable resins occurs during the curing phase, utilizing multiple wavelengths of light or grayscale DLP (g‐DLP) technology to adjust light intensity.^[^ ^20^, ^21^, ^22^, ^23^, ^24^, ^25^, ^26^, ^27^, ^28^, ^29^ ^]^ The g‐DLP approach enables spatially controlled curing, providing precise control over chemical and mechanical properties. In g‐DLP printing, light intensity regulates the degree of monomer conversion and spatially controlled crosslinking density, which can be easily manipulated at the pixel level using a grayscale input image. As a result, single‐vat g‐DLP provides a cost‐effective solution for varying properties within a single resin material. Grayscale patterns in g‐DLP facilitate the simultaneous creation of stiff thermosets and stretchable soft organogels within a single layer.^[^ ^20^ ^]^ This technique also supports controlled buckling and enables programmable shape and motion deformation in soft robots, with further applications extending to 4D printing using UV‐curable shape memory polymers. It also enables the introduction of multiple colors in a single vat or enhances shape accuracy in a single‐batch process.^[^ ^30^ ^]^ Importantly, g‐DLP has also been shown to effectively reduce stress concentrations in printed structures. Forte et al. demonstrated that stress‐distribution‐based modulus gradient design strategies can mitigate stress concentrations, thereby delaying crack propagation and failure.^[^ ^24^ ^]^ More recently, Zhang et al. proposed a framework for g‐DLP‐printed soft robots that considers nonlinear material properties at the voxel level, allowing for reduced stress concentrations and programmable multimodal motion.^[^ ^26^ ^]^ In these approaches, the gradient design function for elastic modulus is determined by stress values at specific points rather than geometric positions, eliminating constraints based on structural shape while limiting variety. This underscores the need for optimization through versatile and finely tuned gradient design functions. Specifically, optimizing gradient design strategies to simultaneously address localized stress distribution and overall structural stiffness is essential for achieving greater design flexibility. Furthermore, current state‐of‐the‐art methods primarily focus on 2D designs^[^ ^24^, ^31^, ^32^ ^]^ with limited progress in 3D. Transitioning to 3D design necessitates an extensive number of elements, and continuously assigning varying material properties requires a large amount of input data, which increases the risk of errors and computational costs.^[^ ^33^ ^]^ To mitigate these issues, functional gradients of materials are discretized and assigned to groups of voxels; however, this may compromise the precision of grayscale patterns. Therefore, developing flexible gradient designs that function effectively with discrete distributions is essential to achieve a balance between time efficiency and accuracy in 3D design.

Machine learning (ML)‐driven structural optimization offers solutions for developing mechanically robust structures that utilize stress deconcentration through g‐DLP. Recent research has made significant advancements in ML‐driven multi‐objective optimization, which concurrently enhances two or more often conflicting mechanical properties in various materials and structures. Examples include lightweight nacre‐inspired composites with high strength and toughness,^[^ ^34^ ^]^ mechanically reinforced piezoelectric yarns that improve both ultimate tensile strength and failure strain,^[^ ^35^ ^]^ 3D‐printed architected materials for orthopedic implants that enhance elastic modulus and yield strength,^[^ ^36^ ^]^ and carbon nanolattices that provide ultrahigh specific strength while remaining lightweight.^[^ ^37^ ^]^ However, ML has yet to be explored for optimizing multiple mechanical properties in g‐DLP‐based 3D printing structures. To reduce stress concentration while enhancing effective stiffness, a ML‐driven multi‐objective optimization framework is necessary to optimize gradient design functions. This framework should account for structural behavior along with the nonlinear characteristics of materials, including elastic and plastic properties and failure behavior.

**Figure 1:**
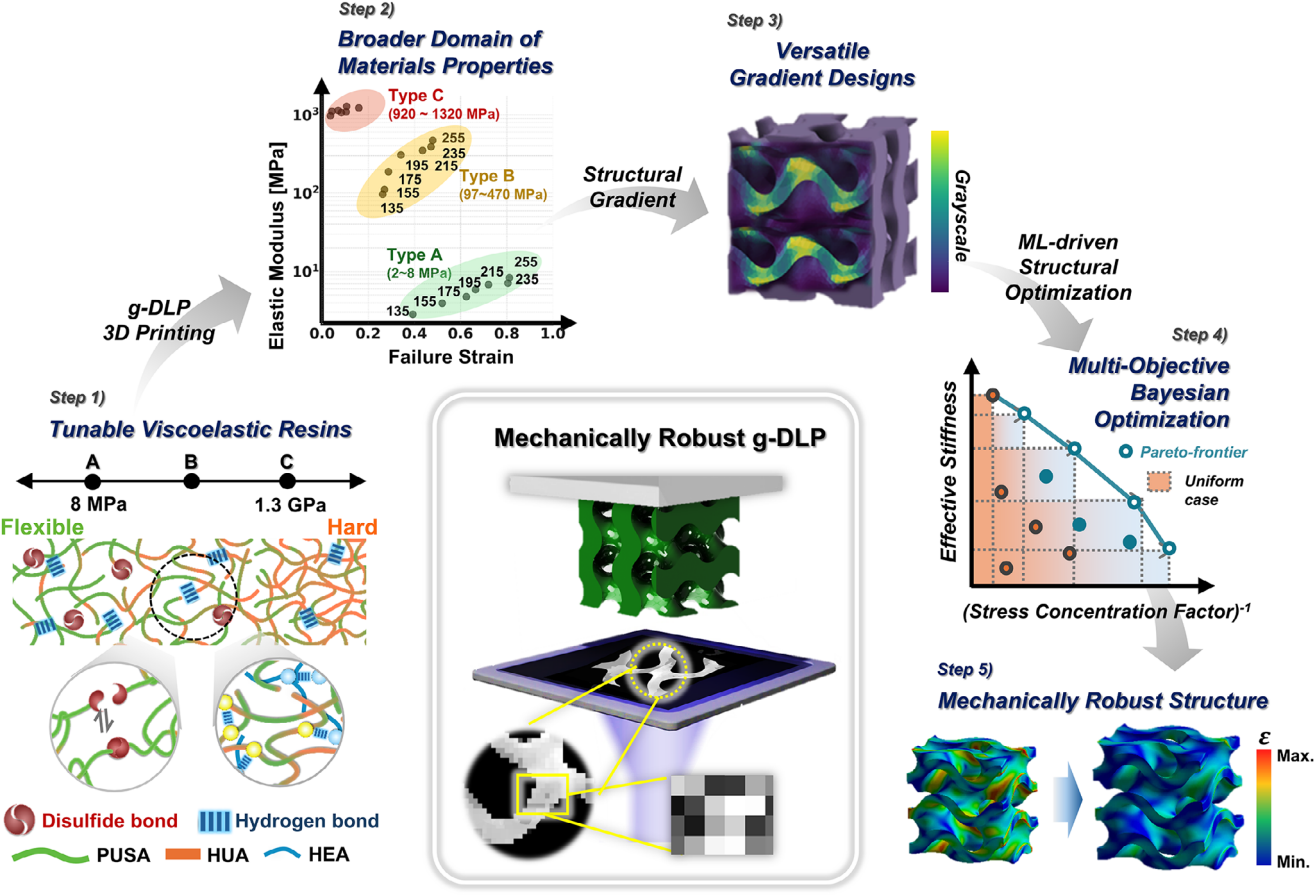
Overview of the material‐to‐structure design and fabrication strategies employed in this study for creating mechanically robust g‐DLP printed structures. Step 1: Development of highly tunable PUA with dynamic bonds to enhance viscoelastic damping. Step 2: g‐DLP 3D printing of viscoelastic PUSA‐HUA resins to achieve an expanded range of mechanical properties. Step 3: Implementation of a versatile gradient design strategy for the fabrication of arbitrary objects using g‐DLP. Step 4: Utilization of machine learning‐driven multi‐objective structural optimization to enhance two physical properties, reduce strain concentrations, and improve effective stiffness. Step 5: Optimization of gradient materials aimed at reducing stress concentrations and reinforcing structural integrity.

In this work, we develop mechanically robust g‐DLP printed structures through a synergistic strategy that integrates the synthesis of tunable polyurethane acrylate resin materials with ML‐driven multi‐objective structural optimization, leveraging elastic modulus gradient. This integrated approach enables simultaneous mitigation of stress concentrations through strain redistribution at the structure level and enhancement of energy dissipation at the material level, enabling mechanically robust structures. As illustrated in **Figure** **1**, our approach spans the entire process—from resin formulation to gradient structure design—enhancing the mechanical resilience of g‐DLP 3D‐printed products for biomedical and industrial applications. First, the material‐level design strategy involved developing polyurethane‐based resins with tunable mechanical properties and enhanced viscoelastic damping for g‐DLP 3D printing. Two urethane‐based acrylates with hard and soft compositions were synthesized to achieve tunability through compositional tailoring, while dynamic bonds responsible for enhanced viscoelastic damping are incorporated during synthesis. The resulting resin offers a broad elastic modulus range through compositional tailoring, enabling continuous property modulation during g‐DLP printing and facilitating gradient designs tailored to diverse structural and functional requirements. Second, a g‐DLP‐based gradient structure design and optimization framework was developed using ML‐driven multi‐objective optimization. This approach employed a Bézier curve‐based design function to generate diverse gradient structures while optimizing strain concentration factors and effective stiffness. The framework was further extended to 3D design, enhancing its applicability to complex real‐world structures and loading conditions. We applied this framework, integrated with the developed resin materials, to two representative cases: artificial knee cartilage—requiring flexibility, load‐bearing capacity, and impact absorption—and an automotive bumper beam—demanding sufficient stiffness and energy absorption.

## 2 Results and Discussion

### 2.1 Synthesis and Compositional Engineering of PUSA‐HUA Resins

**Figure 2:**
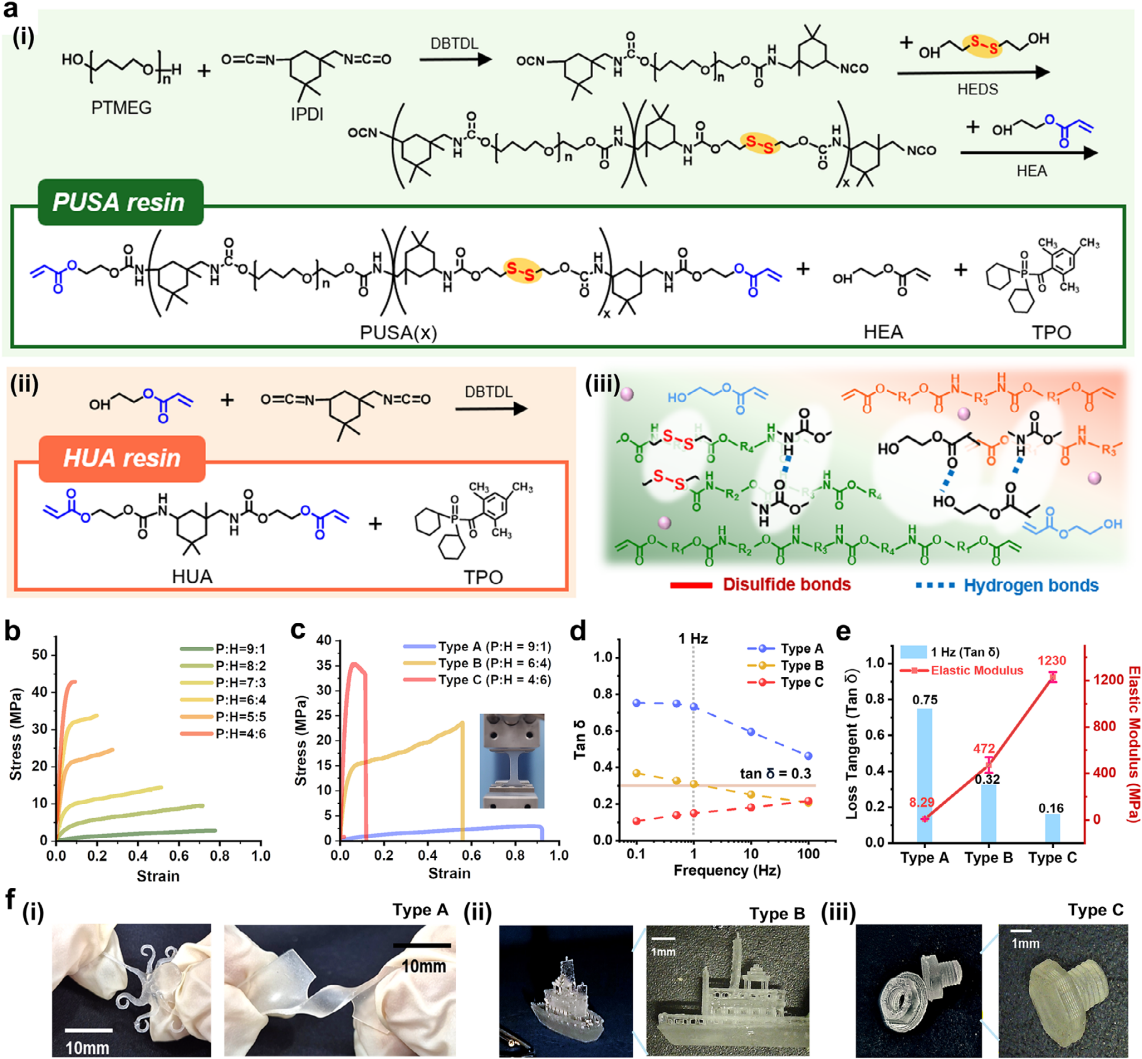
Development and characterization of PUSA‐HUA resins. a) Synthetic scheme and composition of resin components: (i) PUSA‐resin, (ii) HUA‐resin, and (iii) schematic illustration of PUSA‐HUA resin highlighting dynamic bonds. b) Stress‐strain curves of UV‐cured samples prepared by mold casting with varying PUSA‐resin to HUA‐resin (P:H) ratios. c) Stress–strain curves of DLP 3D‐printed PUSA‐HUA resins (Types A, B, and C). Inset: tensile testing setup. d) Frequency‐dependent loss tangent (tan δ) of PUSA‐HUA resins (Types A, B, and C) measured by dynamic mechanical analysis (DMA) at room temperature. e) Comparison of elastic modulus and tan δ at 1 Hz and room temperature for DLP 3D‐printed resins (Types A, B, and C). f) Photographs of DLP 3D‐printed structures using PUSA‐HUA resins: (i) Type A, (ii) Type B, and (iii) Type C. Scale bars in (i), (ii), and (iii) are 10 mm, 1 mm, and 1 mm, respectively.

To achieve a resin system with broad modulus tunability and integrated energy dissipation capability, we synthesized two types of polyurethane acrylates. The first type, polyurethane acrylate containing disulfide bonds (PUSA), was designed to introduce viscoelasticity through dynamic bonding. The second type, hydroxyethyl acrylate‐based urethane acrylate (HUA), was formulated to broaden the elastic modulus range. PUSA was synthesized using a stepwise addition polymerization process, as illustrated in **Figure** **2**a‐i, while HUA was produced via a one‐step synthesis method, depicted in Figure 2a‐ii. The PUSA formulations are denoted as PUSA(x), where *x* = 0.5, 1, or 1.5 represents the molar ratio of 2‐hydroxyethyl disulfide (HEDS) to polytetramethylene ether glycol (PTMEG). Dibutyltin dilaurate (DBTDL) served as the catalyst in both syntheses. The successful formation of PUSA and HUA was confirmed by the disappearance of the –NCO absorption peak at 2260 cm^−1^, as shown in Figure S1 (Supporting Information). Furthermore, ^1^H‐NMR spectra revealed the presence of specific structural characteristics, including disulfide‐adjacent methylenes (─CH_2_─S─S─) in PUSA and urethane‐linked groups (─CH_2_─NH─C(O)─) in both PUSA and HUA (Figures S2 and S3, Supporting Information).

Our strategy to enhance energy dissipation performance and enable DLP applicability involves incorporating dynamic disulfide bonds (─S─S─) into PUSA for DLP applications, which is controlled by varying the content of HEDS (Figure 2a‐i). In this study, we synthesized and characterized PUSA formulations with varying disulfide bond contents, as listed in Table S3 (Supporting Information), to assess their viscoelastic and mechanical properties using DMA, as presented in Figure S4a,b (Supporting Information). As the HEDS content increases, both the storage modulus and loss modulus increase. This is because the concentrations of disulfide bonds and urethane bonds both increase, leading to more extended polymer chains that enhance energy dissipation, while the urethane chains maintain structural rigidity. The effect of HEDS content on stress relaxation was evaluated in Figure S4c (Supporting Information). PUSA(0.5), which has the lowest disulfide bond content and the shortest molecular chains, exhibited limited chain mobility and showed relaxation times 8.5 and 12.7 times longer than those of PUSA(1) and PUSA(1.5), respectively. Thus, we eliminated PUSA(0.5) from further consideration as a candidate formulation. To validate that the energy dissipation and viscoelastic performance in PUSA arise from the reversible nature of disulfide bonds, we compared PUSA with a polyurethane acrylate chain extended with 1,4‐butanediol (BDO), referred to as PUBA. BDO is a common chain extender in flexible polyurethane acrylates (Table S2 and Figure S5, Supporting Information). As shown in Figure S5 (Supporting Information), PUSA exhibited higher loss modulus values and higher loss tangent (tan δ) at T_g_ than PUBA, confirming the effectiveness of the dynamic bonds.

Although introducing HEDS proved effective in increasing energy dissipation, the resulting formulations exhibited high viscosity and required the addition of a diluent acrylate. We mixed 50 wt.% of 2‐hydroxyethyl acrylate (HEA), a photocurable monomer and end‐capping agent, with PUSA. In addition to improving processability, HEA plays an important role in forming dynamic bonding interactions through its hydroxyl groups, which can participate in hydrogen bonding within the polymer network (Figure 2a‐iii). These interactions, along with their flexible nature, contribute to enhanced energy dissipation, as evidenced by the increase in loss modulus upon HEA incorporation (Figure S6 and Table S3, Supporting Information). For viscosity comparison, we excluded PUSA(0.5), which had the weakest energy dissipation performance, and compared the pre‐mixed (diluted) forms of PUSA(1) and PUSA(1.5). PUSA(1.5) exhibits a viscosity of 7160 Pa s, even in a diluted solution, which is too high for effective DLP 3D printing due to its long chain length. Therefore, PUSA(1) was selected as the most suitable formulation. Additionally, we incorporated 2 wt.% of diphenyl(2,4,6‐trimethylbenzoyl)phosphine oxide (TPO) as a photoinitiator to create a photocurable resin, referred to as PUSA‐resin, with optimal viscosity and curing capability (Figure 2a‐i). The detailed composition ratios for the finalized PUSA‐resin formulation are summarized in Table S4 (Supporting Information). For the photocurable HUA‐resin, no additional diluent was needed; only TPO was added at a concentration of 2 wt.% relative to the weight of HUA, as outlined in Figure 2a‐ii and summarized in Table S5 (Supporting Information).

A key strategy for constructing broadly tunable polyurethane acrylate resin systems for DLP applications is the precise control of the composition ratio between PUSA‐resin and HUA‐resin. In this mixture, the PUSA‐resin imparts flexibility and damping,^[^ ^11^, ^15^ ^]^ while the HUA‐resin confers high stiffness. To select the optimal composition ratio for DLP 3D printing, preliminary screening of mechanical properties was conducted by casting the mixture into molds, followed by UV curing. Figure 2b shows the stress–strain curves of UV‐cured PUSA‐HUA resins with various PUSA‐resin:HUA‐resin (P:H) ratios, and the measured mechanical properties are presented in Table S6 (Supporting Information). These results demonstrate that, by finely adjusting the composition ratio, the stress–strain curves gradually change, covering a wide range of mechanical properties. For example, a composition with a high HUA‐resin content (P:H = 4:6) exhibited high tensile strength (55.6 MPa) and modulus (1494 MPa), but displayed a low elongation at break (0.069) due to increased crosslink density. Conversely, a composition with a high PUSA‐resin content (P:H = 9:1) showed much higher elongation at break (0.76) and excellent flexibility, with lower strength (7.42 MPa) and modulus (64.6 MPa). Overall, these results indicate that the resin's performance can be finely tuned by precisely adjusting the P:H ratio, highlighting its suitability for a wide range of applications. Furthermore, this serves as a useful reference for selecting compositions in the DLP 3D printing step.

Based on these results, we selected three representative resin formulations to explore a wide range of stiffnesses. These formulations are defined as Type A (flexible, P:H ratio of 9:1), Type B (moderate stiffness, 6:4), and Type C (high stiffness, 4:6). Using these resins, we fabricated identical dogbone specimens via DLP 3D printing for tensile testing, which produced the stress‐strain curves shown in Figure 2c. Collectively, these resins cover a broad modulus range from 8.3 MPa to 1.2 GPa, establishing a versatile material platform for gradient structure design. A comparison with the initial cast samples reveals that all 3D‐printed formulations exhibit a lower elastic modulus but higher failure strain, resulting in enhanced overall toughness (Table S7, Supporting Information).

Building upon these results, we further analyzed the viscoelastic damping performance of the selected resins. As shown in Figure S7 (Supporting Information), DMA measurements were conducted in a stepped frequency sweep mode over the range of 0.1–100 Hz at five discrete frequencies (0.1, 0.5, 1, 10, and 100 Hz). This evaluation aimed to examine the intrinsic damping behavior of the polymers and their sensitivity to dynamic mechanical loads. As depicted in Figure 2d, the tan δ values decreased in the order: Type A > Type B > Type C, corresponding to increasing stiffness. According to the widely accepted criterion for damping materials (tan δ ≥ 0.3),^[^ ^38^ ^]^ Type A exceeded this threshold at all frequencies, demonstrating substantial damping capability. Type B surpassed it only at low frequencies, while Type C remained below 0.3 across all frequencies. These results indicate that, at room temperature (RT), Type A and Type B resins demonstrate potential as effective damping materials. Figure 2e presents bar graphs of the damping factor (tan δ) at 1 Hz and RT, alongside a line graph of the elastic modulus. This summary combines data from Figure 2c,d. Although there is a typical trade‐off between loss tangent (tan δ) and elastic modulus (E), the tan δ values for our developed resin materials are relatively high—especially for Type C, which exhibits a tan δ of ≈0.16 and an elastic modulus (E) of 1230 MPa. To emphasize this point, the properties of the PUSA‐HUA resins are marked with star symbols on the Ashby chart of loss coefficient versus elastic modulus (Figure S8, Supporting Information), allowing for a clearer comparison between existing materials and our developed PUSA‐HUA resins. Importantly, while the chart highlights the usual trade‐off—where higher stiffness (elastic modulus) typically corresponds to lower damping performance (loss coefficient, tan δ)—our developed resins maintain a relatively high loss coefficient even as their modulus increases. This indicates that our materials can achieve a good balance of stiffness and damping performance, defying the conventional trade‐off. These properties arise from dynamic bonds in the resin matrix, primarily disulfide (─S─S─) bonds from the chain extender in PUSA, along with numerous urethane (NH─COO) bonds and hydroxyl (─OH) groups from the HEA diluent (Figure 2a‐iii). The presence of these hydroxyl and disulfide bonds is confirmed by FTIR (Fourier Transform Infrared Spectroscopy) and Raman spectra (Figure S9, Supporting Information), as shown in bar graphs alongside the elastic moduli.

To further evaluate the suitability of the custom PUSA‐HUA resin for DLP processing, we characterized its viscosity and photopolymerization behavior (Figure S10, Supporting Information). Rheological analysis revealed that the liquid resin has an appropriate viscosity and exhibits shear‐thinning characteristics, confirming good compatibility with DLP printing. Additionally, we determined an optimal curing depth range that corresponds to the chosen layer thickness. The photopolymerization behavior of each resin formulation was further evaluated through Jacobs’ curve analysis. Figure 2f and Figure S11 (Supporting Information) demonstrate the resin's capability for precise free‐form fabrication via DLP.

### 2.2 Applicability of PUSA‐HUA Resins in g‐DLP

**Figure 3:**
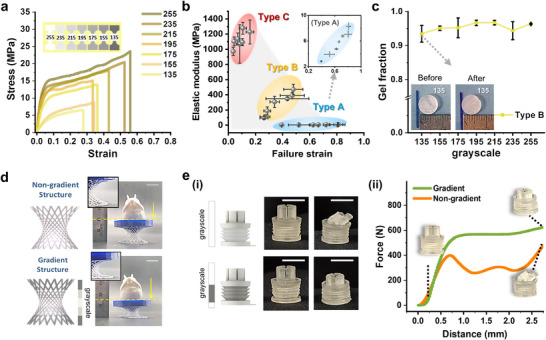
Extended material properties of PUSA‐HUA resins via g‐DLP and applications of g‐DLP 3D printing. a) Stress‐strain curves of g‐DLP 3D‐printed resin Type B at various grayscale levels, ranging from 135 to 255. b) Comparison of the mechanical property ranges of g‐DLP‐printed resins Types A, B, and C, showing failure strain versus elastic modulus with error bars. Inset: enlarged view of the region corresponding to Type A. c) Gel fraction plot for g‐DLP 3D‐printed resin Type B. Inset: photographs of the sample (135) before and after the gel fraction test. d) Illustration of non‐gradient and gradient structures of a hyperboloid lattice, along with images of the g‐DLP 3D‐printed structure under compression. e) (i) Illustration of non‐gradient and gradient structures of a motor damper, including comparisons of printed non‐gradient and gradient structures before and after compression. (ii) Force‐displacement curves of gradient and non‐gradient structures under compression at a rate of 1 mm min^−1^. All scale bars represent 10 mm.

Next, we aimed to quantitatively demonstrate the broad expansion of mechanical properties, including the elastic modulus, through spatial gradients within a single vat process using g‐DLP printing for our developed resin. In 8‐bit grayscale images, each pixel represents an intensity value from 0 (black) to 255 (white), with higher values indicating greater light intensity in DLP 3D printing.^[^ ^39^ ^]^ We used grayscale intensities ranging from 135 to 255 in ≈20‐unit increments, as illustrated in the inset of **Figure** **3**a, representing seven grayscale levels to ensure consistent and reliable printing for the dogbone specimens. The light intensities corresponding to each grayscale level are provided in Table S8 (Supporting Information). Figure 3a shows that the stress‐strain curves of g‐DLP 3D‐printed specimens using Type B resin demonstrate moderate stiffness, with elastic modulus values ranging from 96.8 MPa at grayscale 135 to 472 MPa at grayscale 255, achieving a variation of approximately 4.9 times within a single material. Type A resin shows various low elastic moduli from 2.83 MPa at grayscale 135 to 8.29 MPa at grayscale 255 (Figure S12a, Supporting Information), while Type C resin demonstrates high stiffness, with values ranging from 972 MPa to 1230 MPa (Figure S12c, Supporting Information). The elastic modulus, failure strain, tensile strength, and toughness for Types A, B, and C resins at various grayscale levels are summarized in Tables S9–S11 (Supporting Information). This finding is significant as it underscores an approach that facilitates smooth transitions of properties from several MPa to over 1 GPa, as illustrated in Figure 3b, validating the synergistic effect of g‐DLP printing in substantially expanding the range of properties for tailor‐made PUSA‐HUA resins.

In g‐DLP printing, resins typically exhibit lower gel fraction at reduced light intensities due to their dependence on light‐driven crosslinking density.^[^ ^40^ ^]^ Although the degree of conversion varies across grayscale levels, with a relative difference of ≈38% between the maximum (grayscale 255) and minimum (grayscale 135) (Figure S13, Supporting Information), acetone swelling measurements indicate that our PUSA‐HUA resins achieved gel fraction exceeding 90% under low grayscale conditions (135). This demonstrates their chemical stability and minimal shape changes after light exposure (Figure 3c; Figure S14, Supporting Information). This enhanced stability arises from the synergistic effects of polyurethane polymers, which promote adequate entanglement, along with the crosslinked structure of acrylates and the abundance of ‐OH groups.

Additionally, the ability to adjust elastic stiffness using g‐DLP is exemplified by a gradient‐embedded hyperboloid lattice structure printed with Type C resin (Figure 3d). Fine lattice beams typically restrict control over material properties through thickness adjustment alone. However, applying lower grayscale values to the top and bottom sections enables effective property modulation within the same hyperboloid lattice, enhancing deformability compared to a non‐gradient equivalent under the same weight. Figure 3e illustrates another example printed using g‐DLP with Type C resin, featuring a damper structure with a rotating shaft mount. The upper section of the damper typically encases the motor shaft and has a thin, protruding design that can lead to stress concentration. To mitigate this issue, we engineered the upper part for high stiffness while allowing the body to maintain lower stiffness, thus protecting vulnerable areas through a carefully designed grayscale pattern. In Figure 3e‐i, the non‐gradient configuration shows that the weak fixed part deforms first, resulting in reduced toughness, whereas the gradient configuration enables the damping region to deform initially due to its reduced stiffness, thereby protecting the weak fixed part and enhancing overall robustness under compression. The force‐displacement curves of the damper structures under compression, presented in Figure 3e‐ii, quantitatively confirm that the spatially modulated elastic stiffness achieved through g‐DLP in the gradient configuration improves the overall toughness and structural robustness of the entire damper. Additionally, Figure S15 (Supporting Information) presents the design and behavior of various gradient‐applied monostable beam structures printed with g‐DLP using Type B resin as another example, showing that the deformation shape of the beam could be controlled by tuning the grayscale distribution. To further evaluate the performance and reliability of our gradient printing method, we examined dimensional accuracy and fidelity across different grayscale levels using Type B resin. This assessment, based on cylindrical structures with various aspect ratios and octet truss designs (Figure S16, Supporting Information), confirmed consistent print quality throughout the entire grayscale range.

### 2.3 Design of Gradient Structures via ML‐Driven Multi‐Objective Optimization

**Figure 4:**
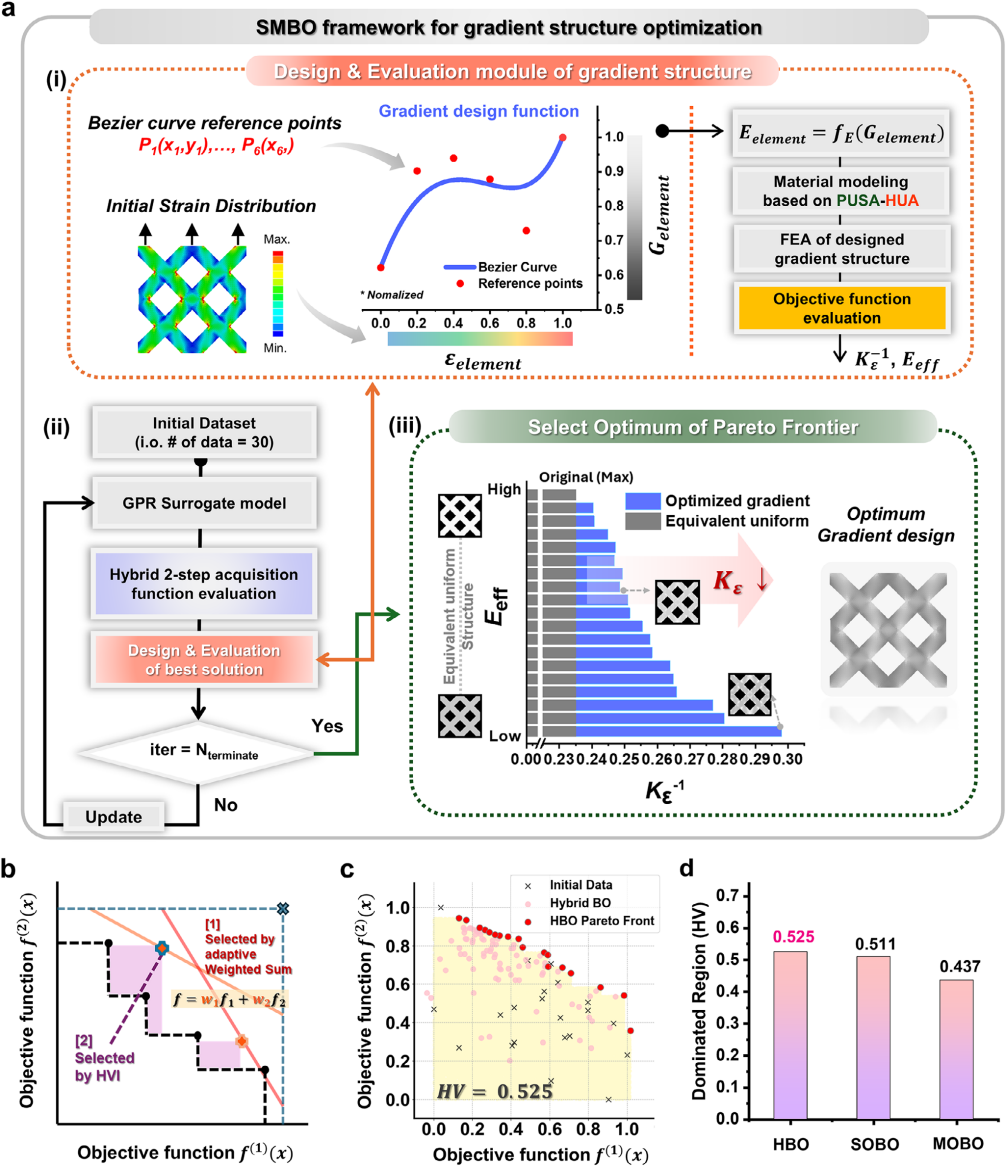
Multi‐objective SMBO framework for gradient structure optimization to simultaneously mitigate stress concentrations and enhance effective stiffness. a) (i) Design and evaluation module for unit gradient structures, where reference points of the Bézier curve generate the gradient design function. The initial strain value is mapped to a grayscale value for each element, and the G_element_ value is converted into an E_element_ for FEA, followed by an evaluation of the objectives. (ii) Flowchart illustrating the SMBO process with a hybrid two‐step acquisition function in the HBO algorithm utilizing a GPR surrogate model. (iii) Selection of the optimal design from Pareto frontiers displayed in the SCF^−1^ and E_eff_ plots. b) Schematic illustration of the hybrid Bayesian optimization (HBO) algorithm. c) Optimization results showing the pareto frontiers generated by HBO and the corresponding hypervolume (HV) metrics; the yellow area represents the region dominated with respect to the reference point, which defines the hypervolume. d) Comparison of dominated region values obtained from the three optimization algorithms.

Building on advancements in customizable and mechanically robust materials, we have developed an ML‐driven multi‐objective framework that generates gradient structural designs for DLP 3D printing, aimed at effectively reducing stress concentration and enhancing stiffness beyond the constraints of intrinsic material properties. **Figure** **4**a presents the flowchart for the sequential model‐based Bayesian optimization (SMBO)‐based gradient design optimization framework established in this work. Figure 4a‐i depicts the design of the gradient design function, derived from Bézier curve reference points and the strain distribution within a 2D lattice structure used as an example case subjected to loading. The Bézier curve reference points shape the gradient design function, while the strain distribution maps the initial strain values (ɛ_element_) obtained from finite element analysis (FEA) under loading to a grayscale value (G_element_) (ranging from 135 to 255) denotes the assigned grayscale intensity for each element. Our approach is innovative as it employs Bézier curves, offering greater flexibility compared to previously reported exponential and sigmoid functions,^[^ ^37^, ^41^ ^]^ thus facilitating efficient optimization. Further details on the structured mapping from initial strain to grayscale to elastic modulus, including the Bézier curve boundary conditions and their necessity (Figure S17, Supporting Information), are provided in Section S5 (Supporting Information). Importantly, the imposed boundary condition, which assigns the highest elastic modulus to the region of maximum strain, plays a role in effectively reducing maximum stress (Figure S18, Supporting Information). The grayscale mapping is subsequently used to adjust the elastic modulus of each element based on experimental measurements (Figure S19, Supporting Information). The generated gradient structure is then analyzed through FEA to assess the multi‐objective functions, f^(1)^(x) and f^(2)^(x). The first objective function, f^(1)^(x), represents the inverse of the strain concentration factor (SCF), a metric quantifying how much localized strain exceeds the nominal strain, as defined in Equation 1:^[^ ^32^, ^42^ ^]^

(1) $$f^{\left(1\right)}\left(x\right)=\mathrm{SC}F^{-1}={K_{\varepsilon }}^{-1}=\frac{\varepsilon_{\mathrm{nominal}}}{\varepsilon_{\max}}$$

where K_ɛ_ is the strain concentration factor, ɛ_max_ is the maximum principal strain, and ɛ_nominal_​ is the nominal strain, calculated by ɛ_nominal_ =  ΔL/L_0_​, with ΔL representing the applied displacement and L_0_​ the initial length of the structure. It is important to note that strain concentration is used because it directly reflects local deformation behavior, independent of material modulus variations. In uniform materials, stress concentration factors are typically employed to assess local stress amplification caused by geometry. However, in gradient structures with spatially varying elastic modulus, strain concentration is more appropriate since stress values are directly influenced by local stiffness. While many previous studies associate the reduction of strain concentration—and thus delayed failure at the material level—with the broader goal of stress concentration mitigation,^[^ ^24^, ^32^ ^]^ analyzing stress concentration via stress concentration factor becomes less relevant here. This is because nominal stress, derived from the global stiffness, also varies with the modulus gradient. Consequently, even if maximum stress at critical regions is reduced, the calculated stress concentration factor may not decrease correspondingly, potentially giving a misleading impression of stress concentration mitigation. As shown in Figure S18 (Supporting Information), the stress distribution can be complex, and embedding high‐modulus materials in the design effectively reduces maximum stress at key locations, but assessing the effectiveness solely through the stress concentration factor may be misleading in gradient structures. The second objective function, f^(2)^(x) represents the effective stiffness (E_eff_), defined in Equation 2 as the average slope of the stress‐strain curve within the elastic deformation region, measured at the specified displacement.

(2) $$f^{\left(2\right)}\left(x\right)=E_{\mathrm{eff}}=\frac{\mathrm{Force}}{\mathrm{Displacement}}\left[N\;mm^{-1}\right]$$

Figure 4a‐ii shows the iterative SMBO flowchart, where 30 Bézier curves are provided as input data, while the two evaluated objective functions, E_eff_ and SCF^−1^, act as output data for training a Gaussian process regression (GPR)‐based surrogate model within the SMBO framework. Examples of various generated Bézier curves are shown in Figure S17 (Supporting Information). Subsequently, Hybrid Bayesian optimization (HBO) process generates solutions, and each solution is evaluated at every step using FEA for updating the GPR model.

Notably, we developed the HBO algorithm as our multi‐objective optimization process, since a more customized model was necessary to address the challenges posed by two strongly trade‐off objective functions, f^(1)^(x) and f^(2)^(x), along with the inherent upper limit of f^(2)^(x) (E_eff_). The HBO algorithm combines strategies from both single‐objective Bayesian optimization (SOBO) (Figure S20a, Supporting Information) and multi‐objective Bayesian optimization (MOBO) (Figure S20b, Supporting Information). Its two‐step evaluation process is illustrated in Figure 4b. First, an adaptive weighted‐sum‐based SOBO employs Expected Improvement (EI) as the acquisition function to identify a set of promising solution candidates by exploring diverse regions through recurrent adjustments of weight ratios.^[^ ^43^ ^]^ Next, Expected Hypervolume Improvement (EHVI)‐based MOBO evaluates these candidates and selects the final solution that maximizes the acquisition function, EHVI.^[^ ^44^ ^]^ The detailed flowchart for the two‐step decision process of HBO is provided in Figure S21 (Supporting Information). In this framework, the construction of the GPR surrogate model is summarized in Table S12 (Supporting Information), and its predictive performance was validated throughout the optimization process, as shown in Figure S22 (Supporting Information). The GPR model demonstrated reliable performance across iterations, with the objective functions f^(1)^(x) and f^(2)^(x) reaching coefficients of determination (R^2^) above 0.98 and 0.96, respectively, after 20 iterations, confirming the effective learning and exploration capabilities of the Bayesian optimization process.

Figure 4c shows the Pareto front obtained via the HBO process, where the Hypervolume (HV)—which indicates the volume of the dominated region in the objective space—demonstrates well‐distributed solutions achieved through effective exploration. In comparison, Figure 4d summarizes the results of SOBO, MOBO, and HBO, with HBO achieving the largest HV (0.511). Additional details, including the distribution of solutions generated by SOBO and MOBO, are available in Figure S20 (Supporting Information).

Once the termination condition of HBO is met, the user can define the final optimum from the Pareto‐optimal set (Figure 4a‐iii). The bars in the graph represent various effective stiffness (E_eff_) values along the vertical axis, while the horizontal axis indicates the degree of SCF reduction. These Pareto‐optimal solutions offer a range of feasible stiffness values and their corresponding optimal structures, allowing us to select options based on our specific objectives. Although a fundamental trade‐off exists between the two objectives, our approach consistently improves stress concentration mitigation across various cases. A grayscale mask for the optimized gradient 2D lattice structure is demonstrated as a sample case in Figure 4a‐iii. The results of the entire Pareto frontiers and gradient optimization, along with corresponding grayscale mask images for g‐DLP printing of 2D lattice structures, rounded hole unit cells, and star‐shaped hole unit cells, are also provided in Figures S23–S25 (Supporting Information) to illustrate the diverse applicability of the developed HBO‐based SMBO framework. Additionally, Table S13 (Supporting Information) summarizes the generated data, and Table S14 (Supporting Information) lists the reference points for the gradient design functions, obtained from the optimal solutions of the three unit cells based on Bézier curves. Figure S26 (Supporting Information) shows that the star‐shaped hole achieves the highest SCF reduction, reaching up to 83.6%, while the rounded hole achieves 46.8%, indicating that the reduction of SCF is highly dependent on intrinsic structural geometry.

### 2.4 Numerical and Experimental Comparison of Gradient 2D Unit Cell Structures

**Figure 5:**
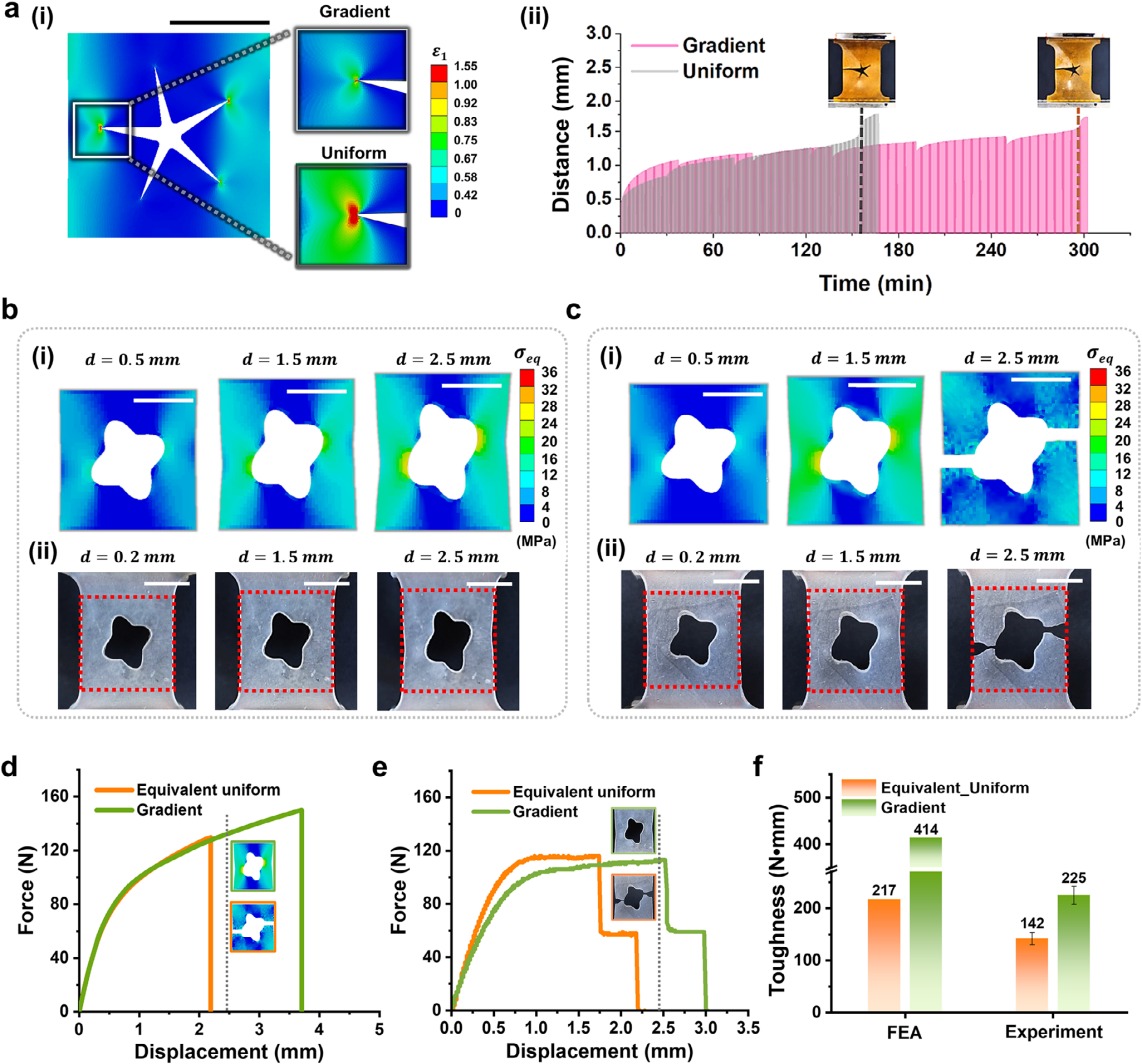
Numerical and experimental evaluations of the 2D star‐shaped hole unit cell structure and the rounded‐hole unit cell structure. For the star‐shaped hole unit cell structure: a) (i) Strain distribution plots comparing the gradient and uniform structures, with the color bar indicating maximum principal strain (𝜀_₁_). (ii) Cyclic test results comparing the equivalent uniform structure and the optimized gradient structure derived from the SMBO framework. For the rounded‐hole unit cell structure: Comparison of b) the equivalent uniform structure and c) the optimized gradient structure derived from the SMBO framework: (i) Equivalent stress distribution plots from FEA at three displacement steps, with the color bar indicating equivalent stress during tensile loading. (ii) Images depicting deformation at corresponding displacement steps during tensile testing. Force–displacement curves from tensile tests in d) FEA and e) experimental results. Inset: The deformation state of each structure at the same displacement, marked by gray lines. f) Comparison of toughness between the equivalent uniform and gradient structures, based on both FEA and experimental results. All scale bars represent 10 mm.

**Figure** **5** presents numerical and experimental validation of the optimized gradient structure derived from the HBO‐based SMBO framework. To investigate its effectiveness in mitigating strain concentrations, we selected a 2D star‐shaped hole unit cell, known for exhibiting severe stress localization, as a representative example. For comparison, an equivalent uniform structure with matching effective stiffness—achieved through grayscale modulation and identical geometry—was also fabricated.

As shown in the FEA results (Figure 5a‐i), the optimized gradient structure markedly reduced the maximum equivalent strain from 1.99 to 1.02, corresponding to an approximate 49% reduction relative to the uniform structure. To experimentally validate this strain concentration mitigation effect, cyclic tensile loading tests were conducted on g‐DLP printed samples (Figure 5a‐ii), fabricated using Type B resins (Figure S27, Supporting Information). During testing, the specimens were repeatedly loaded up to a peak load of 4 N, with the displacement returning to zero after each cycle. The initial test consisted of 500 repetitions, followed by repeated tests of 1000 cycles each, with a 10‐minute rest period between each set. The results demonstrated that the gradient structure exhibited superior fatigue performance, enduring ≈5800 cycles before failure, whereas the uniform structure failed after about 2600 cycles. This indicates that spatially graded modulus distributions effectively delay failure initiation and can significantly extend fatigue life under cyclic loading.

Second, we selected a 2D rounded‐hole unit cell made from Type B resin as an example of a geometry more tolerant to stress concentration. Figure 5b shows deformation images of the equivalent uniform structure under both FEA and experimental conditions as a function of displacement (d). Figure 5c presents the corresponding results for the gradient structure under the same conditions. In both cases, increasing displacement causes strain concentration at the two most protruding points of the internal hole; however, the gradient structure more effectively mitigates this effect. Consequently, the uniform specimen fractured at the two stess concentration points at the respective displacements during FEA (*d* = 0.55 mm, Figure 5b‐i) and experiments (*d* = 2.5 mm, Figure 5b‐ii). In contrast, no fracture was observed in the optimized gradient specimen at the same displacement (Figure 5c‐i,ii). The g‐DLP mask image used, along with the printed structures and detailed experimental photographs, can be found in Figure S28 (Supporting Information). The corresponding force‐displacement curves from both FEA (Figure 5d) and tensile experiments (Figure 5e) further illustrate these fracture behaviors. In the simulations, a hyperelastic material model was adopted to accurately capture large deformation behavior and the nonlinear stress increase observed at high strains in polymers, which do not exhibit a clear yield behavior. To reflect these material characteristics, experimentally derived stress–strain curves for at different grayscale levels were directly incorporated into the FEA model. The detailed curves and modeling approach are provided in Figure S19 (Supporting Information).

Both experimental and numerical analyses successfully captured the nonlinear deformation response, particularly the increased toughness of the gradient structure over the equivalent uniform specimen. Specifically, as shown in Figure 5f, FEA predicted toughness values of 414 N mm and 217 N mm for the gradient and uniform structures, respectively, corresponding to a 91% increase in the gradient structure compared to the equivalent uniform structure. In the experimental results, mean toughness values of 225 N mm and 142 N mm were obtained at a strain rate of 5 mm s^−1^ for the gradient and uniform structures, respectively, corresponding to approximately a 60% enhancement. Additional tests at lower strain rates (Figure S28e, Supporting Information) revealed that the toughness of the gradient structure significantly increased from 225 N mm at 5 mm s^−1^ to 387 N mm at 0.5 mm s^−1^. At this lower strain rate, the gradient structure demonstrated ≈72% higher toughness than the uniform specimen. These findings show that the gradient structure performs effectively under both lower (0.5 mm s^−1^) and higher (5 mm s^−1^) loading speeds. To simulate fracture behavior in gradient materials exhibiting various nonlinear responses, the FEA modeling prioritized computational efficiency and numerical stability, which limited the ability to capture all strain rate‐dependent effects. Accordingly, a hyperelastic model was employed, which successfully captured large deformation behavior and strain redistribution mechanisms; however, it did not account for strain rate dependency and energy dissipation. Despite certain simplifications, the FEA model effectively captures the overall trend of toughness enhancement driven by spatial gradients, showing its applicability for predicting the mechanical performance improvement of gradient structures.

To further verify the mechanical performance of a gradient structure, uniform specimens with various grayscale levels (135–255) were tested alongside the gradient structure (Figure S29, Supporting Information). While uniform specimens with higher grayscale exhibited increased toughness, the gradient structure consistently outperformed them, demonstrating superior energy absorption even compared to the uniform specimen at grayscale 255. These results indicate that the optimized gradient design is likely to delay crack initiation under cyclic loading and enhance toughness under uniaxial tension, depending on the structural geometry. This implies that ML‐driven strain concentration‐mitigating strategies offer promising potential to improve mechanical robustness through various toughening mechanisms.

### 2.5 Mechanically Robust 3D Structures for Biomedical and Automotive Applications

**Figure 6:**
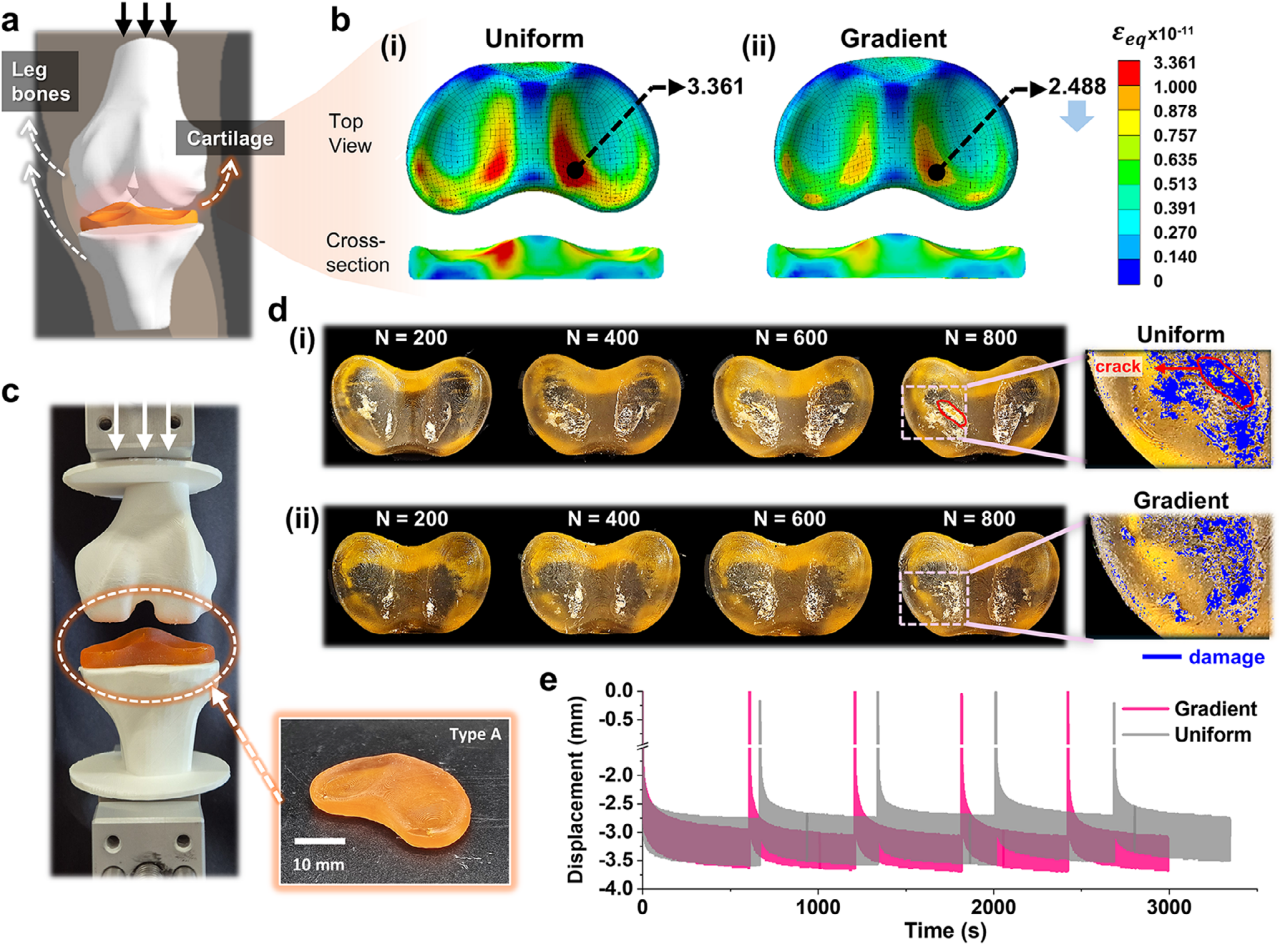
3D application of gradient design utilizing the SMBO framework with PUSA‐HUA resin: artificial human knee cartilage. a) Schematic representation of human knee cartilage under compression. b) Strain distribution plots for (i) uniform and (ii) gradient structures, with the color bar indicating equivalent strain (ɛ_eq_). c) Experimental setup featuring g‐DLP‐printed cartilage for cyclic compression testing. d) Damage evolution in specimens during cyclic compression testing of (i) uniform and (ii) gradient structures. e) Time‐domain displacement curves obtained from cyclic compression testing.

Lastly, we transformed the 2D‐based gradient design optimization framework depicted in Figure 4 into a 3D‐based design framework to enhance its practical applications. Unlike the simplified 2D environment, the realistic 3D setting introduces challenges for computational convergence due to loading conditions involving contact, which complicates the application of continuous gradients.^[^ ^26^ ^]^ Additionally, a discrete approach may not adequately capture extreme values at stress concentration points, which are statistically classified as outliers.^[^ ^45^ ^]^ To address these challenges, our framework effectively integrates 100 discrete gradient levels, allowing for rapid variations in the gradient within localized areas through the use of arbitrary Bézier functions, thereby demonstrating its efficacy in a 3D context. Furthermore, when assigning grayscale values to elements, we utilized the maximum strain rather than the average strain to more accurately reflect the stress concentration points. To demonstrate the diverse applicability in biomedical and industrial fields, we selected knee cartilage and automotive bumper structures as representative examples. For artificial cartilage, we chose Type A resin, which offers a modulus range of 8.3 to 2.8 MPa via g‐DLP printing. This range is suitable for knee cartilage, which requires a material that is flexible enough for shock absorption while still providing sufficient load‐bearing capacity. The knee cartilage serves as an energy absorber between the knee joint bones, which possess various curvatures (**Figure** **6**a).^[^ ^46^, ^47^ ^]^ As shown in Figure 6b‐i, the distribution of equivalent strain (ɛ_eq_) reveals an inhomogeneous stress profile that includes areas of undesirable high strain concentration under loading in a uniform structure. By applying gradient design optimization to the knee cartilage structure (Figure S30, Supporting Information), we achieved a reduction in the maximum equivalent strain (max(ɛ_eq_)) value to 2.488e^−11^, which represents a 28% decrease compared to the uniform structure (3.361e^−11^), as illustrated in Figure 6b‐ii. Figure 6c presents the cartilage structure printed using g‐DLP based on the gradient design (Figure S31, Supporting Information). We incorporated this structure into a 3D‐printed knee joint simulation system to conduct cyclic compression tests, with a loading range from 50 to 500 N, repeated in increments of 200 cycles, and a 5 min resting period between each set (Figure S32, Supporting Information). The progression of cartilage damage with respect to the number of cycles is depicted in Figure 6d, where we compare the equivalent uniform structure (Figure 6d‐i) with the gradient structure (Figure 6d‐ii). Throughout all repetitions, the gradient structure exhibited a significantly reduced damage area compared to the uniform structure. Notably, at N = 800, which N is the number of cycles, the uniform structure demonstrated not only surface damage but also the formation and propagation of vertical cracks, while the gradient structure showed no visible cracking. This indicates that gradient design optimization enhances the redistribution of strain in 3D structures, effectively mitigating unwanted strain concentrations under compressive loads. The results of displacement over time throughout the entire cyclic curve are illustrated in Figure 6e. The initial displacements for both the gradient and uniform structures were comparable, indicating similar effective stiffness. However, upon comparing the peak‐to‐peak displacements at the endpoint, the gradient structure exhibited an ≈25% reduction in displacement compared to the uniform structure. This suggests that, although both structures possess comparable stiffness under the same loading conditions, the gradient structure stabilizes deformation more effectively.

**Figure 7:**
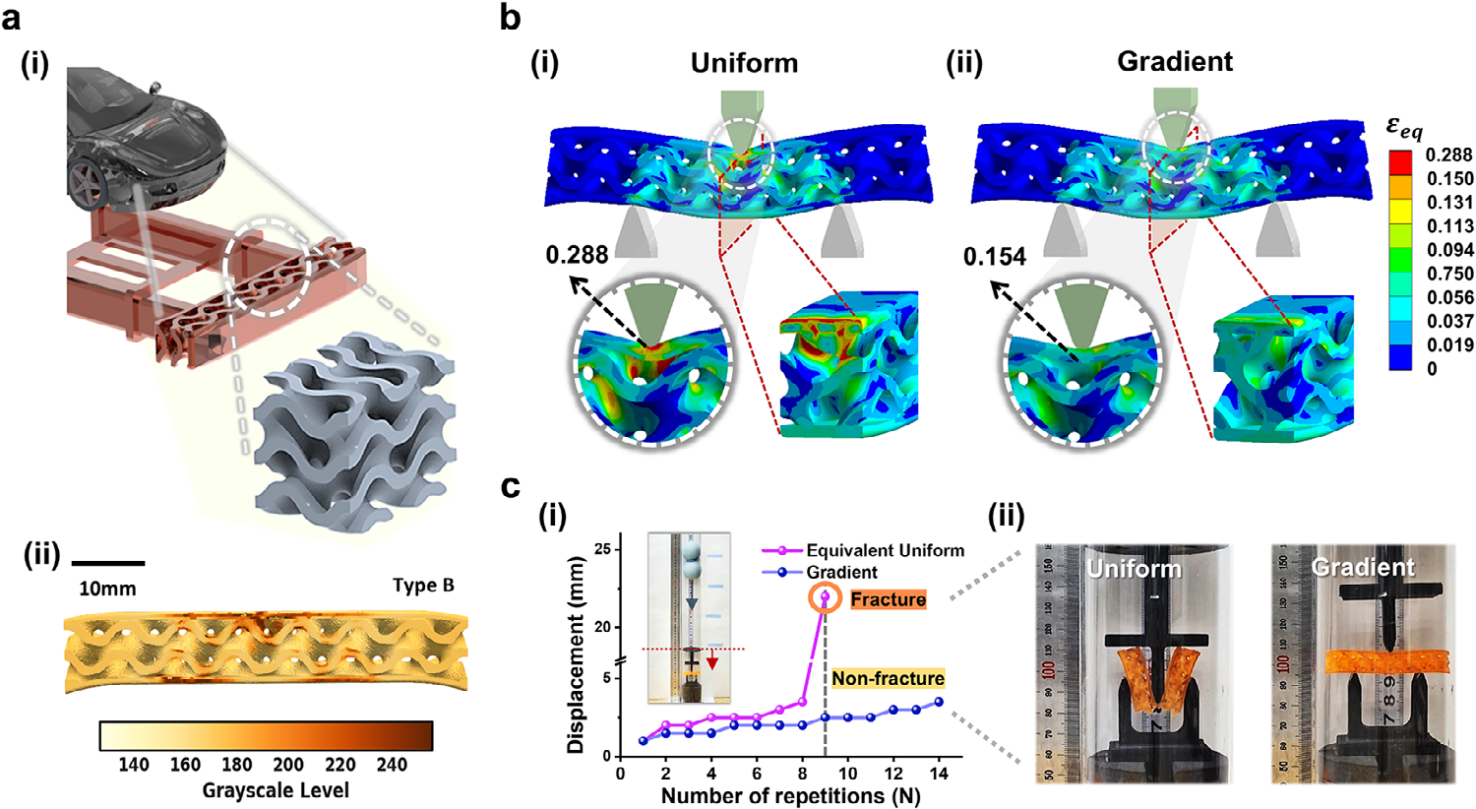
3D application of gradient design utilizing the SMBO framework with PUSA‐HUA resin: automotive energy absorbing bumper beam. a) (i) Representation of an energy‐absorbing crash beam designed for an automotive bumper, featuring a gyroid structure. The scale bar indicates 10 mm. (ii) 3D gradient structure model generated for g‐DLP 3D printing, with the color bar denoting grayscale level. b) Strain distribution plots from FEA for (i) uniform and (ii) gradient structures, with the color bar denoting equivalent strain (ɛ_eq_). c) Repeated drop test to evaluate impact resistance, comparing the optimized gradient structure and the equivalent uniform structure: (i) displacement plot of the impact jig and (ii) fracture comparison after nine impacts.

To assess the broader applicability of our framework beyond single‐axis compression to other deformation types, we investigated the effectiveness of gradient structures in automotive bumper beam designs. For automotive applications, Type B resin was selected, as its tunable modulus range of 471 to 97 MPa —achieved via g‐DLP printing—falls within that of typical impact‐absorbing plastics used in components such as bumper covers or energy‐absorbing lattice structures. Additionally, it offers sufficient damping performance (loss tangent 0.32). We utilized the gyroid structure for the bumper beam, recognized for its ability to uniformly distribute loads and enhance durability,^[^ ^48^, ^49^ ^]^ as illustrated in **Figure** **7**a‐i. Since vehicle collisions involve not only compression but also significant bending deformations, bending tests are crucial for assessing impact performance and structural stiffness. To simulate realistic conditions, we designed an optimized gradient bumper beam structure using numerical simulations based on a three‐axis bending scenario. The resulting grayscale‐based gradient structure is shown in Figure 7a‐ii.

Our results revealed that under a 3 mm upper tip displacement, the uniform structure shown in Figure 7b‐i exhibited a max(ɛ_eq_) of 0.288, while the gradient structure (Figure S33, Supporting Information) in Figure 7b‐ii demonstrated a max(ɛ_eq_) of 0.154, indicating a 47% reduction (Figure S34, Supporting Information). This signifies that our gradient design framework effectively mitigates strain concentrations from bending in automotive bumpers and confirms its applicability in complex 3D structures subjected to various loading conditions. Furthermore, to explore the impact resistance performance of the gyroid‐based bumper beam structure with gradient design (Figure S35, Supporting Information), we conducted repeated drop tests on both the optimized gradient design and the equivalent uniform cases. Figure 7c summarizes the results: (i) displacement monitoring of the impact jig, showing downward motion during impact, and (ii) fracture comparisons after nine impact cycles, with full‐cycle images provided in Figure S36 (Supporting Information). While the uniform structure fractured after nine impacts, the gradient structure withstood at least five additional impacts without failure. This improvement is attributed to effective strain concentration mitigation in critical regions enabled by the spatial modulus gradient. These findings highlight the gradient structure's enhanced durability under repeated impact loading.

## 3 Conclusion

This study presents an integrated design strategy that combines material properties with structural optimization to enhance the mechanical robustness of g‐DLP 3D‐printed structures. At the material level, the developed PUSA‐HUA resin allows for tunable mechanical properties, with an elastic modulus ranging from a few MPa to over 1 GPa, while dynamic bonding enhances viscoelastic damping across this spectrum. The resin's compatibility with g‐DLP enables spatial modulation of its properties, facilitating geometric gradients within a single structure. At the structural level, a customized ML‐driven SMBO framework optimizes gradient design by identifying configurations that minimize strain concentrations for various effective stiffness values, thereby delaying fracture initiation. This framework effectively reduces shape‐induced strain concentrations in various 2D unit cell structures, validated through both numerical analysis and experiments derived from g‐DLP. It also extends to practical 3D structures with arbitrary geometries, as demonstrated by cyclic loading tests for applications such as artificial knee cartilage in biomedical contexts. Furthermore, the framework effectively reduced strain concentration by 47% in 3D energy‐absorbing bumper beams designed with gyroids for automotive bumpers, underscoring its robustness under complex loading conditions. This was further validated through repeated drop tests, where the gradient structures delayed fracture under impact. Through this approach, the synergistic integration of PUSA‐HUA resin and the SMBO framework establishes a versatile g‐DLP 3D printing platform tailored for a wide variety of applications requiring mechanical robustness and long‐term structural stability. This platform holds significant potential for incorporating diverse structures and resin materials.

Future studies could expand their industrial applicability by exploring different functional resins for g‐DLP and optimizing gradient structures for time‐dependent loading to improve adaptive responses. In this context, we will investigate the integration of machine learning algorithms to assess the adaptability of gradient designs under time‐dependent deformation in future work.^[^ ^49^ ^]^ These efforts aim to develop a more advanced platform, broadening its applicability to metamaterials,^[^ ^41^, ^50^, ^51^, ^52^, ^53^, ^54^, ^55^ ^]^ biomimetic structures,^[^ ^56^ ^]^ electronic devices,^[^ ^23^, ^57^ ^]^ structural materials, and other advanced engineering fields.

## 4 Experimental Section

### Materials

Isophorone diisocyanate (IPDI, ≥99%) and tetrahydrofuran (THF, ≥99%) were purchased from Ducksan. 2‐Hydroxyethyl disulfide (HEDS) and hydroquinone (HQ) were obtained from Sigma‐Aldrich. 2‐Hydroxyethyl acrylate (HEA) was supplied by JUNSEI, while polytetramethylene ether glycol (PTMEG‐1000) was purchased from TCI. Diphenyl(2,4,6‐trimethylbenzoyl)phosphine oxide (TPO) was obtained from Sigma‐Aldrich. All chemicals were used as received without further purification. The detailed synthesis and composition process using the material is provided in Section S1 (Supporting Information).

### PUSA‐HUA Resin Characterizations

Fourier Transform Infrared (FTIR) spectroscopy (Nicolet iS5, Thermo Scientific) in attenuated total reflection (ATR) mode was used to analyze the chemical bonding of PUSA, HUA, and PUSA‐HUA resins, with data collected at a spectral resolution of 4 cm^−1^ and averaged over 24 scans. A confocal Raman spectrophotometer (XperRAM 200, NanoBase) with 532 nm laser excitation was employed to acquire Raman spectra.

¹H‐NMR measurements of the samples in chloroform‐d₆ were conducted using a Nuclear Magnetic Resonance (NMR) spectrometer (Unity Inova, 500 MHz) at 25 °C.

Dynamic mechanical analysis (DMA) was performed using a Hitachi DMA7100 in tensile mode following the ASTM D5026. The film specimens had dimensions of ≈30 mm × 8 mm × 0.6 mm, with an effective grip‐to‐grip length of 10 mm. For the temperature sweep, the test was conducted at a heating rate of 5 °C min^−1^ over a temperature range of −50 °C to 100 °C. The amplitude and frequency were set to 10 µm and 1 kHz, respectively. For the frequency sweep, measurements were carried out at 25 °C under stepwise frequencies of 0.1, 0.5, 1, 10, and 100 Hz, with each step maintained for 10 min.

Tensile tests were conducted at room temperature under 40% humidity using a universal testing machine (F305‐EM, Mark‐10) equipped with a 1500 N load cell at a speed of 1 mm min^−1^, in accordance with the ASTM D1708‐13 standard. For each test, more than three printed dog‐bone‐shaped specimens were used for both the PUSA‐HUA resin configuration and the g‐DLP‐printed PUSA‐HUA resin specimens.

### Finite‐Element (FE) Simulations

FE simulations were conducted to support both the optimization process and the failure behavior evaluation of the printed structures. Two different modeling approaches were adopted depending on the simulation objective. For the gradient optimization process, strain distribution analysis was performed under the small‐deformation assumption using a static linear elastic material model. The primary purpose of this step was to evaluate the effective stiffness of each design candidate and provide the necessary feedback for the optimization algorithm. Iterative evaluations during the Bayesian optimization (BO) step were conducted using a 2D unit cell model under plane stress conditions, where the element‐wise material properties were assigned according to the designed grayscale distribution.

For the failure behavior evaluation, the modeling approach was revised to account for the highly deformable, nonlinear behavior of the rubber‐like polymer material. A 2.5D unit cell‐based uniaxial tensile test was constructed in ANSYS LS‐DYNA using explicit dynamic analysis, where the material behavior was defined by the Simplified Rubber/Foam hyperelastic model. To simulate damage initiation and fracture propagation under large deformation, a strain‐based failure criterion was applied.

For the 3D application, the simulations investigated the compressive strain distribution in 3D cartilage structures and the bending‐induced strain in gyroid‐based vehicle anti‐collision beams, aiding in the evaluation of strain concentration mitigation effect across 3D multi‐deformation modes. These simulations employed a commercial static solver (ANSYS/Static, version 2022R1) with SOLID186 elements. The strain was induced in the structure by applying displacement constraints at the rigid patella and the rigid indenter. Frictional contact was established between the cartilage and the rigid support, as well as the gyroid‐based bumper beam. The outcomes of the simulations were assessed based on the deformed state and the Equivalent von Mises Strain.

### Multi‐Objective Optimization

A hybrid Bayesian optimization (HBO) was developed based on Gaussian Process Regression (GPR) to construct surrogate models. The design variables were defined as the control point coordinates (P_1_, P_2_, …, P_6_) of a Bézier curve, while the objective functions included SCF^−1^ and effective stiffness. For multi‐objective optimization, an adaptive approach combining single‐objective Bayesian optimization (SOBO) with a weighted sum strategy and multi‐objective Bayesian optimization (MOBO) using Expected Hypervolume Improvement (EHVI) was employed. In each iteration, the decision step involved two phases: 1) Weighted sum‐based EI evaluation, where five different weight combinations were used to generate ten candidate solutions covering a diverse region of the design space. 2) EHVI‐based selection, refining the Pareto front by selecting solutions that maximize hypervolume improvement among the candidates. The GPR model guided the selection of sampling points by simultaneously predicting both objectives and their associated uncertainties. The iteratively generated solutions were evaluated using FE simulations to compute the objective function values, which were subsequently used to retrain and update the GPR model at each iteration. To assess the performance of the optimization process, the distance metric (DM) was employed to evaluate the diversity of Pareto‐optimal solutions, while error analysis and cross‐validation of the GPR models ensured predictive reliability. This iterative framework facilitated efficient exploration of the design space while maintaining convergence towards an optimal trade‐off between competing objectives. This process, integrated with the FE simulation model, established a Sequential Model‐Based Bayesian Optimization (SMBO) framework, enabling iterative refinement of the surrogate model and efficient exploration of the design space.

### Digital Light Processing 3D Printing

DLP printing was performed using an IMC57 from Carima, with a 405 nm UV light source, under room temperature.

### Voxel‐Based Sliced Image Generation for g‐DLP

A voxel‐based sliced image generation method was developed for DLP 3D printing using a Python‐based pipeline. FE data from Ansys and Abaqus were processed to construct a structured voxel grid, which was then converted into an unstructured VTK format with grayscale values assigned to each voxel. For slicing, the VTK model was processed using the PyVista library, extracting parallel slices at predefined layer thickness for DLP printing. Each slice was rendered in grayscale according to voxel intensities and saved as an image, enabling precise material distribution mapping for DLP printing.

### Experimental Validation of 3D Application

FE simulation and Multi‐Objective Optimization were employed to optimize the 3D gradient structures for the artificial knee cartilage specimen. The optimized design was fabricated using g‐DLP 3D printing, which involved voxel‐based sliced image generation with the developed resin. Repeated compression tests were conducted using a universal testing machine (F305‐EM, Mark‐10). Detailed procedures are provided in Section S8 (Supporting Information). Damage evolution during cyclic loading was monitored at intervals of 200 cycles through visual inspection and image‐based analysis. At each interval, acquired images were quantitatively analyzed to extract pixel‐wise brightness levels. Regions exhibiting noticeably elevated grayscale values relative to the surrounding intact matrix were identified as potential damage zones. For clarity, these regions were visually highlighted in blue.

To evaluate the impact resistance of the gyroid‐based bumper beam structure, repeated drop weight tests were conducted using specimens fabricated by g‐DLP printing based on the 3D gradient modeling framework. A mass of ≈600 g was repeatedly dropped from a height of 25 cm to apply consistent impact loading. During the tests, displacement of the impact jig and fracture behavior of the specimens were monitored after each cycle.

## Conflict of Interest

The authors declare no conflict of interest.

## Supporting information



Supporting Information

## Data Availability

The data that supports the findings of this study are available from the corresponding author upon reasonable request.

## References

[1] L. J. Tan , W. Zhu , K. Zhou , Adv. Funct. Mater. 2020, 30, 2003062.

[2] G. Zhu , N. von Coelln , Y. Hou , C. Vazquez‐Martel , C. A. Spiegel , P. Tegeder , E. Blasco , Adv. Mater. 2024, 36, 2401561. 10.1002/adma.202401561 38949414

[3] S. Huang , H. Zhang , J. Sheng , E. Agyenim‐Boateng , C. Wang , H. Yang , J. Wei , G. Jiang , J. Zhou , J. Lu , J. Zhang , Chem. Eng. J. 2023, 465, 142830.

[4] D. Lee , D. H. Kim , H. Kim , H. M. Seung , H. C. Song , M. Kim , Compos. Part B Eng. 2024, 283, 111595.

[5] G. Zhu , Y. Hou , J. Xiang , J. Xu , N. Zhao , ACS Appl. Mater. Interfaces 2021, 13, 34954. 34270889 10.1021/acsami.1c08872

[6] D. Liu , P. Jiang , Y. Wang , Y. Lu , J. Wu , X. Xu , Z. Ji , C. Sun , X. Wang , W. Liu , Adv. Funct. Mater. 2023, 33, 2214885.

[7] K. Du , J. Basuki , V. Glattauer , C. Mesnard , A. T. Nguyen , D. L. J. Alexander , T. C. Hughes , ACS Appl. Polym. Mater. 2021, 3, 3049.

[8] C. J. Thrasher , J. J. Schwartz , A. J. Boydston , ACS Appl. Mater. Interfaces 2017, 9, 39708. 29039648 10.1021/acsami.7b13909

[9] J. Borrello , P. Nasser , J. C. Iatridis , K. D. Costa , Addit. Manuf. 2018, 23, 374. 31106119 10.1016/j.addma.2018.08.019 PMC6516765

[10] J. Nam , M. Kim , Nano Convergence 2024, 11, 45. 39497012 10.1186/s40580-024-00452-3 PMC11534933

[11] J. Xiaolin , X. Min , W. Minhui , M. Yuanhao , Z. Wencong , Z. Yanan , R. Haoxiang , L. Xun , Eur. Polym. J. 2022, 162, 110893.

[12] X. Li , R. Yu , Y. He , Y. Zhang , X. Yang , X. Zhao , W. Huang , ACS Macro Lett. 2019, 8, 1511. 35651184 10.1021/acsmacrolett.9b00766

[13] S. Peng , Y. Li , L. Wu , J. Zhong , Z. Weng , L. Zheng , Z. Yang , J. T. Miao , ACS Appl. Mater. Interfaces 2020, 12, 6479. 31927985 10.1021/acsami.9b20631

[14] H. Kim , J. Nam , Y. Il Kim , H. C. Song , J. Ryu , M. Kim , Adv. Mater. Technol. 2024, 9, 2400382.

[15] G. Dong , Y. Chang , C. Li , L. Zhao , X. Tian , X. Liu , J. Appl. Polym. Sci. 2023, 140, 54544.

[16] S. Y. Jeon , B. Shen , N. A. Traugutt , Z. Zhu , L. Fang , C. M. Yakacki , T. D. Nguyen , S. H. Kang , Adv. Mater. 2022, 34, 2022272. 10.1002/adma.202200272 35128733

[17] B. Sun , G. Kitchen , D. He , D. K. Malu , J. Ding , Y. Huang , A. Eisape , M. M. Omar , Y. Hu , S. H. Kang , Sci. Adv. 2025, 11, eadt3979. 39919188 10.1126/sciadv.adt3979 PMC11804925

[18] G. Zhu , H. A. Houck , C. A. Spiegel , C. Selhuber‐Unkel , Y. Hou , E. Blasco , Adv. Funct. Mater. 2024, 34, 2300456.

[19] M. Caprioli , I. Roppolo , A. Chiappone , L. Larush , C. F. Pirri , S. Magdassi , Nat. Commun. 2021, 12, 2462. 33911075 10.1038/s41467-021-22802-z PMC8080574

[20] L. Yue , S. M Montgomery , X. Sun , L. Yu , Y. Song , T. Nomura , M. Tanaka , H. J Qi , Nat. Commun. 2023, 14, 1251. 36878943 10.1038/s41467-023-36909-y PMC9988868

[21] X. Kuang , J. Wu , K. Chen , Z. Zhao , Z. Ding , F. Hu , D. Fang , H. J Qi , Sci. Adv. 2019, 5, aav5790. 10.1126/sciadv.aav5790 PMC6499595 31058222

[22] B. Zhao , M. Zhang , L. Dong , D. Wang , Compos. Commun. 2022, 36, 101395.

[23] F. Gholami , L. Yue , M. Li , A. Jain , A. Mahmood , M. Fratarcangeli , R. Ramprasad , H. J. Qi , Adv. Mater. 2024, 36, 2408774. 10.1002/adma.202408774 39340273

[24] C. T. Forte , S. M. Montgomery , L. Yue , C. M. Hamel , H. J. Qi , J. Appl. Mech. Trans. ASME 2023, 90, 071003.

[25] S. M. Montgomery , C. M. Hamel , J. Skovran , H. J. Qi , Extreme Mech. Lett. 2022, 53, 101714.

[26] M. Zhang , X. Fan , L. Dong , C. Jiang , O. Weeger , K. Zhou , D. Wang , Adv. Sci. 2024, 11, 2309932. 10.1002/advs.202309932 PMC11267290 38769665

[27] N. D. Dolinski , E. B. Callaway , C. S. Sample , L. F. Gockowski , R. Chavez , Z. A. Page , F. Eisenreich , S. Hecht , M. T. Valentine , F. W. Zok , C. J. Hawker , ACS Appl. Mater. Interfaces 2021, 13, 22065. 33929835 10.1021/acsami.1c06062

[28] E. Rossegger , J. Strasser , R. Höller , M. Fleisch , M. Berer , S. Schlögl , Macromol. Rapid Commun. 2023, 44, 2200586. 10.1002/marc.202200586 36107158

[29] L. Yue , X. Sun , L. Yu , M. Li , S. M. Montgomery , Y. Song , T. Nomura , M. Tanaka , H. J. Qi , Nat. Commun. 2023, 14, 5519. 37684245 10.1038/s41467-023-41170-4 PMC10491591

[30] X. Peng , L. Yue , S. Liang , S. Montgomery , C. Lu , C. M. Cheng , R. Beyah , R. R. Zhao , H. J. Qi , Adv. Funct. Mater. 2022, 32, 2112329.

[31] Y. Zhou , Q. Lin , J. Hong , N. Yang , Structures 2021, 29, 561.

[32] Z. Yang , C. B. Kim , C. Cho , H. G. Beom , Int. J. Solids Struct. 2008, 45, 713.

[33] Y. Li , Z. Feng , L. Hao , L. Huang , C. Xin , Y. Wang , E. Bilotti , K. Essa , H. Zhang , Z. Li , F. Yan , T. Peijs , Adv. Mater. Technol. 2020, 5, 1900981.

[34] K. Park , C. Song , J. Park , S. Ryu , M. Horiz. 2023, 10, 4329. 10.1039/d3mh00137g 37434475

[35] Z. Yang , K. Park , J. Nam , J. Cho , Y. J. Choi , Y. Il Kim , H. Kim , S. Ryu , M. Kim , Adv. Sci. 2024, 11, 2402440. 10.1002/advs.202402440 PMC11434127 38935025

[36] B. Peng , Y. Wei , Y. Qin , J. Dai , Y. Li , A. Liu , Y. Tian , L. Han , Y. Zheng , P. Wen , Nat. Commun. 2023, 14, 6630. 37857648 10.1038/s41467-023-42415-y PMC10587057

[37] P. Serles , J. Yeo , M. Haché , P. G. Demingos , J. Kong , P. Kiefer , S. Dhulipala , B. Kumral , K. Jia , S. Yang , T. Feng , C. Jia , P. M. Ajayan , C. M. Portela , M. Wegener , J. Howe , C. V. Singh , Y. Zou , S. Ryu , T. Filleter , Adv. Mater. 2025, 37, 2410651. 39846271 10.1002/adma.202410651 PMC11983246

[38] P.‐F. Cao , M. D. Bartlett , Z. Cui , S. H. Pun , C. Yu , Adv. Funct. Mater. 2018, 28, 1800741.

[39] S. M. Montgomery , F. Demoly , K. Zhou , H. J. Qi , Adv. Funct. Mater. 2023, 33, 2213252.

[40] J. E. Hergert , A. C. Uzcategui , A. Muralidharan , V. Crespo‐Cuevas , V. L. Ferguson , R. R. McLeod , Adv. Eng. Mater. 2022, 24, 2101543.

[41] S. Lee , W. Choi , J. W. Park , D. S. Kim , S. Nahm , W. Jeon , G. X. Gu , M. Kim , S. Ryu , Nano Energy 2022, 103, 107846.

[42] W. C. Young , R. G. Budynas , Roark's Formulas for Stress and Strain, 7th ed., McGraw‐Hill, New York, NY, USA 2002.

[43] I. Y. Kim , O. L. De Weck , Struct. Multidiscip. Optim. 2006, 31, 105.

[44] Y. Collette , P. Siarry , Multiobjective Optimization: Principles and Case Studies, 1st ed., Springer, Berlin, Heidelberg, Germany 2003.

[45] W. Weibull , J. Appl. Mech. 1951, 18, 293.

[46] N. A. Traugutt , D. Mistry , C. Luo , K. Yu , Q. Ge , C. M. Yakacki , Adv. Mater. 2020, 32, 2000797. 10.1002/adma.202000797 32508011

[47] A. K. Means , C. S. Shrode , L. V. Whitney , D. A. Ehrhardt , M. A. Grunlan , Biomacromol. 2019, 20, 2034. 10.1021/acs.biomac.9b00237 31009565

[48] Z. Gan , M. D. Turner , M. Gu , Sci. Adv. 2016, 2, 1600084. 10.1126/sciadv.1600084 PMC4928936 27386542

[49] L. Han , S. Che , Adv. Mater. 2018, 30, 1705708. 10.1002/adma.201705708 29543352

[50] P. Thakolkaran , M. A. Espinal , S. Dhulipala , S. Kumar , C. M. Portela , Extreme Mech. Lett. 2025, 74, 102274.

[51] D. H. Kim , H. H. Kwon , W. H. Cho , M. Kim , Thin‐Walled Struct. 2025, 210, 112943.

[52] H. Kwon , D. H. Kim , W. H. Cho , M. Kim , Adv. Mater. Technol. 2025, 10, 2401910.

[53] T. Y. Kim , S. H. Park , K. Park , Addit. Manuf. 2021, 47, 102254.

[54] D. S. Kim , W. Choi , S. W. Kim , E. J. Kim , S. Nahm , M. Kim , Mater. Horiz 2022, 10, 149. 10.1039/d2mh01041k 36321368

[55] A. Farzaneh , N. Pawar , C. M. Portela , J. B. Hopkins , Nat. Commun. 2022, 13, 28696. 10.1038/s41467-022-28696-9 PMC8873317 35210416

[56] Y. Yang , X. Song , X. Li , Z. Chen , C. Zhou , Q. Zhou , Y. Chen , Adv. Mater. 2018, 30, 1706539. 10.1002/adma.201706539 29920790

[57] Q. Mu , L. Wang , C. K. Dunn , X. Kuang , F. Duan , Z. Zhang , H. J. Qi , T. Wang , Addit. Manuf. 2017, 18, 74.

