# Supplementary material for: Machine Learning‐Driven Grayscale Digital Light Processing for Mechanically Robust 3D‐Printed Gradient Materials
Source: Adv Mater. 2025 Jul 16;37(42):2504075. doi: 10.1002/adma.202504075 (PMC12548508; doi:10.1002/adma.202504075)
Supplement: Supplementary file 1 — Supporting Information [file ADMA-37-2504075-s001.docx]

*Supporting Information*

Machine Learning-Driven Grayscale Digital Light Processing for Mechanically Robust 3D-Printed Gradient Materials

Jisoo Nam, Boxin Chen, and Miso Kim*

J. Nam, B. Chen, M. Kim

Department of Mechanical Engineering, Korea Advanced Institute of Science and Technology (KAIST), Daejeon, 34141 Republic of Korea

*E-mail: misokim@kaist.ac.kr (M. Kim)

B. Chen

School of Advanced Materials Science and Engineering, Sungkyunkwan University (SKKU), Suwon, 16419 Republic of Korea

Keywords: 3D printing, grayscale digital light processing, polyurethane acrylate, dynamic bond, gradient structure, machine learning, multi-objective optimization

**Table of Contents**

S1. Synthesis and Characterization of PUSA and HUA6

S2. Study on the Viscoelastic Characteristics of PUSA based on HEDS Compositions10

S3. PUSA-HUA Resin Formulation and Characterization for DLP 3D Printing 12

S4. PUSA-HUA Resin Application for g-DLP Printing22

S5. Gradient Structure Design and Optimization Strategies32

S6. Application of Gradient Structure Design and Optimization for 2D Unit Cell Structures46

S7. Validation of 2D Unit Cell Structures52

S8. 3D Application Case I: Artificial Human Knee Cartilage58

S9. 3D Application Case II: Energy Absorption Beam in Automotive Bumpers63

**S1. Synthesis and Characterization of PUSA and HUA**

**One-pot Synthesis of Polyurethane Acrylate containing Disulfide Bonds (PUSA)**
Polyurethane acrylate containing disulfide bonds (PUSA) was synthesized via a stepwise addition polymerization process. As mentioned in the main text, *x* in PUSA(*x*) represents the molar ratio of HEDS to PTMEG. Here, the synthesis procedure is described using PUSA(1) as a representative example. The reaction was carried out using a three-necked round-bottom flask equipped with a mechanical stirrer, a dropping funnel, and a thermometer. The detailed synthesis procedure is described below, taking the molar ratio of PTMEG: HEDS = 1:2 as an example. Initially, Isophorone diisocyanate (IPDI, 13.33 g, 60.00 mmol) and Dibutyltin dilaurate (DBTDL, 0.0133 g, 0.1 wt% of IPDI) were heated to 60 °C under nitrogen protection. Polytetramethylene ether glycol (PTMEG-1000, 15 g, 15.00 mmol) was added dropwise to the flask, and the mixture was stirred for three hours to form the polyurethane prepolymer. Then, tetrahydrofuran (THF, 25 mL) was added, followed by the dropwise addition of 2-hydroxyethyl disulfide (HEDS, 3.483 g, 30.00 mmol). In the final step, 2-hydroxyethyl acrylate (HEA, 3.484 g, 30.00 mmol) and hydroquinone (HQ, 0.004 g) were introduced into the reaction at 55 °C. The reaction was monitored using Fourier transform infrared (FTIR) spectroscopy to confirm the progress of the reaction, particularly the consumption of isocyanate groups (-NCO).^[1]^ The resulting polyurethane acrylate (PUSA(1)) was dried under vacuum to remove residual solvents.

**Synthesis of HEA-based Aliphatic Urethane Acrylate (HUA)**

In a one-step synthesis, IPDI (5.55 g, 25.00 mmol) and the catalyst DBTDL (0.0056 g, 0.1 wt% % of IPDI) were added to a three-necked round-bottom flask equipped with a mechanical stirrer, a dropping funnel, a thermometer, and a condenser, and the mixture was heated to 55 °C. Then, HEA (7.55 g, 65.00 mmol) was added dropwise into the flask under a nitrogen atmosphere. The reaction mixture was stirred for three hours at 55 °C until the absorption of isocyanate groups (-NCO) disappeared on the FTIR spectrum. Finally, the HUA was obtained and subsequently dried under vacuum to remove residual solvent.

**FTIR Analysis of PUSA and HUA**

The FTIR spectra in Figure S1a illustrate the chemical transformations during the synthesis of PUSA(1). The -NCO stretching peak at 2260 cm^-1^ in the prepolymer spectrum confirms the presence of isocyanate groups, which disappear after chain extension, indicating complete consumption. The formation of urethane bonds is further evidenced by peaks at 1720 cm^-1^ (-C=O stretching), 1532 cm^-1^ (-NH bending), 2950 cm^-1^ and 2862 cm^-1^ (C-H stretching), and 1192 cm^-1^ (C-O-C stretching).

FTIR spectra of the synthesized HUA, shown in Figure S1b, exhibit similar urethane-related peaks with variations in intensity. The N-H stretching appears at about 3320 cm^-1^ in both. The asymmetric C-H stretching at 2946 cm^-1^ is observed in both, while the symmetric C-H stretching at 2862 cm^-1^ is more pronounced in PUSA(1) due to its more extended polyol backbone. In contrast, with its shorter chain and higher acrylate density, HUA shows a weaker symmetric -CH₂ stretching while maintaining a strong asymmetric stretching signal.


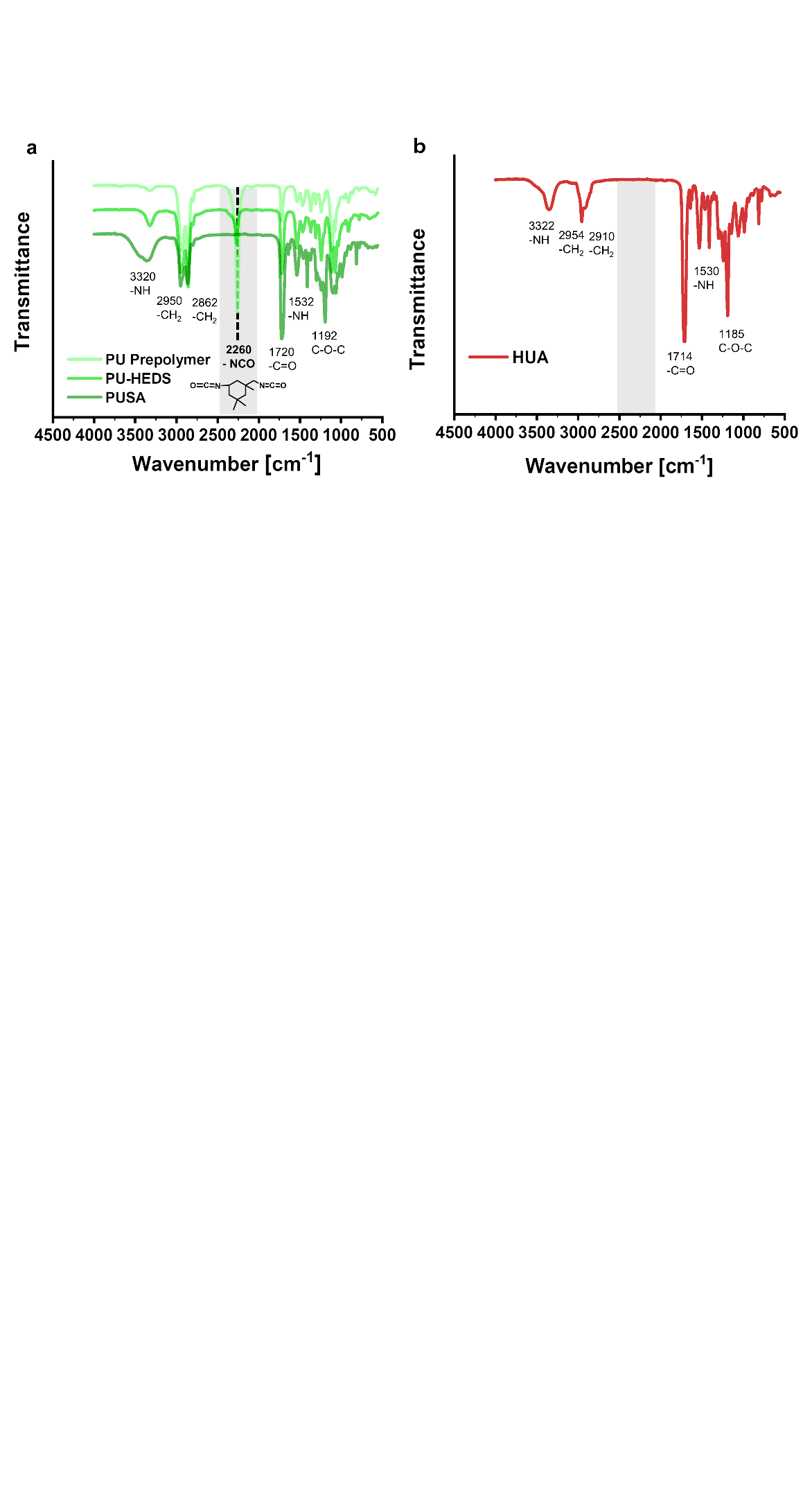


**Figure S1.** FTIR spectra of synthesized polymers. (a) Prepolymer, PU-HEDS, and finalized PUSA(1). (b) Finalized HUA.

**Proton Nuclear Magnetic Resonance (^1^H-NMR) Spectrum Analysis confirming PUSA**

The ^1^H-NMR spectrum of the PUSA, using PUSA(1) as a representative example, reveals characteristic chemical shifts (δ) corresponding to its structural components (Figure S2). Vinyl protons from the acrylate terminal groups appear at 5.5-6.5 ppm, confirming the presence of reactive double bonds. Methylenes adjacent to ester oxygen (-CH_2_-O-C=O) are observed at 4.0-4.5 ppm, while disulfide-adjacent methylenes (-CH_2_-S-S) and urethane-linked groups (-CH_2_-NH-C(O)-) overlap in the 3.5-4.2 ppm range. PTMEG backbone methylenes, shielded by the aliphatic environment, are found near 2.0 ppm, with additional aliphatic protons appearing in the 1.5–2.0 ppm region. Methyl protons derived from isocyanate groups are most upfield shift at 1.5 ppm, reflecting their highly shielded environment. The spectrum highlights the distinct chemical environments introduced by acrylate terminals, urethane linkages, polyols, and HEDS segments, with some overlapping signals due to polymer chain dynamics.


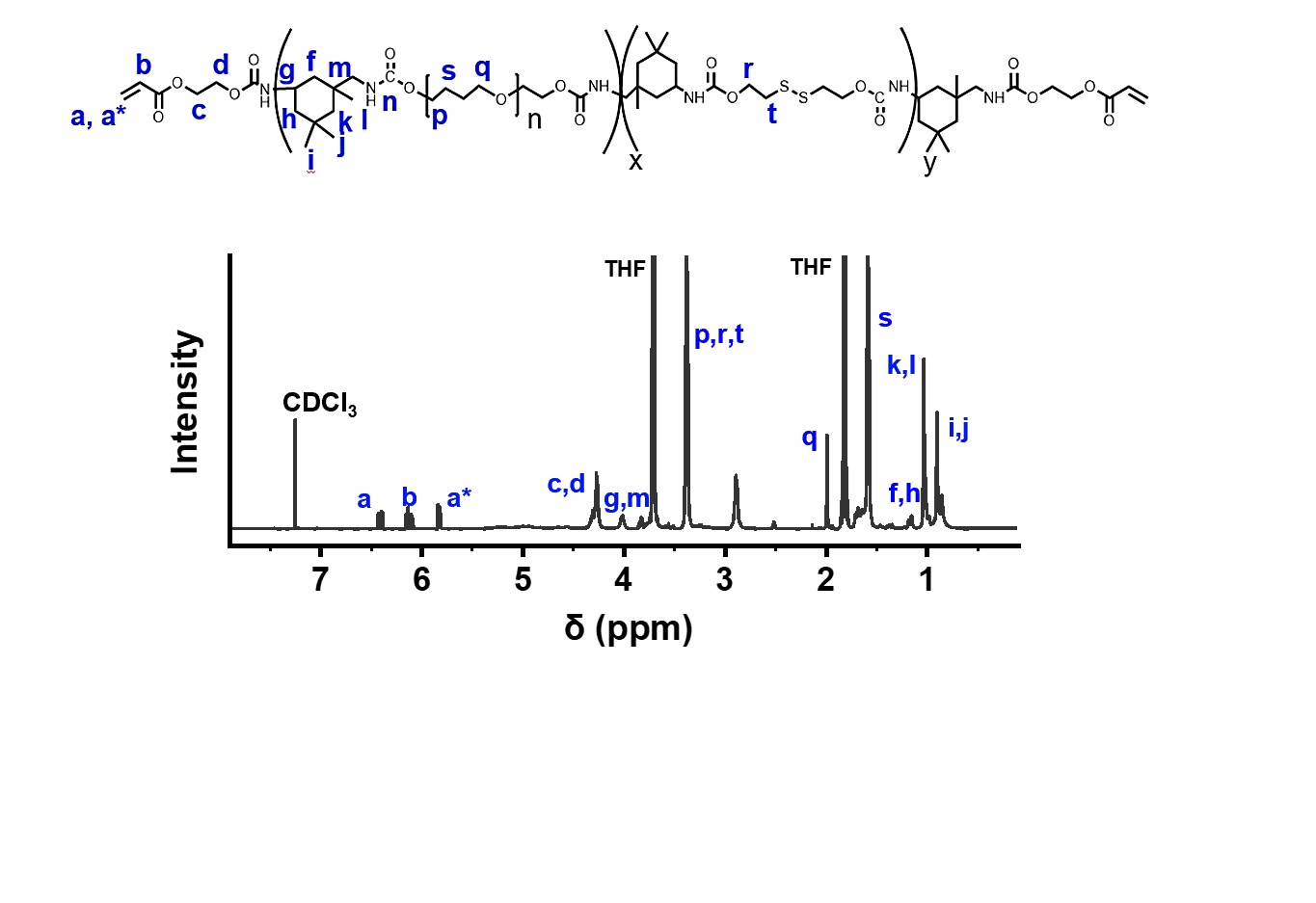


**Figure S2.** ^1^H-NMR spectrum of PUSA(1).

The ^1^H-NMR spectrum of the synthesized HUA in Figure S3 highlights key chemical shifts (δ) corresponding to its structure. Similar to the PUSA(1) spectrum, vinyl protons from the acrylate terminal groups are observed at 5.0-6.5 ppm. Methylenes adjacent to ester oxygen (-CH-O-C=O) appear in the 4.0–4.5 ppm range, with additional contributions from urethane-linked methylenes (-CH-NH-C(O)-) near 3.7–4.0 ppm. Aliphatic methylenes in the backbone are shielded and found near 1.5–2.0 ppm, while terminal methyl groups (-CH) resonate at 1.0 ppm. Unlike the PUSA spectrum, the absence of disulfide linkages and PTMEG segments simplifies the spectrum.


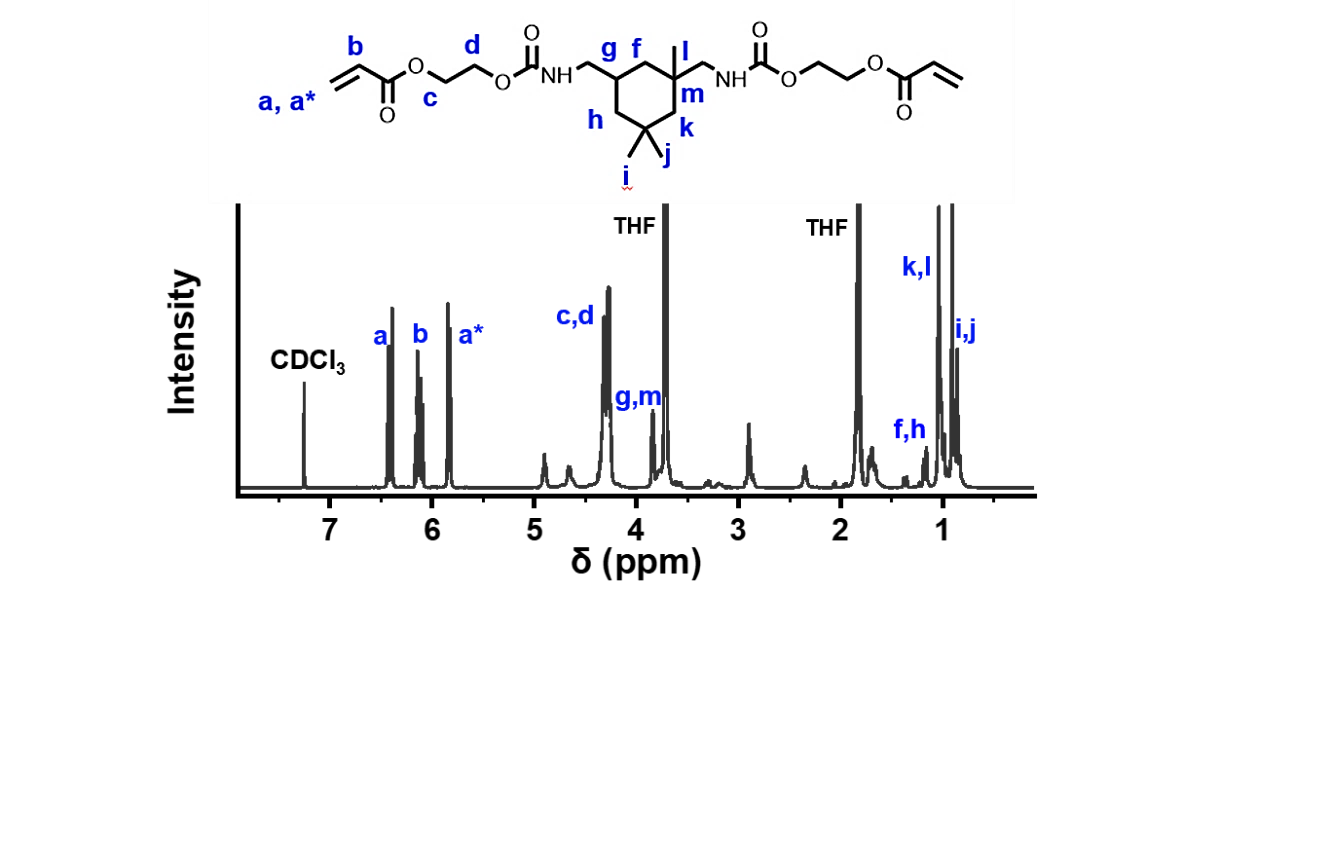


**Figure S3.** ^1^H-NMR spectrum of HUA.

**S2. Study on the Viscoelastic Characteristics of PUSA based on HEDS Compositions**

**Dynamic Mechanical Analysis of PUSA with Varying Disulfide Bond Content**

In this study, PUSA(x) formulations with varying disulfide bond contents were synthesized and characterized to evaluate their viscoelastic and mechanical properties. The baseline formulation, referred to as PUSA(1), was synthesized following the detailed procedure described earlier. Additional formulations, PUSA(0.5) and PUSA(1.5), were prepared by varying the relative molar amounts of 2-hydroxyethyl disulfide (HEDS) per mole of isocyanate (-NCO) functional groups. The molar ratios of reactants for all formulations are summarized in Table S1, which presents data where the -NCO to -OH bond amount ratio is consistently maintained at 1:1.

**Table S1.** Compositions of PUSA(x) with varying disulfide bond contents.

|  | PTMEG | HEDS | HEA | IPDI |
| --- | --- | --- | --- | --- |
| PUSA(0.5) | 15 | 15 | 30 | 45 |
| PUSA(1) | 15 | 30 | 30 | 60 |
| PUSA(1.5) | 15 | 45 | 30 | 75 |

Dynamic mechanical analysis (DMA) was performed to evaluate the viscoelastic properties of PUSA formulations with varying disulfide bond contents, as presented in **Figure S4**a and S4b. As the HEDS content increases, both the storage and loss moduli rise, with PUSA(1.5)—which has the highest disulfide bond concentration—demonstrating the most significant improvement in stiffness and energy dissipation at room temperature. This enhancement is linked to the presence of more extended molecular chains. The tan δ curves further illustrate the energy dissipation effect. In PUSA(1.5), disulfide bonds within the extended molecular chains enhance energy dissipation, while urethane chains maintain structural rigidity, keeping the glass transition temperature (T_g_) nearly unchanged. Furthermore, the effect of HEDS content on stress relaxation was evaluated, as shown in Figure S4c. PUSA(0.5) exhibited the longest relaxation time due to its lower disulfide bond content and shorter molecular chains, which restricted mobility. In contrast, PUSA(1) and PUSA(1.5), with higher disulfide contents and longer chains, exhibited shorter relaxation times, which facilitated stress redistribution. In addition to these thermomechanical properties, the viscosities of the formulations, diluted with 50 wt% HEA, were measured and compared; the results are summarized in Figure S4d. The selected PUSA(1) formulation maintains an appropriate viscosity of 1980, which is below 3000, while exhibiting excellent dissipation characteristics.

**
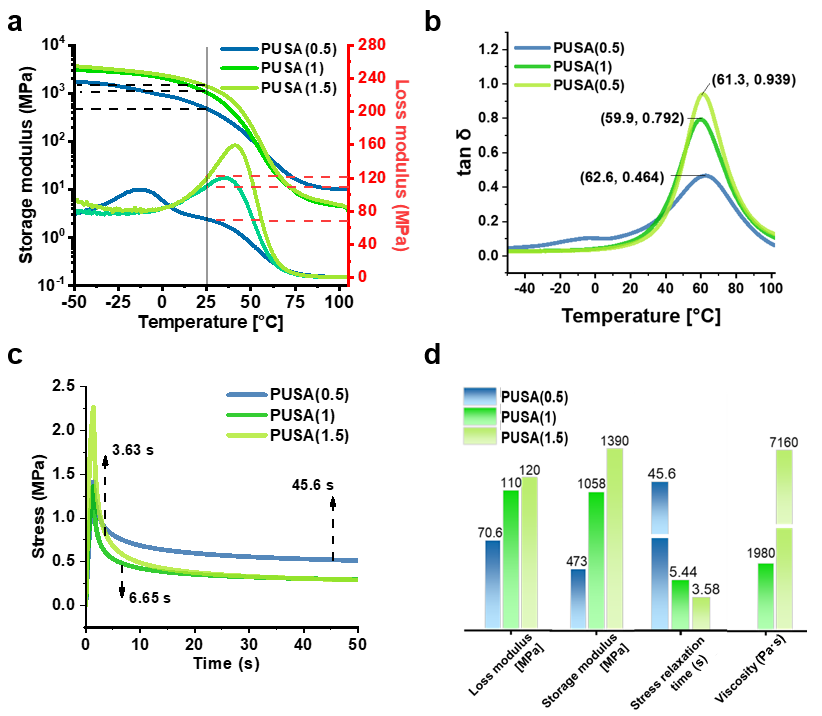
**

**Figure S4.** Dynamic mechanical analysis results for PUSA(x), including (a) storage modulus and loss modulus curves, (b) tan δ as a function of temperature, (c) stress relaxation curves, and (d) comparison of storage modulus, loss modulus, stress relaxation time, and viscosity for PUSA(x) with x = 0.5, 1, and 1.5.

**Comparison of HEDS and BDO as Chain Extenders**

**Table S2** summarizes the compositions of PUSA(1) and PUBA(1). The synthesis process for PUBA(1) follows the same stepwise procedure as PUSA(1), with 1,4-butanediol (BDO) used as the chain extender instead of 2-hydroxyethyl disulfide (HEDS). Specifically, after the formation of the polyurethane prepolymer, 1,4-butanediol (BDO, 2.705 g, 30.00 mmol) is added dropwise to replace HEDS in the second step of the reaction. The reaction temperature and time remain identical to those used for PUSA(1), ensuring consistent conditions for the formation of the polyurethane acrylate. The final product, PUBA(1), is dried under vacuum alongside PUSA(1) to remove any residual solvents.

BDO is a linear diol commonly used as a chain extender in the synthesis of polyurethanes. As a representative chain extender without dynamic bonding, BDO was selected to evaluate the effect of disulfide bonds on viscoelastic performance. DMA results reveal that PUSA(1) exhibits a higher tan δ peak (0.792) at its glass transition temperature (T_g_) (Figure S5a) and a greater loss modulus (109.8 MPa) at room temperature (RT, 25 °C) (Figure S5b). These findings indicate that the reversible nature of disulfide bonds contributes to enhanced energy dissipation and viscoelastic performance.^[2]^ This suggests that, in addition to the viscoelastic effects induced by the increased chain length from HEDS as a chain extender, the dynamic disulfide bonds also play a significant role in improving the overall viscoelastic properties.

**Table S2.** Compositions of PUSA(1) and PUBA(1).

|  | PTMEG  [mmol] | HEDS/BDO  [mmol] | HEA  [mmol] | IPDI  [mmol] |
| --- | --- | --- | --- | --- |
| PUSA(1) | 15 | 30 | 30 | 45 |
| PUBA(1) | 15 | 30 | 30 | 60 |


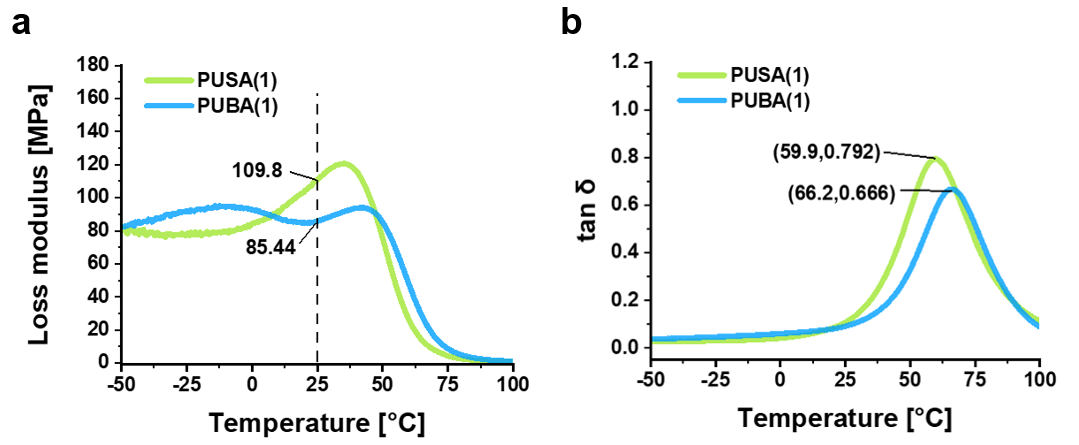


**Figure S5.** Dynamic viscoelastic properties of PUSA(1) and PUBA(1). (a) tan δ as a function of temperature and (b) loss modulus curves, demonstrating the influence of disulfide bonds on damping behavior and energy dissipation.

**S3. PUSA-HUA Resin Formulation and Characterization for DLP 3D Printing Resin Formulations for DLP 3D Printing**

The synthesized PUSA(1) is viscous, posing challenges for DLP 3D printing. To reduce viscosity, 2-hydroxyethyl acrylate (HEA)—a photocurable monomer and end-capping agent used in PUSA(1) synthesis—was added as a reactive diluent (50 wt% relative to PUSA(1)). For photopolymerization, diphenyl(2,4,6-trimethylbenzoyl)phosphine oxide (TPO, 2 wt%) was included to ensure proper curing and printability. The final resin composition is summarized in **Table S4**.

The addition of HEA resulted in a decrease in the elastic modulus of PUSA resin. DMA (**Figure S6**) further shows that the storage modulus (E′) dropped from 1058 MPa to 499 MPa, while the loss modulus (E″) increased from 110 MPa to 152 MPa upon HEA incorporation. The corresponding values are summarized in **Table S3**. The elastic modulus reported in **Table S3** reflects the initial stiffness, calculated as the average slope of the stress-strain curve within the 0–0.02 strain range. These changes indicate reduced stiffness and enhanced energy dissipation due to the presence of HEA. Its hydroxyl groups (–OH) can form additional hydrogen bonds between polymer chains, which, along with the original disulfide bonds in the PUSA network, create a composite dynamic bonding network.


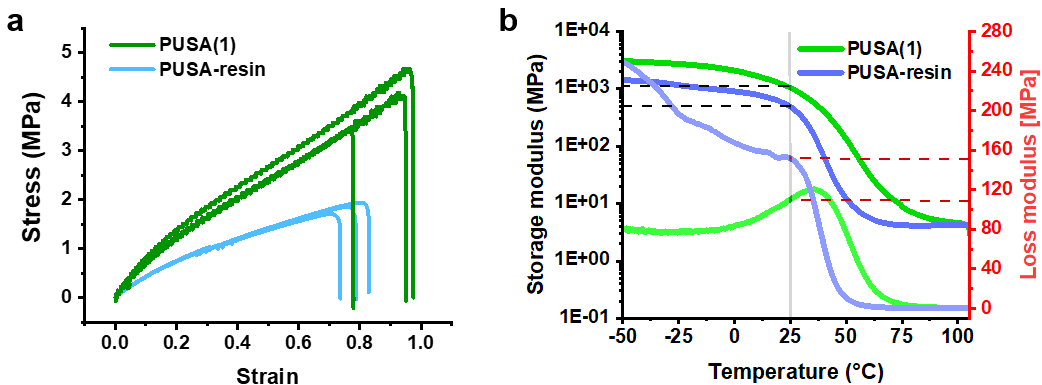


**Figure S6.** Comparison of elastic and viscoelastic properties between PUSA(1) and its resin formulation. (a) Uniaxial tensile stress–strain curves of PUSA(1) and PUSA resin. (b) Temperature-dependent storage and loss modulus measured by DMA.

**Table S3.** Elastic and viscoelastic properties of PUSA(1) and its resin formulation.

|  | Elastic modulus  (MPa) | Storage modulus  at 25°C (MPa) | Loss modulus  at 25°C (MPa) |
| --- | --- | --- | --- |
| PUSA(1) | 9.429 ± 1.141 | 1058 | 110 |
| PUSA-resin | 5.516 ± 0.196 | 499 | 152 |

**Table S4**. Compositions of the photocurable PUSA-resin.

|  | PUSA(1)  [wt%] | Reactive Diluent  (HEA) [wt%] | TPO  [wt%] |
| --- | --- | --- | --- |
| PUSA-resin | 65.36 | 32.68 | 1.96 |

No additional diluent was required for HUA, and only TPO (2 wt%) was added. The final formulation is detailed in **Table S5**.

**Table S5.** Compositions of the photocurable HUA-resin.

|  | HUA [wt%] | TPO [wt%] |
| --- | --- | --- |
| HUA-resin | 98.04 | 1.96 |

**Tailoring Mechanical Properties by Adjusting PUSA-resin: HUA-resin Ratios**

The mechanical properties of UV-cured PUSA-HUA resins were evaluated by varying the PUSA-resin: HUA-resin ratio (P:H) to identify optimal compositions for DLP 3D printing. Based on the results from Figure 2b, the mechanical properties and detailed composition data for the six selected formulations are summarized in Table S6.

**Table S6.** Compositions and mechanical properties of UV-cured PUSA-HUA resins. (P:H = weight ratio of PUSA-resin to HUA-resin)

| Composition | Elastic modulus  [MPa] | Failure Strain  [mm mm^-1^] | Tensile strength  [MPa] | Toughness  [MJ m^-3^] |
| --- | --- | --- | --- | --- |
| P:H = 9:1 | 64.6 ± 9.4 | 0.717 ± 0.029 | 7.42 ± 1.06 | 3.77 ± 0.61 |
| P:H = 8:2 | 307 ± 28.8 | 0.421 ± 0.046 | 16.1 ± 0.54 | 5.45 ± 0.32 |
| P:H = 7:3 | 593 ± 13.5 | 0.227 ± 0.026 | 24.1 ± 0.65 | 4.63 ± 0.62 |
| P:H = 6:4 | 895 ± 30.6 | 0.166 ± 0.016 | 32.8 ± 1.08 | 4.54 ± 0.61 |
| P:H = 5:5 | 1160 ± 65.3 | 0.086 ± 0.004 | 43.4 ± 1.08 | 2.69 ± 0.24 |
| P:H = 4:6 | 1490 ± 43.5 | 0.069 ± 0.001 | 56.0 ± 0.26 | 2.58 ± 0.07 |

**Mechanical Characterization of DLP 3D-Printed PUSA-HUA 3-Type Resins**

To examine the mechanical properties of selected PUSA-HUA resins that were UV-cured through DLP 3D printing, tensile tests were conducted following the ASTM D1708-13 guidelines. Dogbone specimens were fabricated by curing each layer (100 µm thickness) with an energy dose of 14.35 mJ cm^-2^. The stress-strain behavior of the selected compositions is presented in Figure 2e. **Table S7** summarizes the mechanical properties of the three PUSA-HUA resin types. The elastic modulus (E) for each composition was determined from the initial elastic region of the stress-strain curves, calculated as the average slope in the strain range of 0 to 0.02.

**Table S7.** Mechanical properties of 3 types DLP 3D-printed resins

| Grayscale | Elastic modulus  [MPa] | Failure Strain  [mm mm^-1^] | | Tensile strength  [MPa] | | Toughness  [MJ m^-3^] |
| --- | --- | --- | --- | --- | --- | --- |
| Type A | 8.29 ± 0.55 | | 0.810 ± 0.033 | | 2.87 ± 0.04 | 1.61 ± 0.08 |
| Type B | 472 ± 67.91 | | 0.478 ± 0.072 | | 21.6 ± 2.48 | 8.13 ± 1.63 |
| Type C | 1230 ± 43.51 | | 0.182 ± 0.001 | | 40.0 ± 1.26 | 3.57 ± 0.07 |

**Viscoelastic Damping Characteristics of DLP 3D-Printed PUSA-HUA Resins**

**Figure S7** presents the full DMA frequency sweep data at room temperature for the PUSA-HUA resins in the range of 0.1–100 Hz. Figure S7a shows the measured storage modulus, S7b shows the loss modulus, and S7c shows the calculated tan δ across 5 level frequencies: 0.1, 0.5, 1, 10, and 100 Hz. When comparing the three types of resins, Type A exhibited a pronounced increase in both storage and loss modulus within the 0.1–100 Hz frequency range, indicating enhanced molecular mobility. Type B showed a slight change, while Type C displayed near-saturation behavior in this frequency range, confirming highly restricted molecular motion characteristic of a stable glassy state.

Figure S7d, S7e, and S7f show the time evolution of (i) storage modulus, (ii) loss modulus, and (iii) tan δ, respectively, measured at five fixed frequencies (0.1, 0.5, 1, 10, and 100 Hz) for each resin type (A, B, and C). Each frequency was held for 10 minutes to monitor the stability of the viscoelastic response over time. Initial slight fluctuations due to instrument and sample stabilization are observed, after which the properties stabilize, allowing reliable determination of the viscoelastic behavior for each resin type at different frequencies.


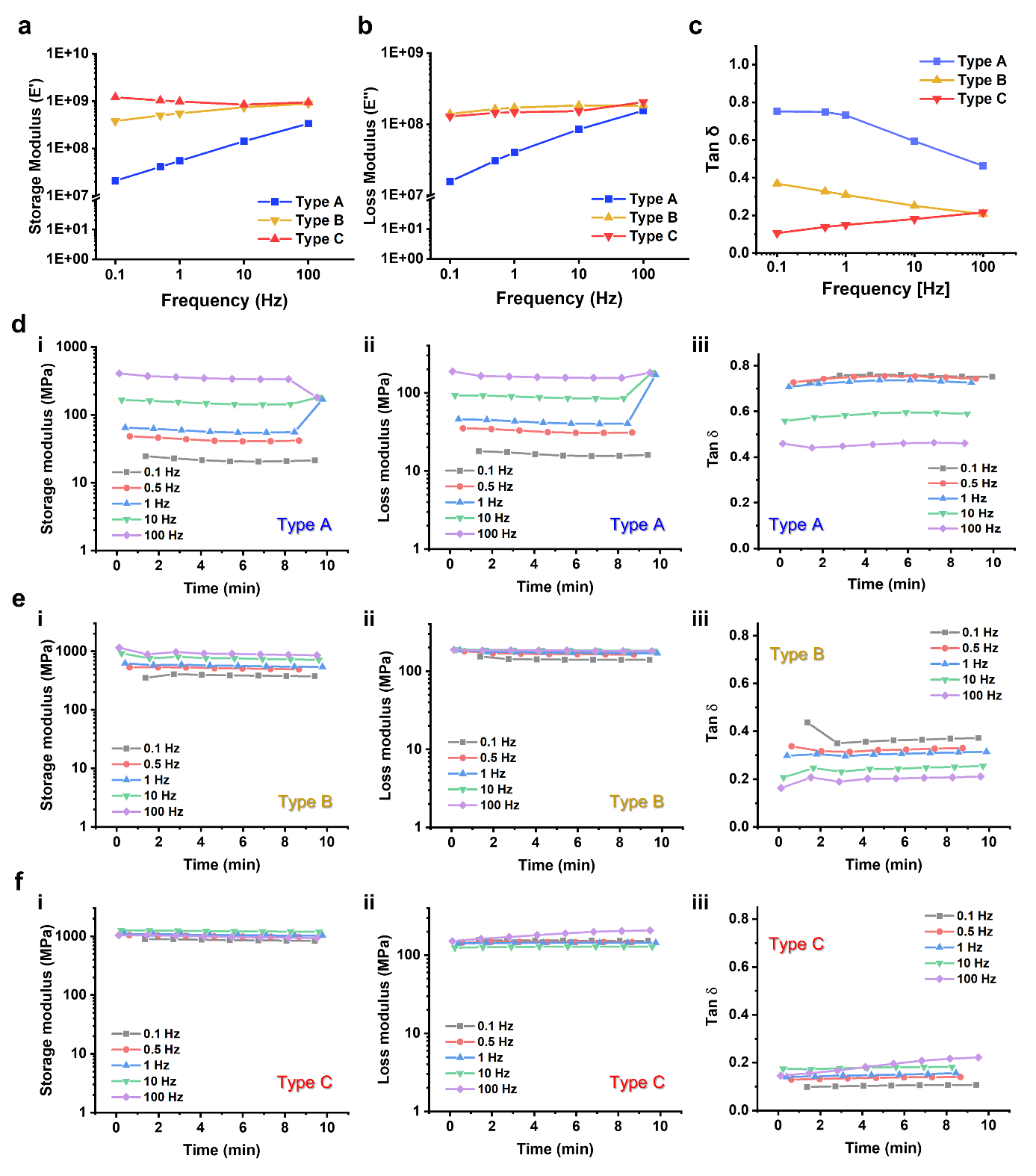


**Figure S7.** Frequency-dependent viscoelastic behavior of PUSA-HUA resin compositions obtained from DMA frequency sweep tests at five fixed frequencies (0.1, 0.5, 1, 10, and 100 Hz) conducted at room temperature. (a) Storage modulus, (b) loss modulus, and (c) tan δ. Time evolution of viscoelastic properties at five fixed frequencies (0.1, 0.5, 1, 10, and 100 Hz) for Types (d) A, (e) B, and (f) C resins are presented, showing the (i) storage modulus, (ii) loss modulus, and (iii) tan δ for each type.

When the properties of the PUSA-HUA resin developed in this study are plotted on the Ashby chart (Young’s modulus versus loss coefficient, Figure S8), the Type C resin is marked with a red star, Type B with a yellow star, and Type A with a blue star. Among these, Type A is positioned within the upper range of its modulus category, indicating superior performance. Meanwhile, Types B and C, which have higher Young’s moduli, demonstrate damping characteristics that exceed those of many conventional materials. These findings suggest that the applicability of photocurable acrylate-based materials—commonly viewed as limited in damping capability—can be significantly expanded.


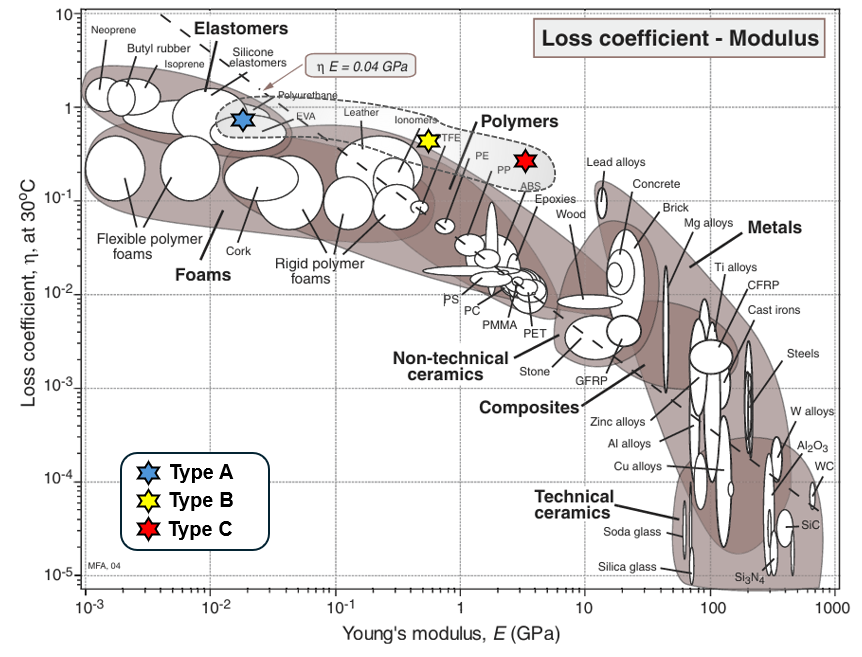


**Figure S8.** Ashby chart comparing the loss coefficient and Young’s modulus of the developed PUSA-HUA resins with those of conventional materials.

**Dynamic Bond Configuration of PUSA-HUA Resins with FTIR and Raman Analysis**

The viscoelastic damping properties of PUSA-HUA resins are derived from the dynamic bonds incorporated within the resin matrix. The dynamic properties of PUSA-resin arise from disulfide (S-S) bonds introduced by the chain extender and hydrogen bonding interactions primarily facilitated by hydroxyl (-OH) groups from the HEA diluent, with additional contributions from urethane (-NH) donors and carbonyl (-C=O) acceptors. These two dynamic bonds interact with the backbone of PUA, synergistically creating a damping effect.

**Figure S9**a shows FTIR spectra of PUSA-resin and HUA-resin, and Figure S9b shows spectra of the three resin compositions, highlighting the -OH (3436 cm^-1^) and -NH (3350 cm^-1^) stretching vibrations. Both -OH and -NH groups participate in dynamic hydrogen bonding, which plays a critical role in the resin's viscoelastic properties. In urethane systems, reversible hydrogen bonds between -NH and carbonyl (or -OH) groups, particularly in long-chain segments, contribute to the material's ability to adapt to stress. As expected, the intensity of the -OH peak is highest in the composition with a higher PUSA-resin content (Type A, PUSA-resin: HUA-resin = 9:1, weight ratio) due to the more significant contribution of HEA as a diluent. However, the -NH peak intensity is higher in HUA-resin-rich compositions, indicating a greater concentration of urethane (-NH) groups in these formulations.

Figure S9c presents the Raman spectra of the three resin compositions, spanning from 1000 to 400 cm^-1^. The peak at 646 cm^-1^ corresponds to the C-S stretching vibration, a characteristic signature of the disulfide-containing chain extender in PUSA-resin. The peak at 510 cm^-1^ is attributed to the S-S bond stretching, confirming the presence of dynamic disulfide bonds within the resin structure. As the PUSA-resin content increases (moving from a PUSA-resin:HUA-resin ratio of 4:6 to 9:1), the intensity of these peaks gradually decreases. This trend reflects the reduced density of disulfide bonds because of the lower proportion of PUSA in the resin formulation.


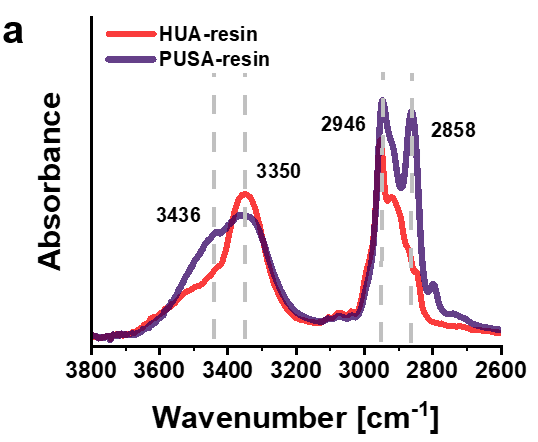

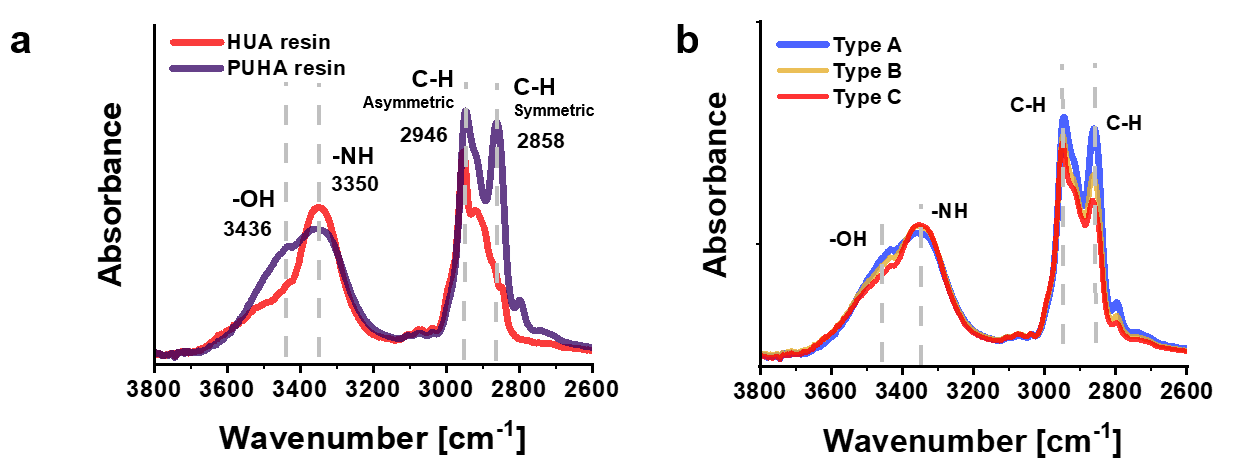

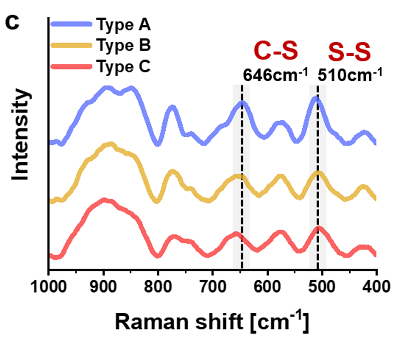


**Figure S9**. Dynamic bond configurations of PUSA-HUA resins with FTIR and Raman analysis. (a) FTIR spectra of PUSA-resin and HUA-resin. (b) FTIR spectra of resin mixtures with varying PUSA-resin: HUA-resin compositions (Type A, B, and C), highlighting the -OH stretching vibration at 3436 cm⁻¹ and -NH stretching vibration at 3350 cm^-1^, which indicate hydrogen bonding interactions. (c) Raman spectra showing C-S (646 cm^-1^) and S-S (510 cm^-1^) stretching vibrations, confirming the presence of dynamic disulfide bonds.

**DLP 3D Printing using Various Compositions of PUSA-HUA Resins.**

The PUSA-HUA resin formulations were applied to DLP 3D printing. **Figure S10**a presents the viscosity measurements of the three PUSA-HUA resin types. While all resins exhibit moderately high viscosity, their values remain below 3000 cP, which is generally considered suitable for DLP 3D printing. In addition, the observed shear-thinning behavior further confirms their printability from a rheological standpoint. Figure S10b shows the cured depth measurements of the three types of PUSA-HUA resins as a function of the applied light energy dose. These values represent the averages of three repeated measurements. The light intensity used in this measurement was 4.1 mW cm^-2^, which corresponds to the maximum grayscale value (255). Based on common guidelines for DLP printing, an appropriate cured depth is typically 1.5 to 3 times the layer thickness, which corresponds to approximately 0.15–0.30 mm. Figure S10c presents the results of fitting these measurements to Jacob’s law equation. From the fitting, the penetration depths (D_p_) were estimated to be 0.215, 0.222, and 0.243 mm for Type A, B, and C resins, respectively, and the corresponding critical energies (E_c_) were 5.03, 4.83, and 4.95 mJ cm^-2^.

**Figure S11** demonstrates the DLP 3D-printed structures: (a) a frog model, (b) an octet truss, and (c) a traditional pattern dish, representing structures that demand high printing precision. All structures were fabricated under consistent DLP printing conditions, with an energy dose of 13.8 mJ cm^-2^ and a layer thickness of 100 μm. The results highlight the ability of PUSA-HUA resins to create intricate and precise structures with a resolution of less than 1 mm.


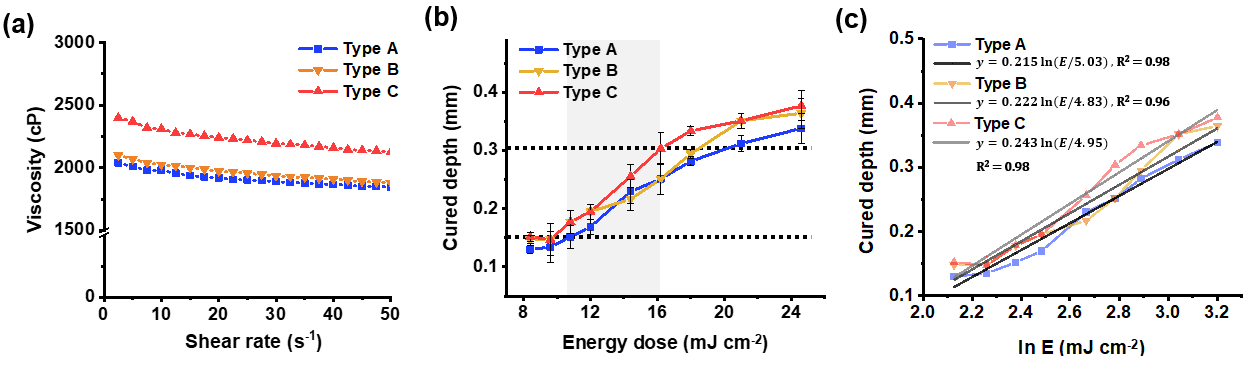


**Figure S10.** DLP 3D Printing using three types of PUSA-HUA resins. (a) Viscosity measurements of Type A, B, and C as a function of shear rate. (b) Cured depth as a function of energy dose for Type A, B, and C. (c) Fitted Jacobs’ working curves for each resin type.

*
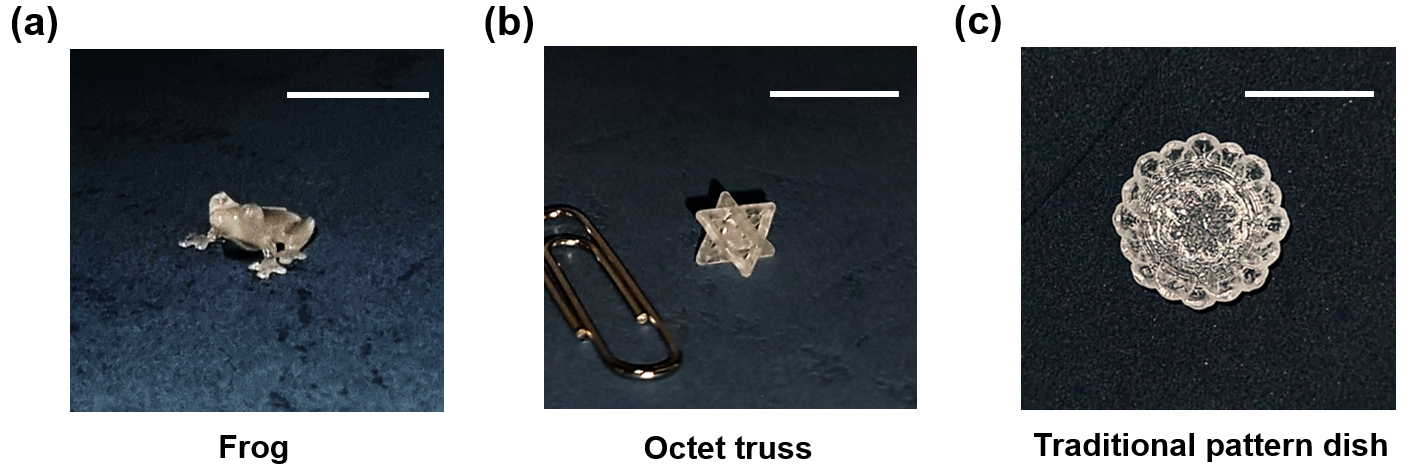
*

**Figure S11.** DLP 3D printed structures using the PUSA-HUA resin: (a) Frog, (b) Octet truss, and (c) Traditional pattern dish. All scale bars represent 10 mm.

**S4. PUSA-HUA Resin Application for g-DLP Printing**

**PUSA-HUA Resins Applicable for g-DLP Printing**

PUSA-HUA resins were utilized in g-DLP printing to evaluate their gradient mechanical properties. Samples were fabricated at grayscale levels ranging from 135 to 255 in intervals of approximately 20 units, and their mechanical properties were assessed through at least three repeated tensile tests. **Figure S12** presents the stress-strain curves for (a) Type A, (b) Type B, and (c) Type C resins, while **Table S9, Table S10**, and **Table S11** summarize the elastic modulus, yield strength, and failure strain at each grayscale level for Type A, Type B, and Type C, respectively. The variation in light intensity with grayscale level is provided in **Table S8**. These values were obtained by placing a photometer on the projection window and averaging the measurements taken at three different positions. The printing conditions included a layer thickness of 100 μm and an exposure time of 3.9 seconds per layer, corresponding to an energy dose of 15.8 mJ cm^-^² per layer at maximum intensity. Calculated energy doses for other grayscale levels are also listed in Table S8. Within the applied energy dose range, resin types A, B, and C exhibited cured depths of approximately 1.3 to 3 times the layer thickness (Figure S10). Given that the recommended energy dose range corresponds to cured depths of 1.5 to 3 times the layer thickness, our experimental results confirm that this parameter window enables stable interlayer stacking while preventing interfacial debonding from under-curing and dimensional inaccuracies from over-curing.


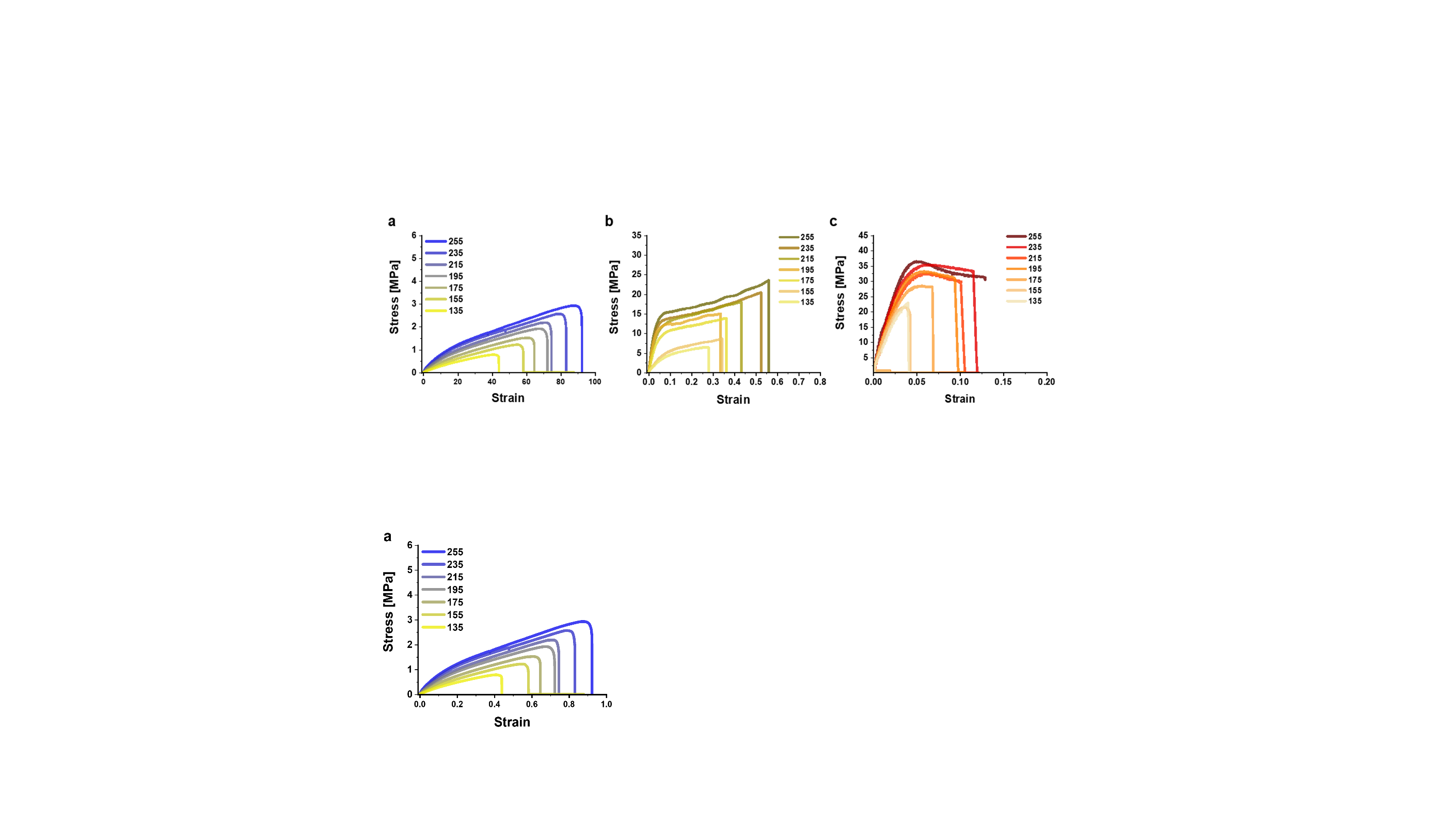

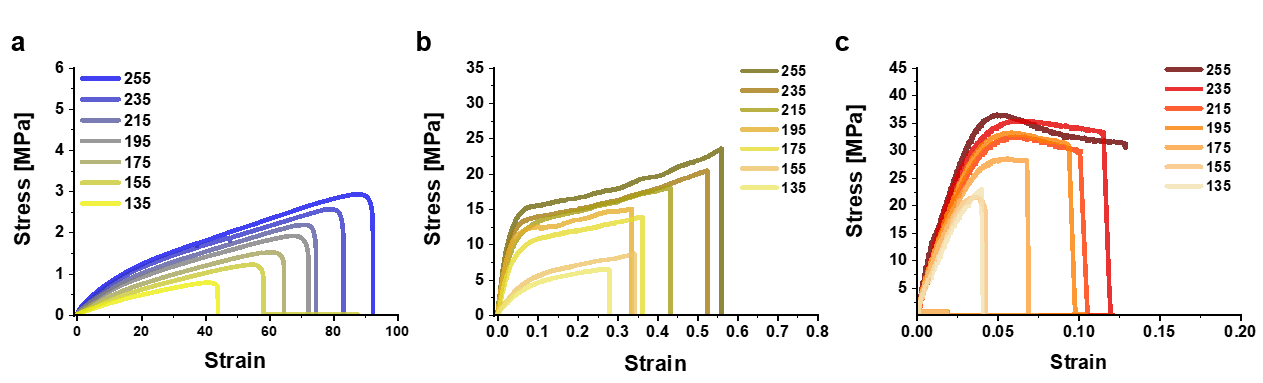


**Figure S12**. Stress-strain curves of PUSA-HUA resin across seven grayscale levels. (a) Type A, (b) Type B, and (c) Type C, demonstrating the gradient mechanical properties achievable with g-DLP printing.

**Table S8.** Grayscale-dependent light intensity in the DLP system and the corresponding energy dose imposed per layer.

| Grayscale | Intensity  (mW cm^-2^) | Energy dose  (mJ cm^-2^) |
| --- | --- | --- |
| 255 | 4.1 | 15.8 |
| 235 | 3.7 | 14.3 |
| 215 | 3.3 | 13.0 |
| 195 | 3.0 | 11.8 |
| 175 | 2.7 | 10.6 |
| 155 | 2.4 | 9.4 |
| 135 | 2.1 | 8.1 |

**Table S9.** Mechanical properties of DLP 3D-printed samples of resin Type A at various grayscale levels.

| Grayscale | Elastic modulus  [MPa] | Failure Strain  [mm mm^-1^] | Tensile strength  [MPa] | | Toughness  [MJ m^-3^] |
| --- | --- | --- | --- | --- | --- |
| 255 (Max) | 8.298 ± 0.550 | 0.811 ± 0.033 | 2.865 ± 0.040 | 1.610 ± 0.080 | |
| 235 | 7.073 ± 0.475 | 0.805 ± 0.055 | 2.604 ± 0.309 | 1.370 ± 0.240 | |
| 215 | 6.794 ± 0.186 | 0.721 ± 0.022 | 2.220 ± 0.085 | 1.106 ± 0.079 | |
| 195 | 5.887 ± 0.113 | 0.664 ± 0.005 | 1.907 ± 0.026 | 0.888 ± 0.011 | |
| 175 | 4.758 ± 0.090 | 0.625 ± 0.022 | 1.576 ± 0.050 | 0.663 ± 0.043 | |
| 155 | 3.936 ± 0.368 | 0.520 ± 0.076 | 1.208 ± 0.169 | 0.460 ± 0.110 | |
| 135 | 2.833 ± 0.050 | 0.393 ± 0.010 | 0.723 ± 0.049 | 0.195 ± 0.011 | |

**Table S10.** Mechanical properties of DLP 3D-printed samples of resin Type B at various grayscale levels.

| Grayscale | Elastic modulus  [MPa] | Failure strain  [mm mm^-1^] | Tensile strength  [MPa] | Toughness  [MJ m^-3^] |
| --- | --- | --- | --- | --- |
| 255 (Max) | 471 ± 67.9 | 0.479 ± 0.071 | 21.6 ± 2.48 | 8.13 ± 1.63 |
| 235 | 391 ± 42.7 | 0.471 ± 0.089 | 19.5 ± 1.33 | 7.13 ± 1.07 |
| 215 | 350 ± 128 | 0.435 ± 0.123 | 16.8 ± 1.38 | 5.59 ± 1.16 |
| 195 | 370 ± 74.5 | 0.348 ± 0.079 | 17.5 ± 0.62 | 4.94 ± 1.12 |
| 175 | 188 ± 53.8 | 0.358 ± 0.078 | 11.9 ± 1.74 | 3.29 ± 0.99 |
| 155 | 111 ± 24.6 | 0.326 ± 0.052 | 7.90 ± 0.66 | 1.87 ± 0.29 |
| 135 | 96.8 ± 13.9 | 0.262 ± 0.030 | 7.30 ± 0.77 | 1.41 ± 0.18 |

**Table S11.** Mechanical properties of DLP 3D-printed samples of resin Type C at various grayscale levels.

| Grayscale | Elastic modulus  [MPa] | Failure strain  [mm mm^-1^] | Tensile strength  [MPa] | Toughness  [MJ m^-3^] |
| --- | --- | --- | --- | --- |
| 255 (Max) | 1230 ± 157 | 0.158 ± 0.028 | 29.1 ± 2.89 | 4.41 ± 1.02 |
| 235 | 1290 ± 78.9 | 0.106 ± 0.027 | 32.1 ± 1.27 | 3.03 ± 0.81 |
| 215 | 1090 ± 46.8 | 0.103 ± 0.006 | 30.5 ± 1.03 | 2.81 ± 0.17 |
| 195 | 1140 ± 77.6 | 0.069 ± 0.008 | 32.6 ± 0.83 | 1.69 ± 0.27 |
| 175 | 1110 ± 58.6 | 0.042 ± 0.002 | 26.1 ± 2.52 | 0.78 ± 0.06 |
| 155 | 1070 ± 253 | 0.083 ± 0.025 | 22.5 ± 8.33 | 2.02 ± 0.95 |
| 135 | 972 ± 54.1 | 0.036 ± 0.003 | 26.2 ± 1.97 | 0.56 ± 0.12 |

**Degree of Conversion Variance under Grayscale-Controlled Single-Layer Curing**

To evaluate the grayscale-dependent curing behavior of the resin, single-layer samples were fabricated and analyzed using FTIR spectroscopy, allowing the isolation of grayscale-controlled exposure effects without the influence of cumulative curing. Samples were prepared under controlled conditions with a layer thickness of 100 μm, and the grayscale levels ranged from 135 to 255. An energy dose of 15.8 mJ cm^-2^, corresponding to the maximum grayscale value of 255, was used as the reference, with the energy doses for lower grayscale levels proportionally adjusted.

As shown in **Figure S13**a, the intensity of the characteristic acrylate peak at 809 cm⁻¹, corresponding to C=C stretching, progressively decreased with increasing grayscale, indicating higher Degrees of Conversion (DoC) at elevated exposure levels. Baseline correction was applied by aligning the shoulder near 809 cm⁻¹ to enable accurate quantification. The DoC was calculated based on the change in peak intensity, given that the intensity of the 809 cm⁻¹ band, which corresponds to the stretching vibration of the acrylate C=C bonds, is proportional to the concentration of unreacted double bonds in the material:

$$\begin{aligned} \text{DoC} = 1 - \frac{I_{809, \text{cured}}}{I_{809, \text{uncured}}}\#\left( S1 \right) \end{aligned}$$

where $I_{809, \text{cured}}$​ is the peak intensity after curing at a given grayscale level, and $I_{809, \text{uncured}}$​ is the intensity in the uncured resin state.

As summarized in Figure S13b, the DoC reached 0.55 at grayscale 255 and 0.34 at grayscale 135, reflecting a 38% reduction at lower exposure. While these results were obtained under single-layer conditions, even greater differences are anticipated in multi-layered structures. These results demonstrate that grayscale modulation induces significant variation in the degree of curing, which provides a mechanistic explanation for the grayscale-dependent differences in mechanical performance observed in this study. Figure S13c plots the calculated DoC against the implied energy dose for each grayscale level, with the energy doses corresponding to individual grayscale levels provided in Table S8.


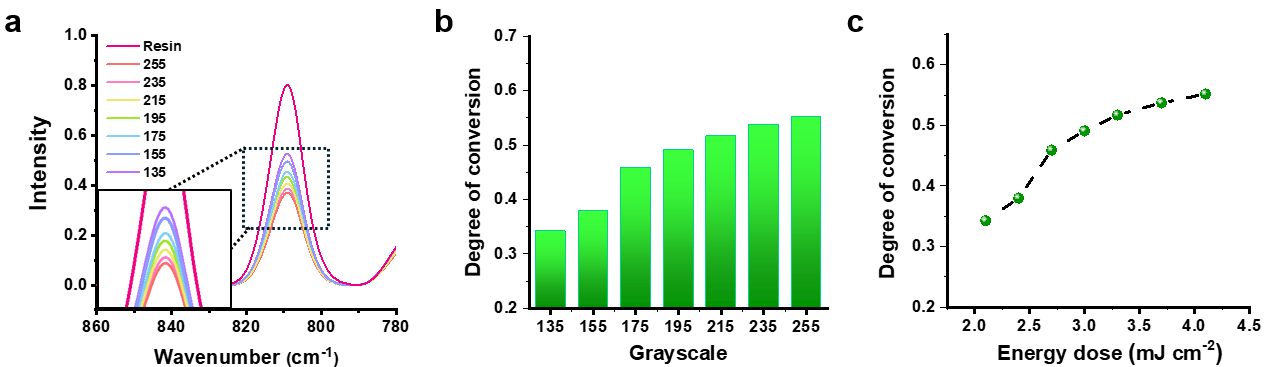


**Figure S13.** (a) FTIR spectra showing the characteristic peak at 809 cm⁻¹ for different grayscale levels (135–255). Calculated degree of cure (DoC) as a function of (b) grayscale level and (c) energy dose.

**Gel Fraction Measurements of g-DLP Printed PUSA-HUA Resins**

DLP technologies typically rely on low molecular weight monomers or oligomers, which often exhibit reduced gelation rates under lower light intensities due to their dependence on crosslinking density. In contrast, the PUSA-HUA resins developed in this study exhibit a high gel fraction (>90%) across the grayscale range of 135 to 255, as confirmed by acetone immersion tests. Disk-shaped specimens (8 mm diameter, 2 mm thickness) were immersed in acetone for three days to induce swelling, followed by drying in a vacuum oven for 12 h. The gel fraction was calculated as the mass ratio of the sample before and after swelling-drying. **Figure S14** presents the gel fraction results for (a) Type A, (b) Type B, and (c) Type C resins, respectively. The high gel fraction is attributed to the formation of sufficient entanglements and the chemically robust urethane bonding characteristic of polyurethane polymers, even under low grayscale conditions.


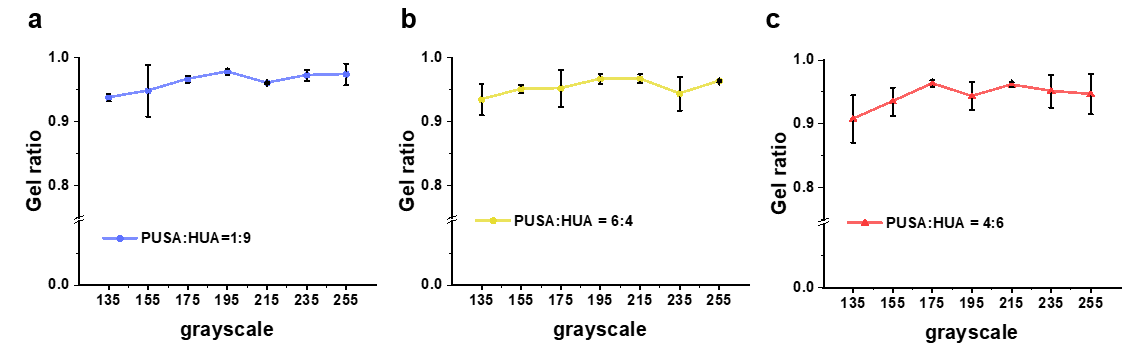


**Figure S1****4.** Gel fraction measurements of three PUSA-HUA resin types across grayscale levels. (A) Type A, (B) Type B, and (C) Type C, demonstrating consistently high gel fractions (>90%) due to robust urethane bonding and polymer entanglements, even under low grayscale conditions.

**Freeform Fabrication of PUSA-HUA Resins with g-DLP**

**Figure S15**a illustrates the 3D structure of a monostable beam and its fabricated form via DLP printing. In Figure S15b, a grayscale gradient is applied to both ends of the beam, which influences its deformation profile and results in a relatively flattened shape. In contrast, Figure S15c shows a gradient applied at the central region, leading to deformation concentrated in the middle under similar loading conditions. These differences show the impact of gradient placement on the structural response. This demonstrates that the buckling behavior of the monostable beam can be finely tuned by adjusting the grayscale distribution, enabling precise control over energy absorption and release without altering the overall shape. By manipulating the initial deformation profile through different gradient configurations, the structural response can be customized for specific applications.


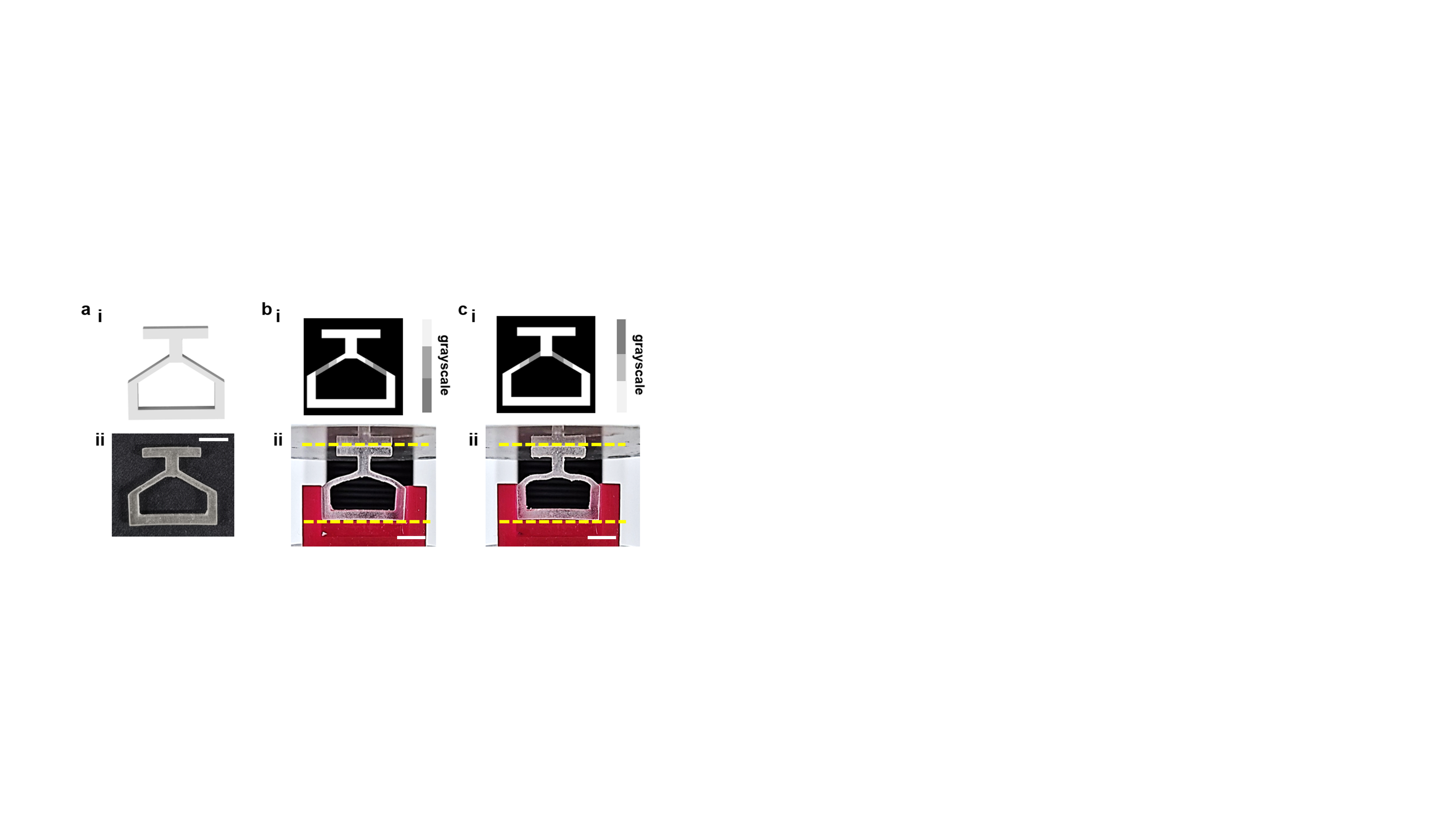


**Figure S15.** Comparison of monostable beam behavior between two gradient structures. (a) The image of i) 3D CAD model and ii) DLP-printed structure. (b) For gradient applied at both ends: i) Illustration of the monostable beam with a gradient, and ii) the compressed beam shape under a compression test. (c) For the gradient applied at the central region: i) Illustration of the monostable beam with a gradient and ii) the compressed beam shape under a compression test. The scale bar is 10 mm.

**Dimensional Variance in g-DLP Printing of PUSA-HUA Resin**

To evaluate the influence of grayscale levels on printing accuracy and dimensional fidelity, we first fabricated cylindrical pillar structures with two different aspect ratios using the Type B resin formulation. The thick pillar had a target diameter of 3.3 mm, while the slender pillar had a target diameter of 0.8 mm, both designed as vertical columns with identical layer thickness (100 μm) and exposure conditions (15.8 mJ/cm² per layer). These two pillar geometries were chosen to represent different levels of structural rigidity and to assess potential shape deviations across varying grayscale levels.

As shown in **Figure S16**, the printed thick pillars exhibited dimensional deviations of approximately +1.5% and −1.5%, while the slender pillars showed deviations of +1.1% and −0.5% relative to their target diameters. When comparing the two extreme grayscale conditions (maximum 255 and minimum 135), the relative size difference between these conditions was measured to be 3.0% for the thick pillar and 1.6% for the slender pillar. While these variations reflect a measurable impact of grayscale level on the final printed size, the magnitude of this change remains small. It does not reach the level typically associated with significant overcuring. In the slender pillars printed at low grayscale (135), a slight bending was observed, which we attribute to reduced stiffness of the softer material during the vertical stacking process. Nonetheless, the overall dimensional fidelity was well-maintained across both pillar types, demonstrating stable feature formation even at the lowest grayscale level.

In addition to the uniform grayscale conditions, we further evaluated the printing fidelity of grayscale-gradient structures to assess whether consistent resolution and dimensional accuracy could be maintained across continuous grayscale transitions. As shown in Figure S16c, a gradient pillar array was designed with three different line widths, stacked vertically across seven grayscale steps from 255 (bottom) to 135 (top). Throughout the transition, vertical resolution was consistently preserved, and no significant distortion or interface blurring was observed, confirming robust grayscale control even in gradient configurations.


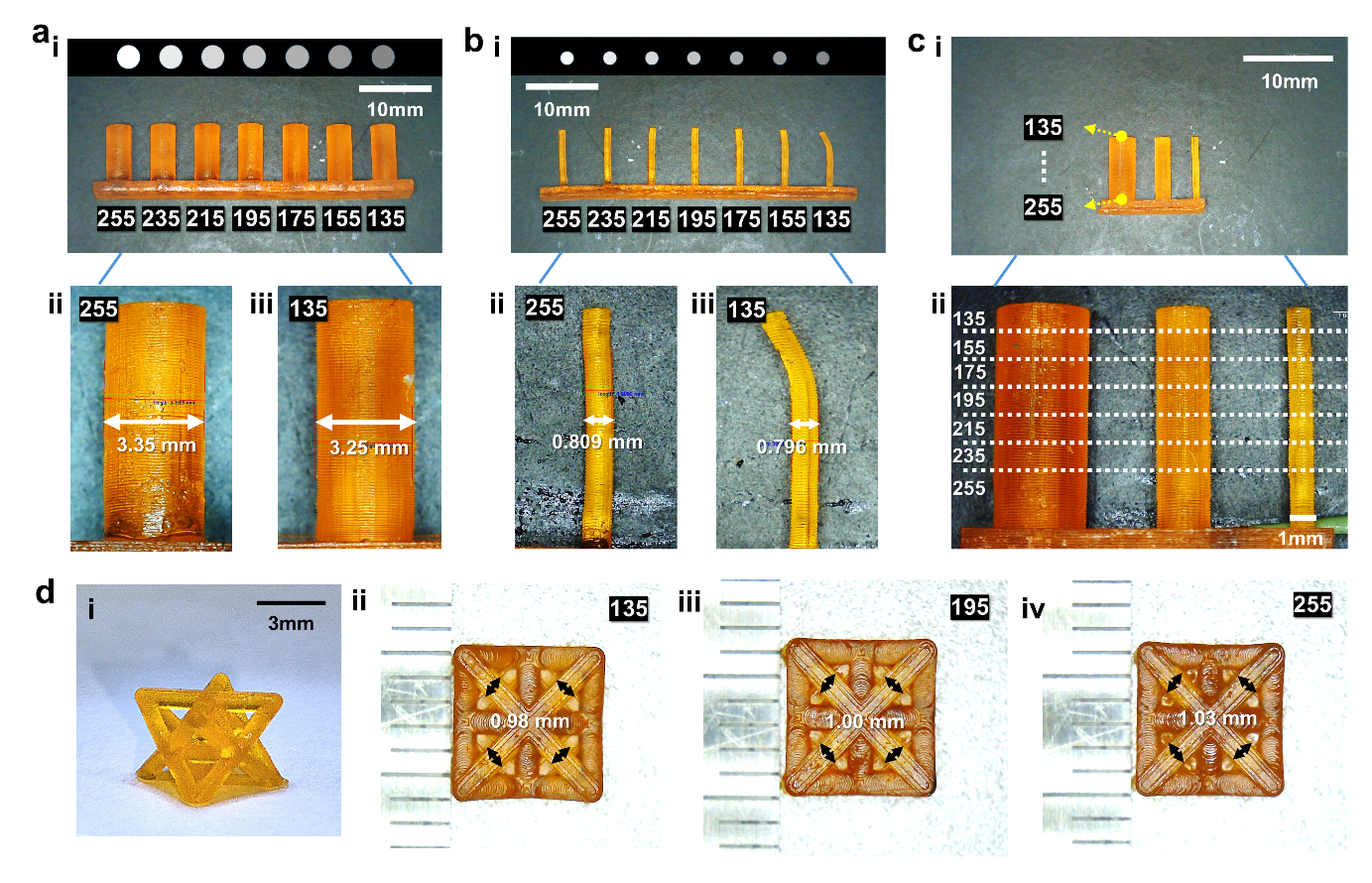


**Figure S16.** Grayscale-dependent printing accuracy and dimensional fidelity in g-DLP printed pillar structures. Printed cylindrical pillars with two different aspect ratios: (a) low aspect ratio (diameter ≈ 3.3 mm) and (b) high aspect ratio (diameter ≈ 0.8 mm), each printed at maximum (255) and minimum (135) grayscale levels. (i) Full array view, (ii–iii) magnified images of the 255 and 135 regions, respectively. (c) Gradient pillar array composed of three different line widths, stacked vertically with 7-step grayscale levels from 255 (bottom) to 135 (top). (d) Octet-truss lattice structure showing (i) 3D-printed tilted view and top views at (ii) grayscale 135, (iii) grayscale 195, and (iv) grayscale 255, with rulers on the left indicating 1 mm per division.

Second, to investigate the effect of grayscale level on fine-featured geometries, we fabricated octet-truss lattice structures under different grayscale conditions. The beam thickness at the top layer was measured at four representative positions, yielding values of 0.98 mm at grayscale 135, 1.00 mm at grayscale 195, and 1.03 mm at grayscale 255. This corresponds to a 5.0% increase between the lowest and highest grayscale levels, with deviations of –2.0% and +3.0% relative to the target thickness of 1.00 mm. While the overall variation remains small, slight corner rounding was observed at higher grayscale levels, likely due to localized overcuring at sharp junctions. These results suggest that, particularly for fine features or concave regions prone to such effects, adjusting the maximum grayscale level or exposure intensity may be necessary to ensure accurate feature formation.

**S5. Gradient Structure Design and Optimization Strategies**

As illustrated in Figure 4a of the main text, the gradient structure design process begins with an analysis of the initial strain distribution in the geometric structure, represented by the color scale, where red indicates the maximum strain and blue indicates the minimum strain. In addition, geometry-based gradient structural design optimization has been proposed, but this design strategy may be less constrained by structural limitations.^[4]^ In this case, for the 2D plane stress model, the maximum principal strain value ($\varepsilon_{\text{1}}$) was used. The strain value for each element, $\varepsilon_{element}$​, is defined as the average strain value for a node ($\varepsilon_{1}^{\left( i \right)}$) over all the nodes contained within the element, as expressed in Equation S2:

$$\begin{aligned} \varepsilon_{\text{element}}=\frac{1}{N}\sum_{i=1}^{N} \varepsilon_{1}^{\left( i \right)}\#\left( S2 \right) \end{aligned}$$

Here, the function that receives the strain value of each element and outputs the corresponding grayscale value is defined as the Gradient design function (Figure S13a), as given in Equation S3:

$$\begin{aligned} G_{element}=f_{g}\left( \varepsilon_{element} \right)\#\left( S3 \right) \end{aligned}$$

To determine the shape of $f_{g}(x)$, Bézier curves are generated by defining a series of reference points $(P_{1}, P_{2}, P_{3},P_{4}, P_{5}, P_{6})$ (**Figure S16**a), which controls the shape of the curve, as expressed in Equation S4:

$$\begin{aligned} B\left( t \right)=\sum_{i=0}^{6} \binom{6}{i}\left( 1-t \right)^{6-i}t^{i}B_{i},\quad0\leq t\leq1\#\left( S4 \right) \end{aligned}$$

The generated Bézier curve provides a parametric representation of the gradient design function, enabling precise control of grayscale values corresponding to the strain distribution. Previous studies have mainly employed monotonically increasing functions, such as exponential and sigmoidal functions, to define the relationship between initial stress and Young’s modulus. While retaining their advantages, this study enhances design flexibility by relaxing strict monotonicity. Instead, a boundary condition ensures that the highest grayscale value is assigned exclusively to the maximum strain point, specifically the final reference point, $P_{6}\left( x_{6},y_{6} \right)$, as expressed in Equation S5:

$$\begin{aligned} P_{6}\left( x_{6},y_{6} \right),\quad y_{6}=\max\{y_{i}\mid i=1,2,\ldots,6\}\#\left( S5 \right) \end{aligned}$$

where $x_{1}=0$, $x_{2}=0$.2, $x_{3}=0.4$, $x_{4}=0$.6, $x_{5}=0.8$, $x_{6}=1$ as fixed positions on the x-axis, and the x values are input normalized values of $\varepsilon_{element}$​. Figure S17b presents the resulting set of Bézier curve-based functions, which serve as the initial data for optimization. The functions exhibit diverse profiles, allowing for the exploration of a wide range of gradient designs tailored to specific mechanical and structural performance requirements.


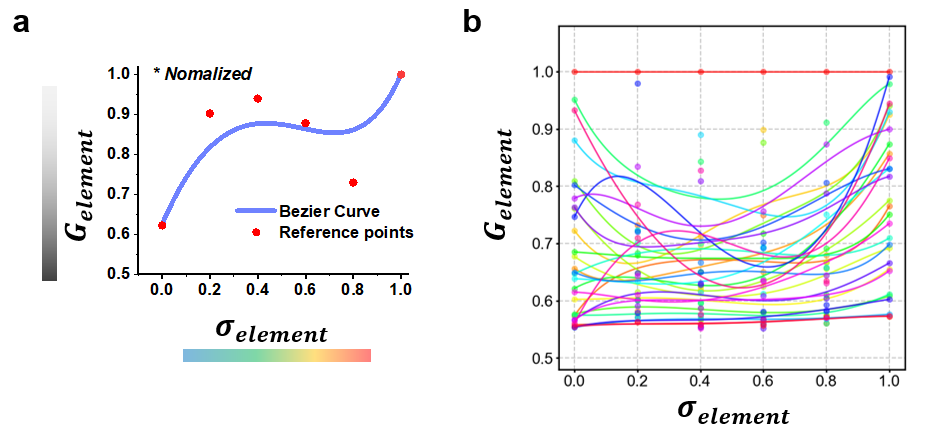


**Figure S17**. Parametric process for generating random gradient design functions using Bézier curves. (a) The Bézier curve-based function generation process. (b) The resulting function set, provides diverse profiles for exploring various gradient designs.

This method enables strain redistribution by lowering Young’s modulus in the surrounding region of a stress concentration point, thereby mitigating stress concentration. Numerical studies on the fracture behavior of functionally graded materials (FGMs) have been conducted, revealing that gradients of elastic modulus can effectively delay crack propagation.^[5,6]^ **Figure S18**a presents an example of a stress-concentrated structure with its mapped grayscale distribution. In this grayscale gradient, the stress concentration point has the highest grayscale value, indicating that the function varies in a monotonic manner locally. Figure S18b idealizes the stress concentration zone using a large side crack for fracture initiation and propagation tests with contour paths for evaluating the J-integral value. This model provides a basis for assessing the effectiveness of the gradient design function used in this study. The J-integral is related to the stress intensity factor in linear elastic deformation, as shown in Equation S6:

$$\begin{aligned} J=\frac{K^{2}}{E^{'}}\#\left( S6 \right) \end{aligned}$$

where $J$ is the J-integral, $K$ is the stress intensity factor, $E^{'}$ is the effective elastic modulus. The stress intensity factor $K$ is related to the nominal stress ($\sigma$), crack length ($a$) and geometric aspect ($f\left( \frac{a}{W} \right))$ as shown in Equation S7:

$$\begin{aligned} K=\sigma\sqrt{\pi a} f\left( \frac{a}{W} \right)\#\left( S7 \right) \end{aligned}$$

By combining these equations, the relationship between the J-integral and nominal stress can be expressed as shown in Equation S8:

$$\begin{aligned} J=\frac{\sigma^{2}\pi a}{E^{'}}{f\left( \frac{a}{W} \right)}^{2}\#\left( S8 \right) \end{aligned}$$

Then, the monotonically increasing gradient function is applied to the simple crack structure shown in Figure S18b. The J-integral value was obtained by averaging the values calculated along these contour paths. The maximum stress was measured at the same point where the highest stress value occurs in the initial state. Since the stress concentration region dominates the J-integral, the nominal stress ($\sigma$) can be regarded as effectively proportional to the maximum stress (σ_max_) in the overall structure. The numerical analysis results for the gradient structure, including J-integral and maximum stress values across 1000 gradient designs within the elastic deformation range, are plotted in Figure S18c. The analysis indicates that the two values are well approximated by a second-order polynomial, which satisfies the relationship described in Equation S7. The gradient function was found to lower the maximum stress compared to the original specimen, thereby reducing the J-integral value. This confirms that gradient design can alleviate stress concentration and ultimately enhance fracture resistance.


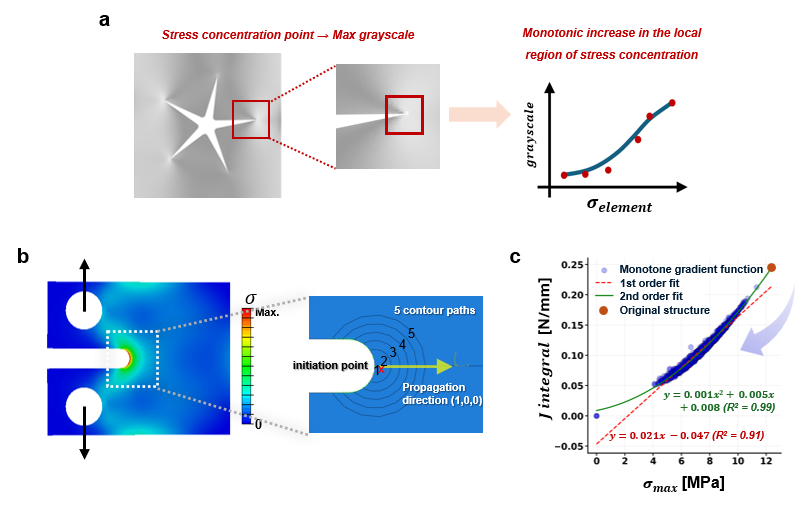


**Figure S18. (**a) Grayscale distribution of a stress-concentrated structure, with the highest value at the maximum stress point. (b) An idealized stress-concentrated model with a side crack for J-integral evaluation in a gradient structure. (c) J-integral and maximum stress trends across 1000 gradient designs applied to the side crack model, showing a second-order polynomial relationship.

The gradient function, constructed using Bézier curves, assigns grayscale values based on the initially measured strain of each element, which are then translated into material properties. To achieve physical material conversion, a fitting process was required to correlate grayscale values with experimentally obtained resin properties.

To optimize the spatial distribution of material stiffness, relationships between grayscale values and elastic moduli were first established using experimental data (Figure 4a). This relationship was used as the primary input for initial design optimization, which assumes linear elasticity. During the optimization process, the grayscale-dependent material assignment was initially performed under the assumption of small deformation. Therefore, full-range stress–strain curves were not directly required at this stage.

However, to enable reliable fracture prediction and ensure that this modeling approach remains valid even under large deformation conditions, we developed a generalized curve generation method capable of representing the nonlinear stress–strain behavior across all grayscale levels. In grayscale-DLP printing using nonlinear materials—such as the resin employed in this study—the mechanical behavior of the printed resin varies markedly with the applied grayscale level, not only in elastic modulus but also in the overall shape of the stress–strain curve.

In this approach, we first experimentally measured the stress-strain curves at two representative grayscale conditions: maximum grayscale (255) and minimum grayscale (135), where the curve shapes were found to be most distinct (**Figure S19**a). We then generated intermediate curves for other grayscale levels by applying a linear combination between these two reference curves, with weighting determined according to the experimentally measured Young’s modulus at each grayscale level. This interpolation was performed point-by-point across the strain–stress data, ensuring that the resulting curves not only reflected the gradual shape transition but also accurately matched the experimentally obtained modulus values (Figure S19b). In addition, failure strain was used as a constraint for each interpolated curve to maintain physical consistency with the measured data.

The imposed constraint on Young’s modulus enabled the interpolation-based curve generation to accurately reflect the actual material behavior (Figure S19c). Toughness values calculated from the interpolated curves were also compared with the experimentally measured toughness, showing reasonably good consistency despite some minor deviations (Figure S19d).

Although the full-range stress-strain curves were not essential for the initial optimization focused on stiffness-dominated design, the development of this curve generation method provides a flexible framework for future applications involving high-deformation or fracture-dominated scenarios. Moreover, this strategy is expected to enhance the generality and adaptability of the FEA–ML-based optimization framework, allowing it to be readily applied to various resin systems with different mechanical characteristics.


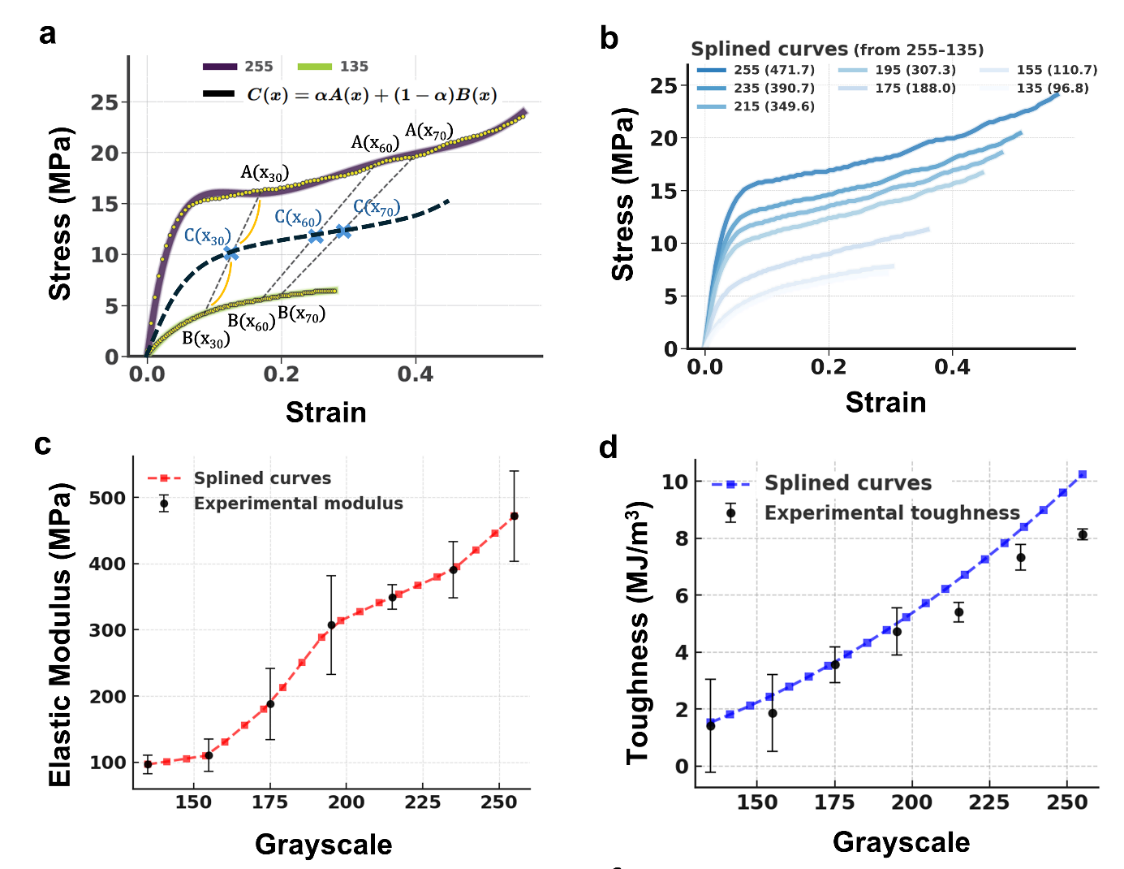


**Figure S19.** (a) Modeling method using Linear combination between two reference curves, weighted by Young’s modulus values. (b) Splined curves generated by this interpolation method for various grayscale. (c) Comparison between the interpolated splined curves and the experimentally measured elastic modulus. (d) Comparison between the interpolated splined curves and the experimentally measured toughness values.

**Comparison of Multi-objective Bayesian Optimization Algorithms**

In this study, three optimization algorithms, illustrated in Figure S20, were explored to simultaneously consider two objective functions: weighted-sum-based single-objective optimization, multi-objective optimization, and a hybrid approach. Among these, multi-objective optimization employs Expected hypervolume improvement (EHVI), a hypervolume-based acquisition function widely used in Bayesian optimization. EHVI quantifies the expected improvement in the hypervolume of the Pareto front by integrating the uncertainty in the objective functions.^[7]^

Single-objective Bayesian optimization combines the two objective functions into a single objective using a weighted sum approach and employs Expected Improvement (EI) as the acquisition function (**Figure S20**a). This method offers advantages such as low computational cost and rapid convergence. Still, it risks failing to explore specific regions of the Pareto front due to its difficulty in global exploration. In contrast, multi-objective optimization uses EHVI as an acquisition function. This approach is known to generate non-dominated solutions across diverse regions and effectively explore the Pareto front (Figure S20b).


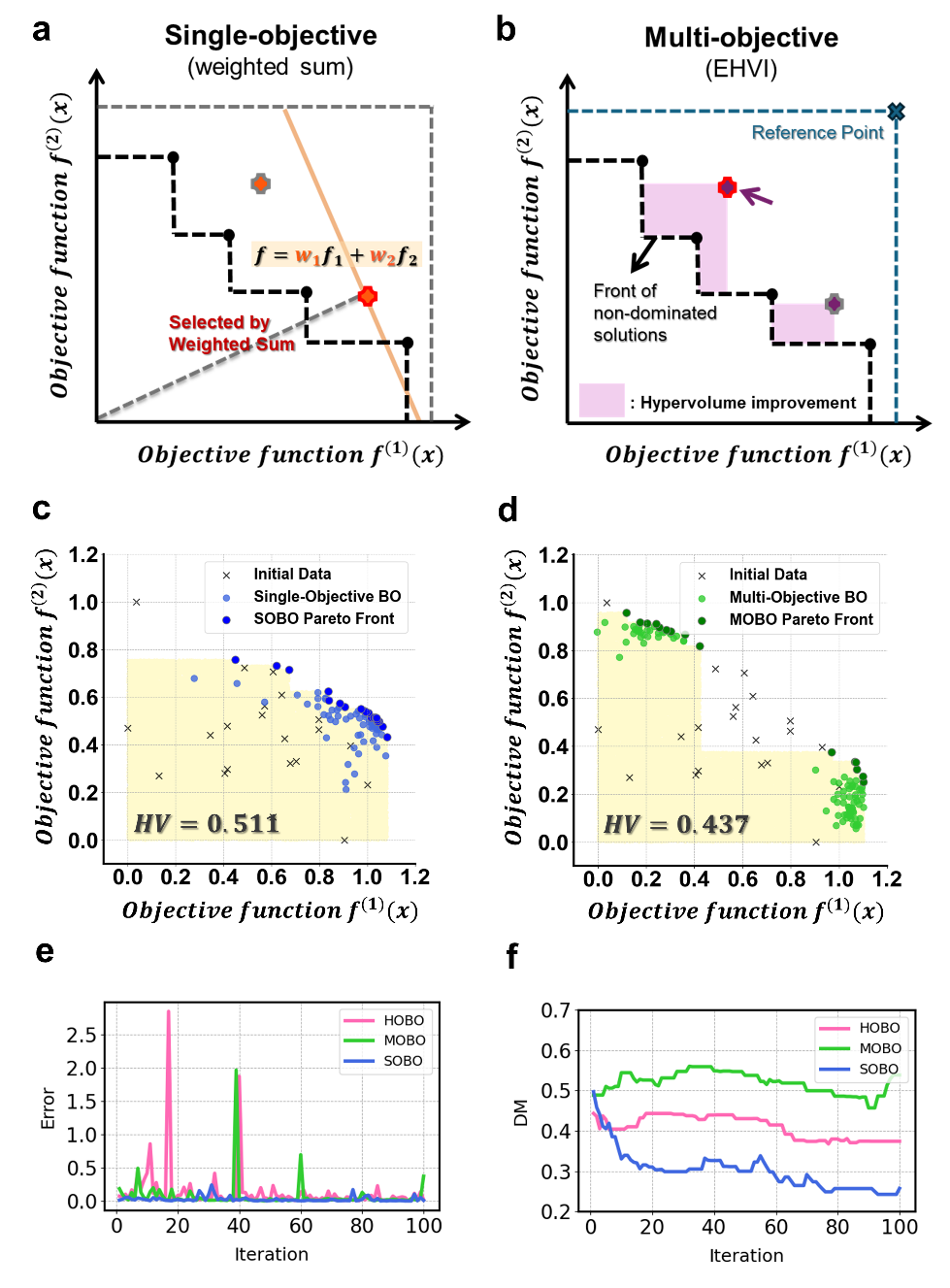


**Figure S20.** Schematics of (a) weighted-sum-based single-objective and (b) multi-objective Bayesian optimization algorithms for multi-objective problems. Optimization results from (c) SOBO, and (d) MOBO, illustrating the BO-generated Pareto frontiers and measured HV metrics. The yellow-colored region represents the dominated solution region used for HV calculation. Comparison of dominated region values across the three algorithms. (e) Error values and (f) DM values over iterations in three different methods, including HBO.

However, in this study, where the two objective functions exhibit characteristics such as one being constrained or a strong trade-off relationship between them, optimization through EHVI may face limited hypervolume improvement potential. Consequently, the contribution of the EHVI term often becomes negligible, leading to an overemphasis on uncertainty and leaving certain regions of the Pareto front unexplored. We suggest that HBO employ a hybrid decision-making step in the BO process using two acquisition functions in a stepwise manner. First, it utilizes the adaptive weighted-sum-based EI for selecting solution candidates. The acquisition function at this stage can be expressed as shown in Equation S9:

$$\begin{aligned} EI\left( x \right)=w_{1}EI_{\text{Obj}_{1}}\left( x \right)+w_{2}EI_{\text{Obj}_{2}}\left( x \right)\#\left( S9 \right) \end{aligned}$$

The weights $w_{1}$ and $w_{2}$ cycle through predefined values during optimization while satisfying $w_{1}+w_{2}= 1$, where $w_{1},w_{2}\in\{0.2,0.4,0.6,0.8\}$ and change cyclically. Then, the final solution for the iteration is determined based on Hypervolume Improvement (HVI). HVI, which quantifies the increase in hypervolume when a new point $f\left( x \right)$, is added to the existing Pareto frontier $P\left( \mathcal{D} \right)$. $f\left( x \right)\text{ }$represents the objective function value for a given input $x$ and is assumed to follow the probability density function $p\left( f\left( x \right) \mid\mathcal{D} \right)$ based on the given data $\mathcal{D}$. HVI can be seen in Equation S10, and EHVI is expressed by the following Equation S11:

$$\begin{aligned} \text{HVI}\left( f\left( x \right), P\left( \mathcal{D} \right) \right) = \text{HV}\left( f\left( x \right)\text{ }\text{∪}\text{ }P\left( \mathcal{D} \right) \right) - \text{HV}\left( P\left( \mathcal{D} \right) \right)\#\left( S10 \right) \end{aligned}$$

$$\begin{aligned} =\int\text{HVI}\left( f\left( x \right),P\left( \mathcal{D} \right) \right)p\left( f\left( x \right) \mid\mathcal{D} \right)df\left( x \right)\#(S11)\# \end{aligned}$$

where $r$ is the reference point, and $f\left( x \right)$ represents the objective function values at $x.$

The overall procedure of the HBO algorithm is summarized in the flowchart shown in **Figure S21**, which outlines the sequential decision-making steps combining weighted-sum-based acquisition and hypervolume-based selection.

For algorithm comparison, we preliminarily explored 500 design functions and formed a set of 30 candidates, including those on the Pareto front. This was conducted because the performance of each function varies significantly across different structures. Given that the dataset inherently involves trade-offs, we aimed to evaluate the suitability of each algorithm for our specific design objectives. HBO achieved a well-distributed effective stiffness and the largest Hypervolume (HV)—a performance indicator in multi-objective optimization that represents the area dominated by Pareto solutions within the reference region—with a value of 0.511, as shown in Figure 4c. This performance surpasses that of both SOBO (Figure S20c) and MOBO (Figure S20d). At this stage, the hypervolume value represents the proportion of the dominated area within the rectangle defined by the Pareto solutions (1.2, 1.2) and (0, 0). Figures S20e and Figure S20f analyze the optimization behavior by examining error and distance metric (DM) values over iterations. Error values remained similar across all methods, indicating no significant differences in prediction accuracy. DM, which represents the distribution of solutions on the Pareto front, initially starts high and decreases over time. SOBO maintained relatively small DM values with a steady decline, reflecting localized exploration. MOBO exhibited a sharp initial increase in DM, indicating strong exploration of extreme values, but lacked sufficient coverage of middle regions and failed to fully converge. In contrast, HBO achieved a balanced increase and convergence in DM values, resulting in a well-distributed Pareto front. The Gaussian Process Regression (GPR) models used as surrogates for the objective functions are independently trained for each response, with detailed model setup and kernel configurations provided in **Table S12.**

In addition, the predictive performance of GPR models in the HBO process was 5-fold cross-validated at each iteration (**Figure S22**). Objective 1 consistently exhibited high accuracy (R² > 0.98), while Objective 2 initially showed lower performance but improved significantly after iteration 20, eventually converging to R² values above 0.96. This indicates that the GPR models maintained reliable performance throughout the BO process.


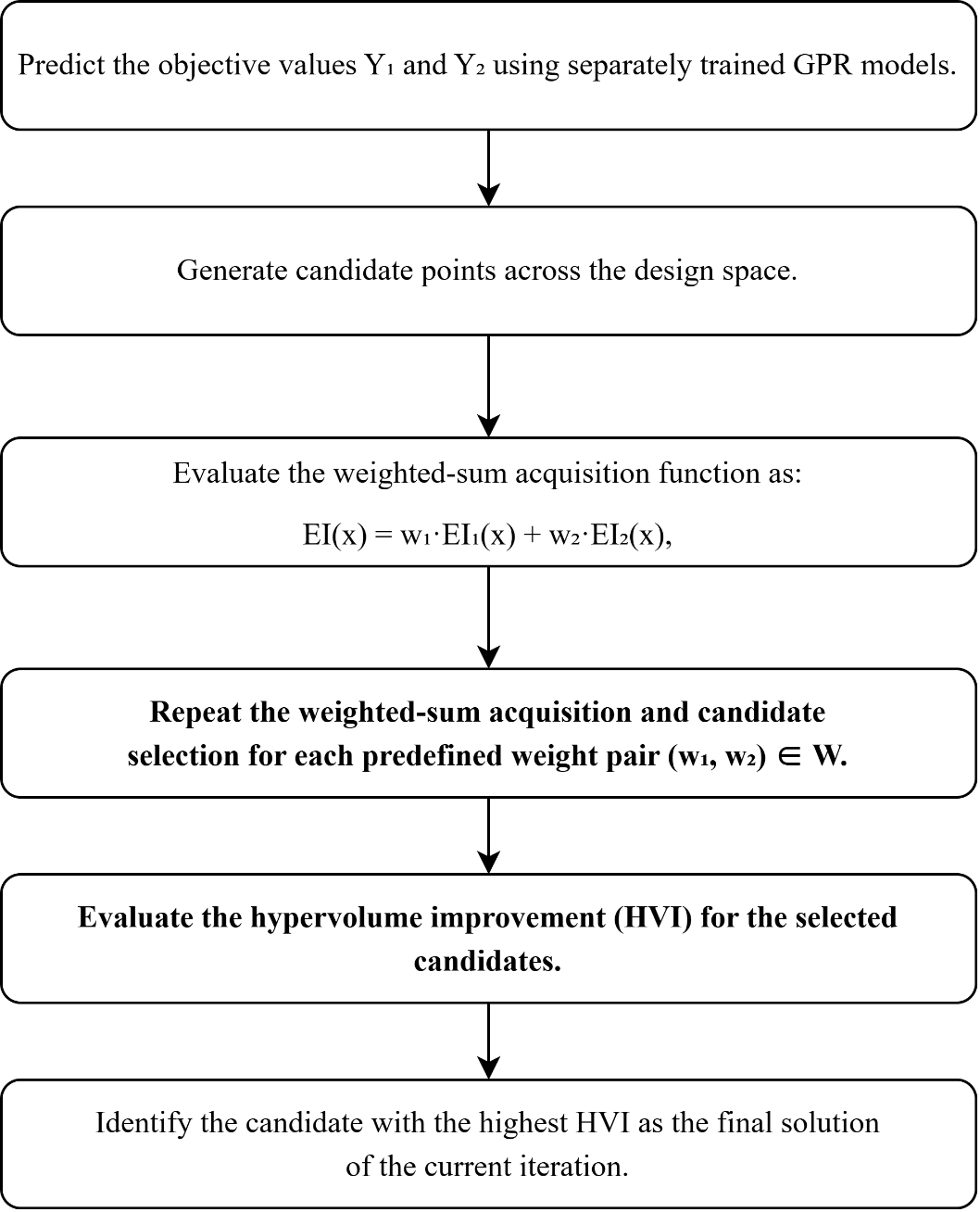


**Figure S21.** Flowchart of the evaluation steps for the HBO algorithm.

**Table S12.** GPR model setup and hyperparameters.

|  | Values |
| --- | --- |
| Kernel | ConstantKernel(1.0, (1e-3, 1e3)) * Matern(length_scale=1.0, length_scale_bounds=(1e-2, 1e2), nu=1.5) |
| Alpha | 0.001 |
| n_restarts_optimizer | 10 |

**Figure S22.** Cross-validation performance of GPR models per iteration.

**S6. Application of Gradient Structure Design and Optimization for 2D Unit Cell Structures**

For the optimization of various 2-D unit cell structures, the Bayesian optimization process repeated over 100 iterations based on 30 initial data points, generating one solution per iteration. The algorithm is implemented using Python with BoTorch.^[8]^ Including the initial data, each 2-D unit cell structure was simulated under plane stress-based tensile testing at every iteration. This process was automated within a single Abaqus-Python framework incorporating the developed HBO algorithm.

**Figure S23**a represents the optimization results for the 2D lattice structure, which is characterized by multiple localized stress concentration points distributed throughout. Figure S23b presents the performance monitoring of the optimization algorithm. Figure S23c visualizes the gradient design functions for the three representative cases: relatively [i] high stiffness, [ii] medium stiffness, and [iii] low stiffness. The key point is that these functions exhibit abrupt changes, rather than a monotonic increase in shape. This indicates the effectiveness of the Bézier curve generation strategy employed in the algorithm. Figure S23d displays the corresponding grayscale images with continuous gradient structures for [i] high stiffness, [ii] medium stiffness, and [iii] low stiffness levels. Grayscale levels in these images can be used as mask images for DLP 3D printing, enabling the precise fabrication of the optimized structures. The 2D lattice structure highlights a scenario with multiple distributed stress concentration points, presenting a more significant challenge compared to simple structures.

**Figure S24** represents the optimization results for the star-shaped hole unit cell. The figure layout follows the same structure as Figure S23. Figure S24a illustrates the Pareto frontiers, highlighting three representative cases—high, medium, and low stiffness—where optimization effects were more pronounced in the medium and low stiffness regions, achieving up to 83.6% strain reduction. Figure S24b illustrates the algorithm’s performance, characterized by steadily decreasing error values and stable DM, confirming convergence. Figure S24c visualizes the gradient design functions for the three selected cases. Figure S24d presents the corresponding gradient structure images, providing a spatial representation of the optimized gradient distributions. The results confirm that gradient structures effectively mitigate extreme stress concentrations induced by the sharp geometry, significantly reducing localized strain.

**Figure S25** illustrates the optimization results for the Rounded hole unit cell. The Pareto frontiers compare initial and BO-generated data, highlighting three representative designs—high, medium, and low stiffness (Figure S25a). The optimization process showed stable performance in DM and error values (Figure S25b). Gradient design functions (Figure S25c) and corresponding structures (Figure S25d) visualize optimized material distributions, which can be used in DLP printing. Results confirm that while curvature design mitigates stress concentration, gradient structures further enhance strain concentration reduction, expanding their real-world applicability.

**Table S13** summarizes the data generation and acquisition setup applied to these 2D unit cell structures. The reference points $P(X_{i}^{fixed},Y_{i})$ of the gradient design function (Equation (S4)) for the three optimal unit cell structures are summarized in **Table S14**. **Figure S26** presents the BO-generated Pareto fronts for each structure, showing that the star-shaped hole achieves a greater reduction in SCF (up to 83.6%) compared to the other structures (46.8% for the rounded hole). This result indicates that the optimized gradient design varies depending on the structural geometry, and the degree of optimization also differs accordingly. These findings demonstrate that the ML-driven BO framework effectively identifies optimal gradient designs tailored to different structural geometries.


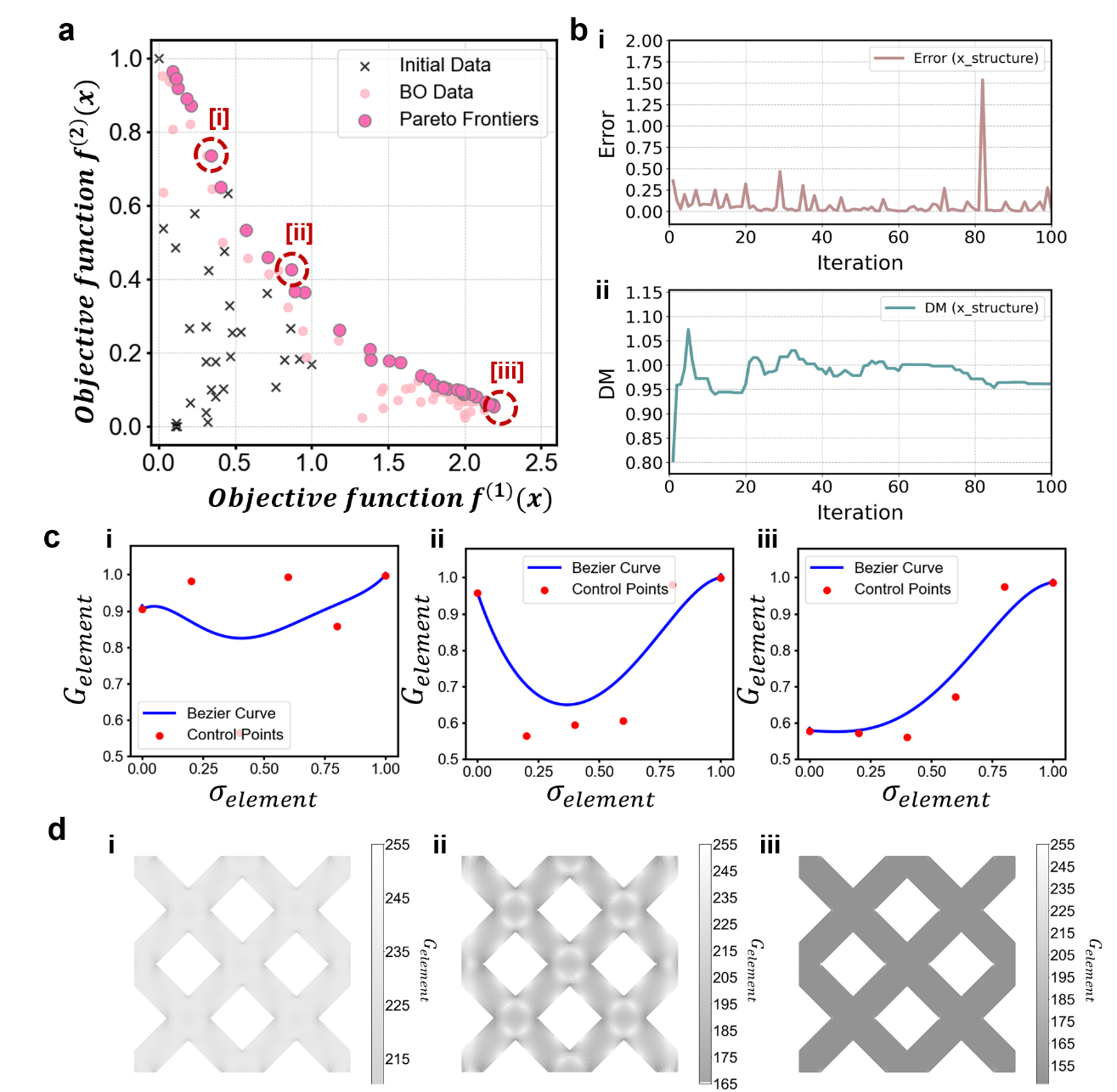


**Figure S23.** Pareto frontiers and gradient optimization results for 2D lattice structures. (a) Distribution of Pareto-optimal solutions with three selected evaluation candidates: i) high stiffness, ii) medium stiffness, and iii) low stiffness. (b) Algorithm performance monitoring during optimization, tracking DM and Error values. (c) Gradient design functions used for the three representative cases, illustrating relative stiffness differences: i) High stiffness structure, ii) Medium stiffness structure, and iii) Low stiffness structure. (d) Gradient structure images for three stiffness levels, demonstrating spatial material distribution and grayscale levels for use as DLP printing masks. The color legend is grayscale level.


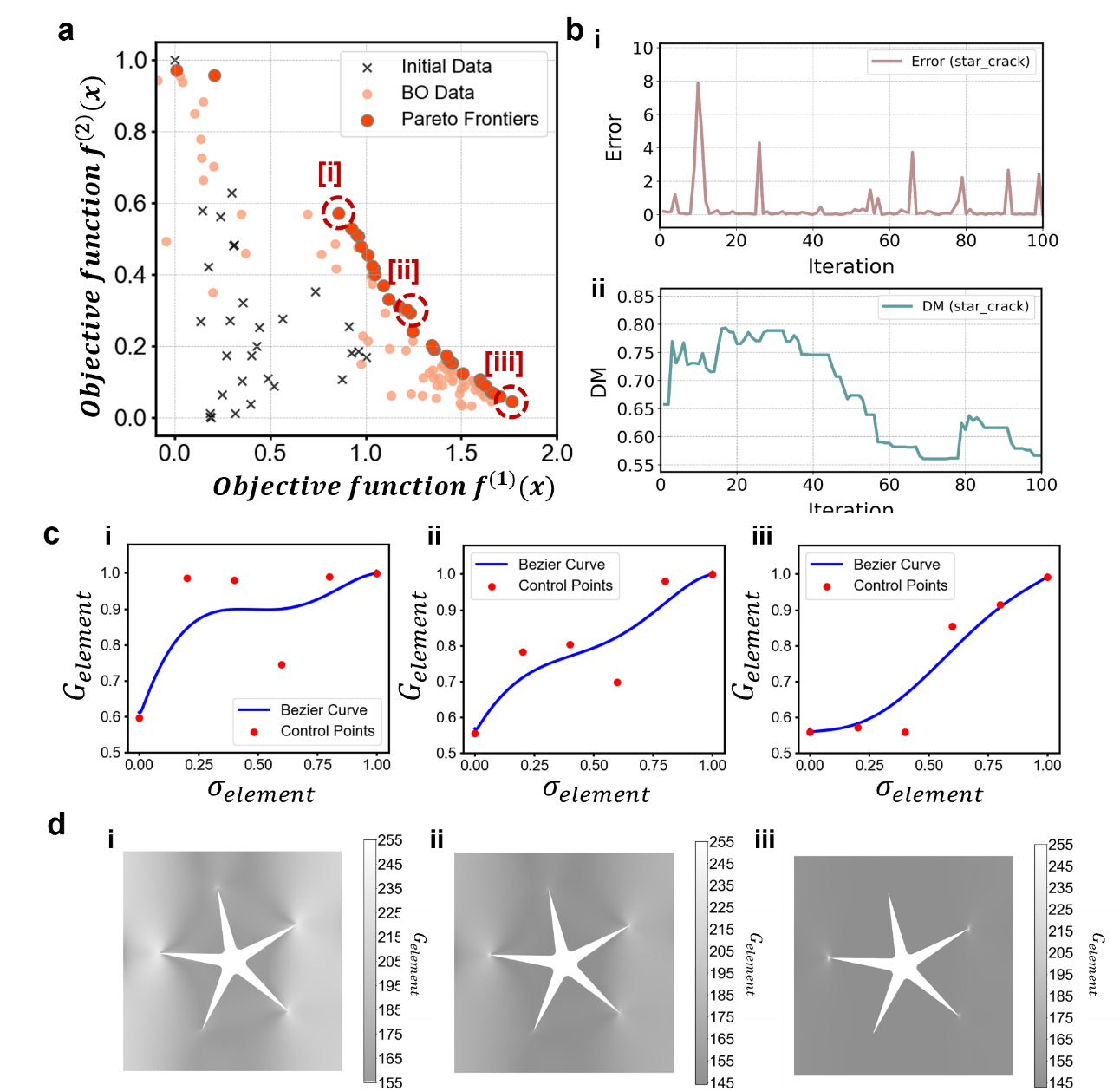


**Figure S24.** Pareto frontiers and gradient optimization results for Star-shaped hole unit cell structures. (a) Distribution of Pareto-optimal solutions with three selected evaluation candidates: i) high stiffness, ii) medium stiffness, and iii) low stiffness. (b) Algorithm performance monitoring during optimization, tracking DM and Error values. (c) Gradient design functions used for the three representative cases, illustrating relative stiffness differences: i) High stiffness structure, ii) Medium stiffness structure, and iii) Low stiffness structure. (d) Gradient structure images for three stiffness levels, demonstrating spatial material distribution and grayscale levels for use as DLP printing masks. The color legend is grayscale level.


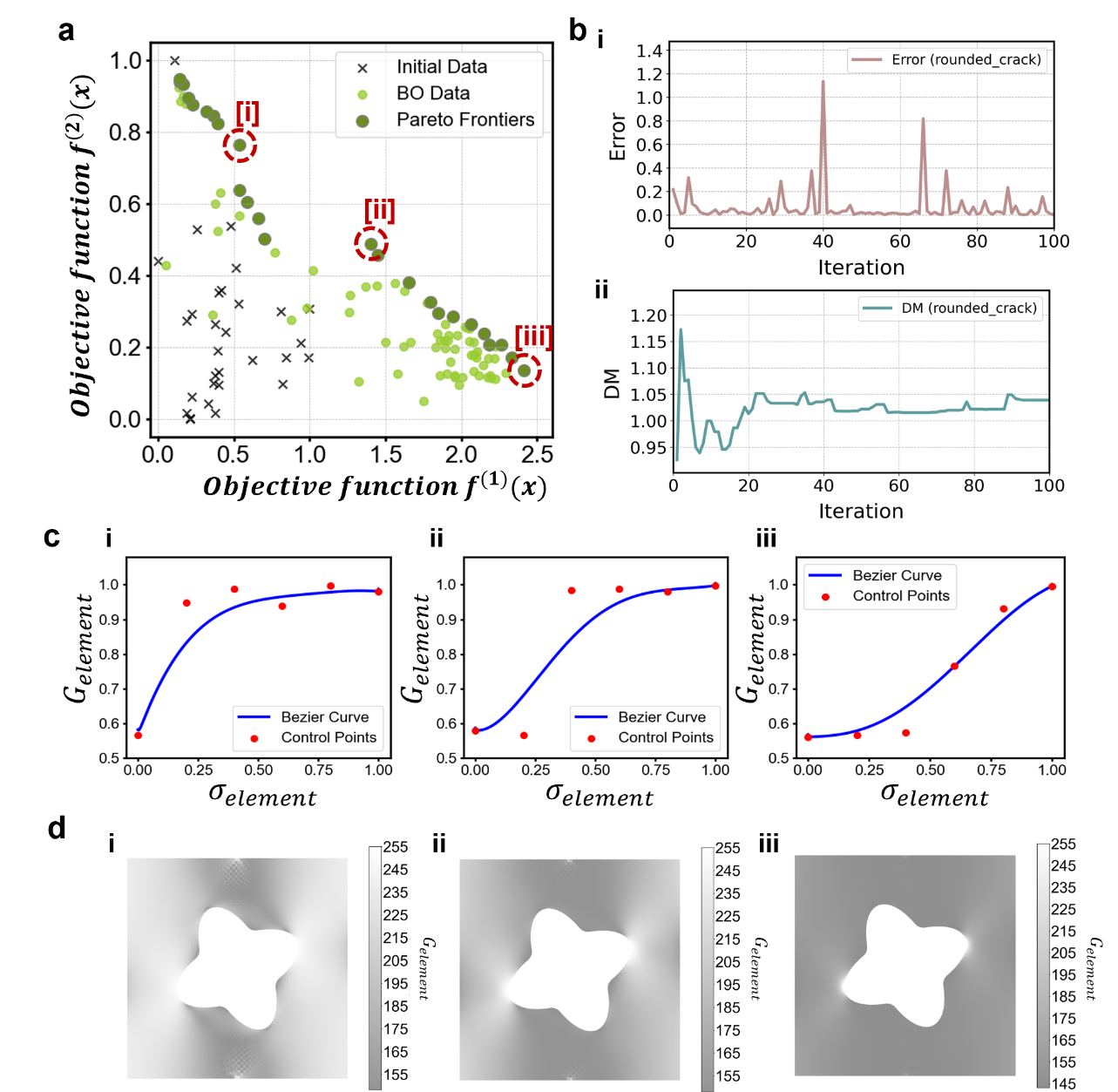


**Figure S25.** Pareto frontiers and gradient optimization results for Rounded hole unit cell structures. (a) Distribution of Pareto-optimal solutions with three selected evaluation candidates: (i) high stiffness, (ii) medium stiffness, and (iii) low stiffness. (b) Algorithm performance monitoring during optimization, tracking DM and Error values. (c) Gradient design functions used for the three representative cases, illustrating relative stiffness differences: [i] High stiffness structure, [ii] Medium stiffness structure, and [iii] Low stiffness structure. (d) Gradient structure images for three stiffness levels, demonstrating spatial material distribution and grayscale levels for use as DLP printing masks. The color legend is grayscale level.

**Table S13.** Summary of data generation and acquisition

| Parameter | Value / Description |
| --- | --- |
| number of Initial data | 30 |
| input data dimension | 6 (each sequence has six variables) |
| output data dimension | 2 (each data point generated by FEA) |
| constraint | Last value (Y_6_) is the maximum in the sequence. |
| Initial sampling | Random selection |
| data generated per iteration | 1 point |
| number of iterations | 100 |
| total data size | 130 (30 initial + 100 generated) |

**Table S14.** $Y_{i}$ values at reference points $P(X_{i}^{fixed},Y_{i})$ used for gradient design curve generation, extracted from the three optimal structures of each unit cell.

| Optimum sample | Y_1_ | Y_2_ | Y_3_ | Y_4_ | Y_5_ | Y_6_ |
| --- | --- | --- | --- | --- | --- | --- |
| Lattice45 | 0.904064 | 0.981765 | 0.563871 | 0.993133 | 0.85863 | 0.997918 |
| Lattice69 | 0.958527 | 0.563248 | 0.594989 | 0.604903 | 0.97983 | 0.999484 |
| Lattice59 | 0.577863 | 0.571282 | 0.559975 | 0.67061 | 0.97478 | 0.985801 |
| Star92 | 0.59518 | 0.986104 | 0.980859 | 0.745135 | 0.98977 | 0.998402 |
| Star49 | 0.555455 | 0.78289 | 0.803495 | 0.698128 | 0.98002 | 0.998793 |
| Star50 | 0.558699 | 0.572115 | 0.558287 | 0.853631 | 0.91482 | 0.99162 |
| Round9 | 0.566195 | 0.94877 | 0.987977 | 0.939365 | 0.99641 | 0.979899 |
| Round53 | 0.579529 | 0.565368 | 0.983823 | 0.987983 | 0.9809 | 0.997026 |
| Round89 | 0.560555 | 0.565905 | 0.574245 | 0.765818 | 0.93096 | 0.995502 |


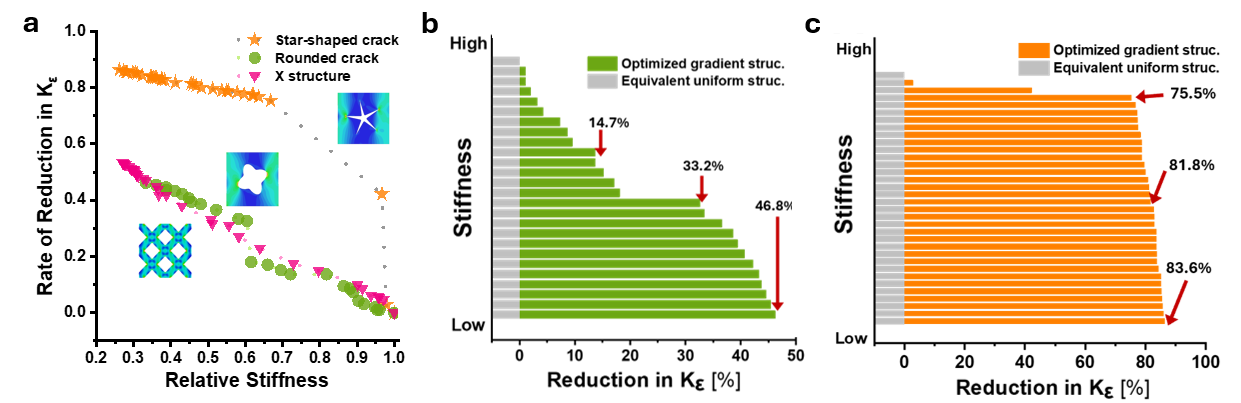


**Figure S26.** Comparative analysis of SCF reduction across gradient-optimized structures. (a) SCF reduction ratios achieved in three structures at various effective stiffness levels. (b) SCF reduction in round-shaped unit cell structures. (c) SCF reduction in star-shaped unit cell structures.

**S7. Validation of 2D Unit Cell Structures**

During the star-shaped hole unit cell structure validation (Figure 5a), the highest stiffness candidate among the three optimized designs (Figure S19) was selected as a representative case for comparison with the equivalent uniform structure. The corresponding grayscale mask images for DLP printing are shown in **Figure S27**a, with (i) the optimized gradient structure and (ii) the equivalent uniform structure. The cyclic loading setup is presented in Figure S27b, and optical images of the specimens after testing are shown in Figures S27c and S27d, confirming delayed crack initiation and enhanced fatigue resistance in the gradient structure.


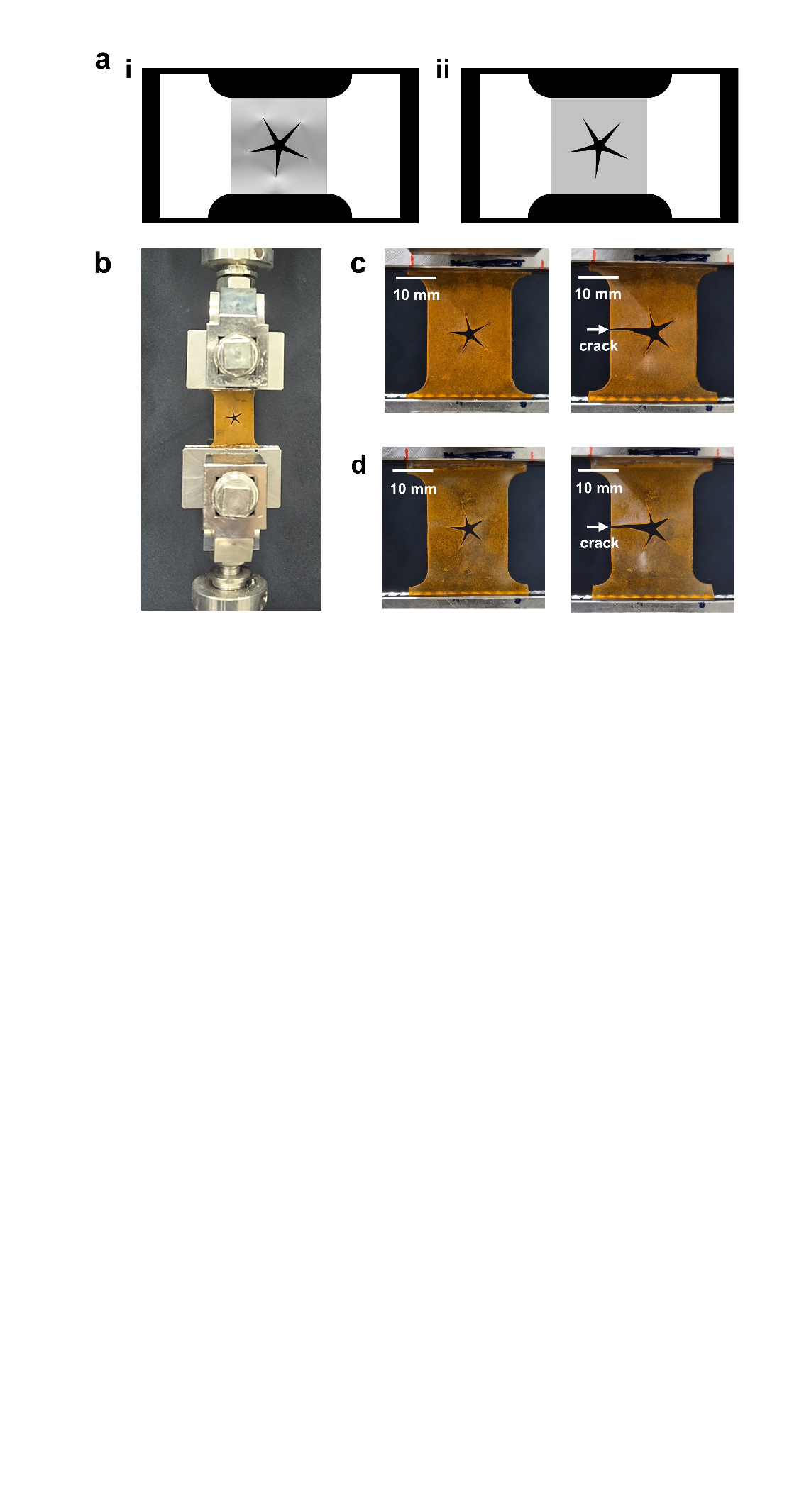


**Figure S27.** Experimental validation of fatigue resistance in the star-shaped hole unit cell structures. (a) Grayscale mask images used for DLP 3D printing: (i) optimized gradient structure and (ii) equivalent uniform structure, both designed with identical geometric configurations. (b) Experimental setup for cyclic tensile loading tests. Images of the (c) equivalent uniform structure and (d) gradient structure before and after cyclic loading test.

As presented in Figure 5 in the main text, the effect of the gradient design in the rounded hole unit cell structure was evaluated through FEA and experimental validation. The material properties were derived from the measured characteristics of the developed resin and assigned as functional forms based on grayscale values. g-DLP 3D-printed specimens using Type B resin were modeled, with material properties fitted to experimental values (Figure S19). An optimum structure with moderate enhancement in $E_{eff}$ and SCF^-1^, marked as sample (ii) in Figure S25, was selected for further analysis. **Figure S28** provides detailed information on the experiment. As shown in Figure S28a, both the gradient and equivalent uniform structures were fabricated using a grayscale mask image within a dogbone specimen to prevent unintended reaction forces at boundary regions. These specimens were produced via DLP printing, as illustrated in Figure S28b, under a printing condition of 15.8 mJ cm^-^² per layer with a layer thickness of 100 μm. Figure S27c and S27d display the initial and after-failure states of the equivalent uniform and gradient specimens, respectively, in the uniaxial tensile test in Figure 5. Figure S28e presents the stress–strain response at a reduced strain rate of 0.5 mm s^-1^, and Figure S28f summarizes the toughness results by integrating the data from Figure 5f and the additional lower strain rate tests. Each strain rate condition was tested in triplicate. Notably, toughness exhibited a clear strain rate dependency, with the gradient structure showing a 72% increase over the uniform structure at 0.5 mm s^-1^. Under slower deformation, the FEA-predicted toughness more closely approximated experimental values; however, the rate-dependent behavior observed experimentally could not be fully captured in the simulation, as the input material properties were derived from single-rate tensile tests.


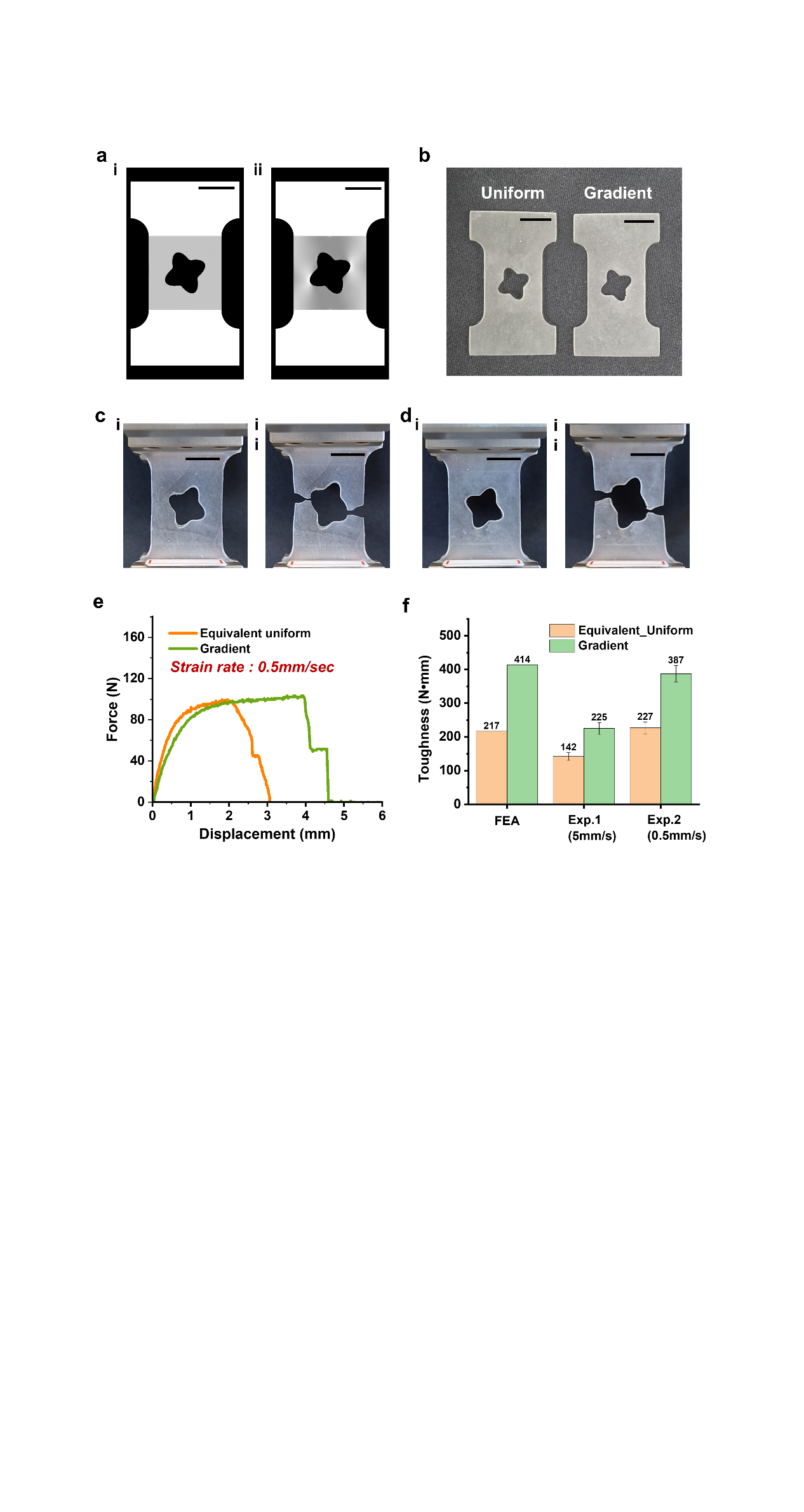


**Figure S28.** Deformation sequences of a dogbone specimen with a rounded hole unit cell structure at the center under tensile testing. (a) Dogbone specimen designed with a grayscale mask image to minimize unintended reaction forces in the grip region. i) Equivalent uniform structure and ii) Gradient structure. (b) Printed specimens of uniform and gradient structures fabricated via DLP printing. (c) Equivalent uniform specimen before i) and after ii) tensile testing. (d) Gradient specimen before i) and after ii) tensile testing. Scale bars are 10 mm for all images. (e) Stress-strain curve of the gradient structure and equivalent uniform structure measured at a reduced strain rate of 0.5 mm s^-1^, highlighting increased toughness at lower deformation rates. (f) Summary of toughness results comparing FEA predictions and experimental data at both 5 mm s^-1^ and 0.5 mm s^-1^, confirming strain rate-dependent toughening and improved agreement with FEA at lower strain rates.

Regarding the round-shaped unit cell structure, we have conducted additional comparative experiments using uniform samples fabricated at various grayscale levels. These experiments clearly confirmed that the toughness enhancement observed in the gradient structures is not simply due to differences in stiffness. The measured stiffness of the uniform samples covered a wide range, from 361 N/mm at the highest grayscale level (255) to 117 N/mm at the lowest level (135). Within this range, the gradient structure exhibited a stiffness of 235 N/mm, while the equivalent uniform structure (grayscale 195) showed a slightly higher value of 246 N/mm. The difference in modulus between the two was approximately 4.5% of the entire adjustable stiffness range, indicating nearly comparable effective stiffness. Thus, the equivalent uniform structure was considered as a valid reference for comparison.

Under this condition, the gradient structure exhibited approximately 60% higher toughness than the equivalent uniform structure. Furthermore, it demonstrated the highest toughness among all uniform structures across the full range of grayscale levels. These results demonstrate that the gradient structure achieves enhanced mechanical robustness beyond simple stiffness modulation.


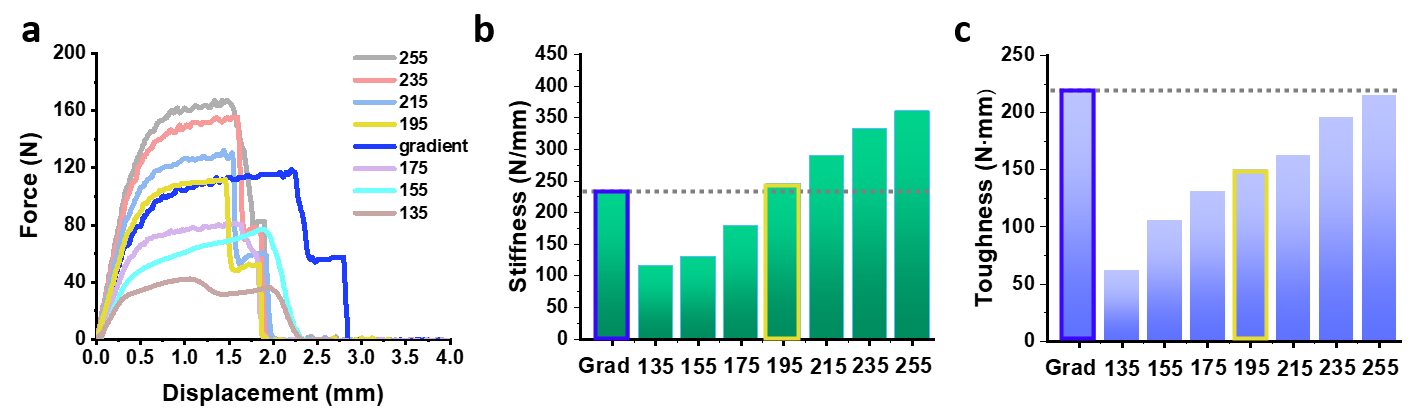


**Figure S29.** Mechanical performance comparison of uniform and gradient specimens fabricated with different grayscale levels: (a) force-displacement curves, (b) stiffness evaluation (slope in 0–0.2 mm displacement range), and (c) toughness evaluation.

When transitioning the 2D-based gradient design and optimization framework to a 3D model, the fundamental design and optimization principles remained the same, while specific technical upgrades were implemented to enhance precision and computational feasibility.

First, we deal with numerical simulation aspects. In the 3D model, the equivalent strain was adopted as a representative strain value, which includes nominal and shear strain, as presented in Equation S12:

$$\begin{aligned} \varepsilon_{\text{eq}}=\sqrt{\frac{1}{2}\left[ \left( \varepsilon_{x}-\varepsilon_{y} \right)^{2}+\left( \varepsilon_{y}-\varepsilon_{z} \right)^{2}+\left( \varepsilon_{z}-\varepsilon_{x} \right)^{2} \right]+3\left( \gamma_{xy}^{2}+\gamma_{yz}^{2}+\gamma_{zx}^{2} \right)}\#\left( S12 \right) \end{aligned}$$

where $\varepsilon_{x}, \varepsilon_{y}, \varepsilon_{z}$are normal strains in the x-, y-, and z-directions, respectively. $\gamma_{xy}, \gamma_{yz}, \gamma_{zx}$ are engineering shear strains in the xy-, yz-, and zx-planes, respectively.

The 3D model was developed to more accurately represent real-world conditions by incorporating interfacial contact and interactions with surrounding objects. This complexity necessitated the use of a unique material constitutive model and a nonlinear one. In terms of the algorithm framework, a discrete grayscale approach with 100 levels was utilized instead of employing a fully continuous gradient across all elements in the 2D model.

Since the gradient design in 3D inherently has lower variance compared to the continuous function-based approach in 2D, maintaining sensitivity to the local extreme value of strain was crucial for effective gradient design. To enhance this sensitivity, the strain evaluation method was refined: instead of using the average nodal strain, the maximum nodal strain ($\varepsilon_{eq}^{\left( i \right)}$) was selected, as presented in Equation S13:

$$\begin{aligned} \varepsilon_{\text{element}}={Max(\varepsilon}_{eq}^{\left( i \right)})\#\left( S13 \right) \end{aligned}$$

This approach synergized effectively with Bézier curves, intrinsically allowing curvature variations, and ensuring effective strain concentration mitigation even in the discrete gradient system.

Second, in the aspect of the Bayesian optimization process, the high computational cost made it impractical to perform numerous iterations as in 2D simulation. To address this, three high-performance cases derived from the optimized unit cell structures were selected as the initial dataset, along with one additional non-gradient case, forming a total of 10 initial datasets (Table S11). Subsequently, 10 BO iterations were conducted, generating 10 BO-derived data points, resulting in a total of 20 simulations to determine the optimal cartilage gradient structure.

**S8. 3D Application Case I: Artificial Human Knee Cartilage**

To realistically simulate the knee joint, a contact model significantly increases computational costs and presents convergence challenges. To address these issues, the quasi-static FEA was structured to include three main components: the femur, cartilage, and tibia. The femur and tibia were modeled as rigid bodies to provide compression and support. The cartilage structure, measuring 45.8 mm in length and 26.5 mm in width, was reverse engineered from a scanned human anatomical model using ANSYS SpaceClaim and Creo Parametric. To balance the trade-off between computational resource limitations associated with nonlinear material properties and contact behavior, and the accuracy of the simulation, the original cartilage material without gradient was simplified to a linear model, with parameters set as follows: Young’s modulus of 8.29 MPa (PUSA-HUA resin Type A), mass density of 1.2 g cm^-3^, and Poisson’s ratio of 0.4. For contact modeling, a no-separation condition with a small sliding allowance was implemented, allowing for mutual deformation between the cartilage and tibia under compression, thereby enhancing realism. The rigid tibia was fixed with a remote displacement constraint to establish a boundary condition, while the rigid femur was assigned a downward displacement boundary condition of 1 mm to maintain the cartilage within a small-deformation regime. Following the simulation, strain data for each element were extracted based on the aforementioned homogeneous material properties and provided to our algorithm framework for material parameter optimization. Once optimized material parameters were obtained, the refined material distribution was reintroduced into the simulation model using APDL command streams, facilitating the simulation of strain distribution within the graded material.

Given that the cartilage-mimicking materials proposed in previous studies typically exhibit moduli in the few MPa range, the primary objective was not to maximize stiffness but to alleviate strain concentration. Therefore, as illustrated in the results, the optimal solution was selected based on the minimum strain concentration factor (SCF), as presented in **Figure S30**a. The detailed design function of the selected optimum is shown in Figure S30b.


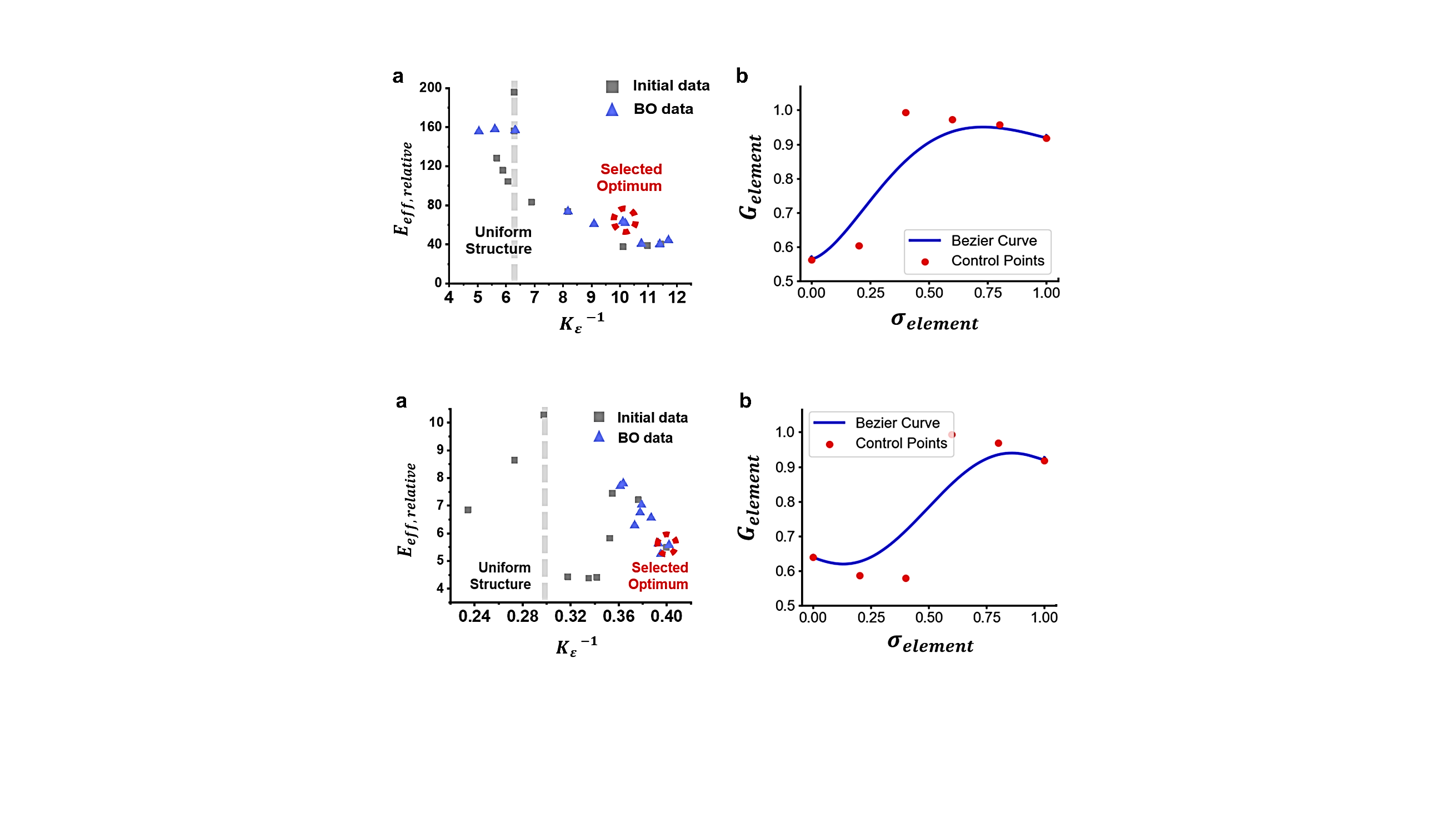


**Figure S30.** Optimization and gradient design results for artificial human knee cartilage. (a) Optimization results in the Pareto frontier and selection of the optimal solution. (b) Gradient design function of the selected optimum design.

**Voxel-based Gradient Structure and g-DLP 3D Printing for Cartilage with Gradient**

In the 3D-based gradient structure, grayscale values were assigned at the voxel level and modeled using Python-based codes.^[9]^ Then, these voxel-based gradient structures were converted into sliced images for g-DLP 3D printing, as shown in **Figure S31**. The fabricated cartilage was fixed onto an ABS-based knee bone-mimicking jig and subjected to cyclic compression testing. Detailed images of this setup are presented in **Figure S32**.


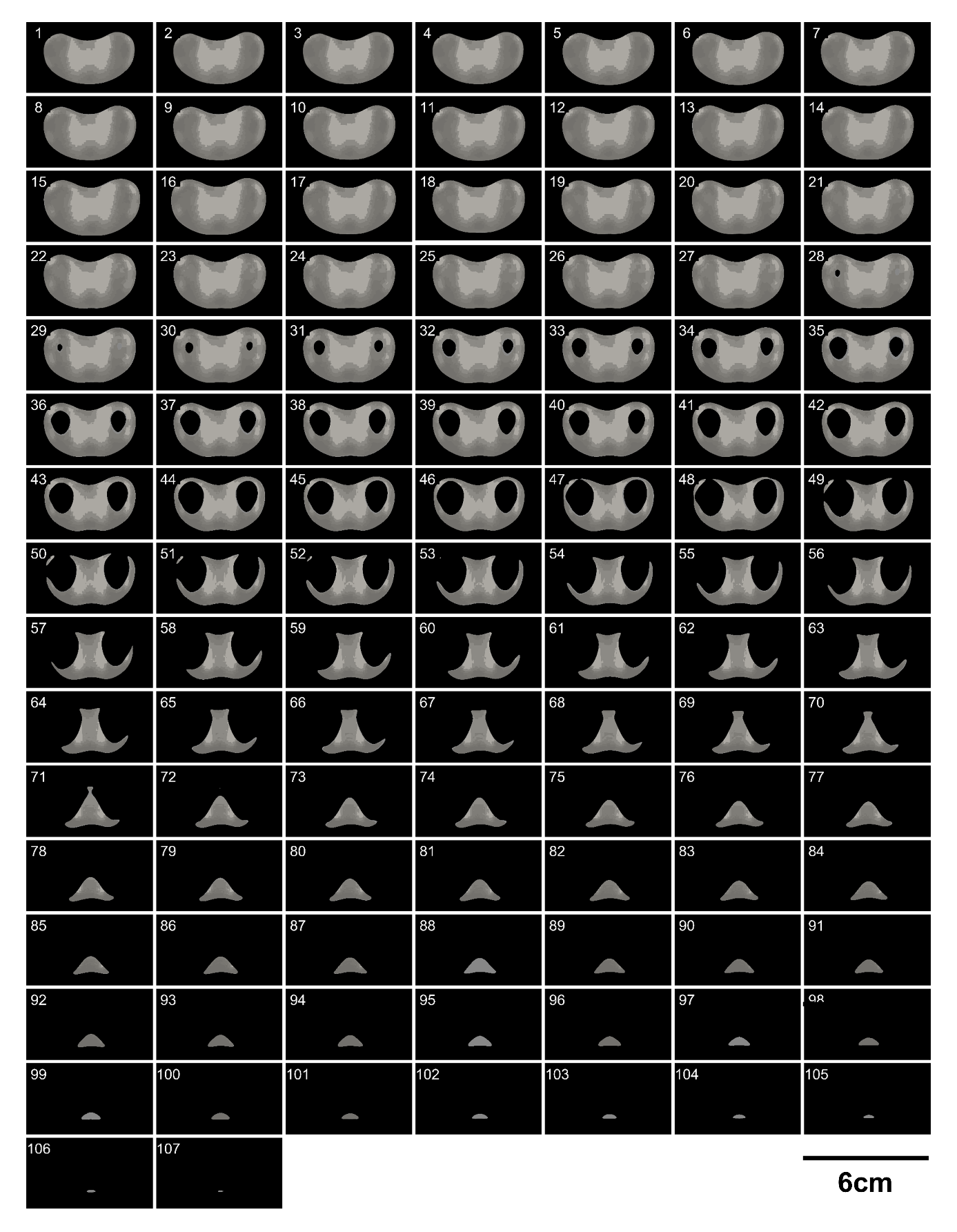


**Figure S31.** Sliced images generated for g-DLP 3D printing of voxel-based gradient structures for artificial cartilage fabrication. All images are 1920×1080 pixels in size.


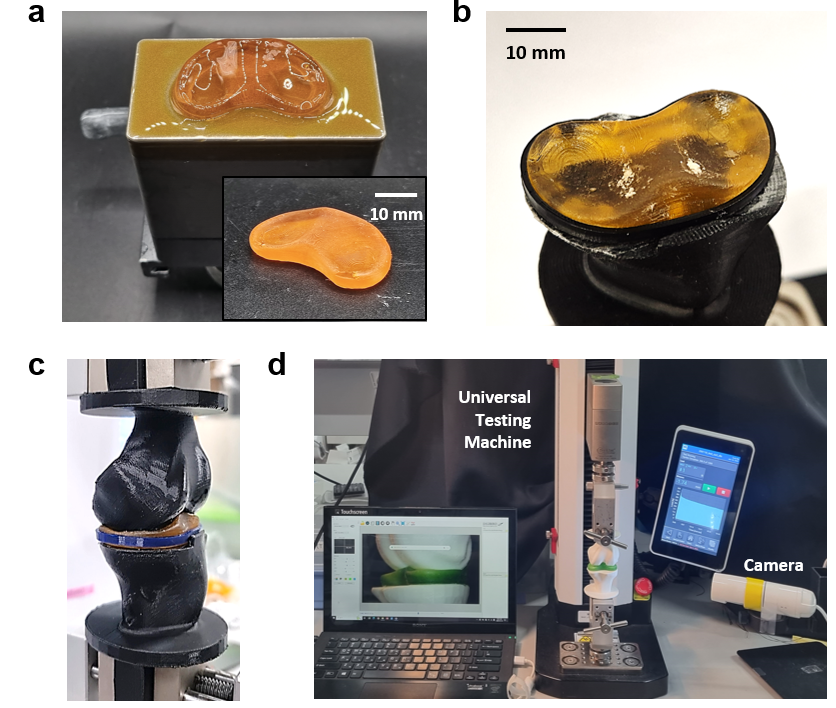


**Figure S32.** Detailed view of the cyclic compression testing setup for knee cartilage. (a) g-DLP printed cartilage structure with gradient, (b) cartilage mounted on the bone jig, (c) configuration of the compression fatigue experiment on the universal testing machine, and (d) overall experimental setup.

**S9. 3D Application Case II: Energy Absorption Beam in Automotive Bumpers**

The gyroid-based automotive energy absorption crash beam was designed to validate the effectiveness of the algorithm framework and customized resin composites in reducing bending strain within architecturally complex geometries. As illustrated in **Figure S33**, the optimization process followed the same methodology as that used for the cartilage case. The selected material is Type B, and the final optimized design achieved an appropriate elastic modulus range for energy absorption during deformation behind the plastic bumper cover.


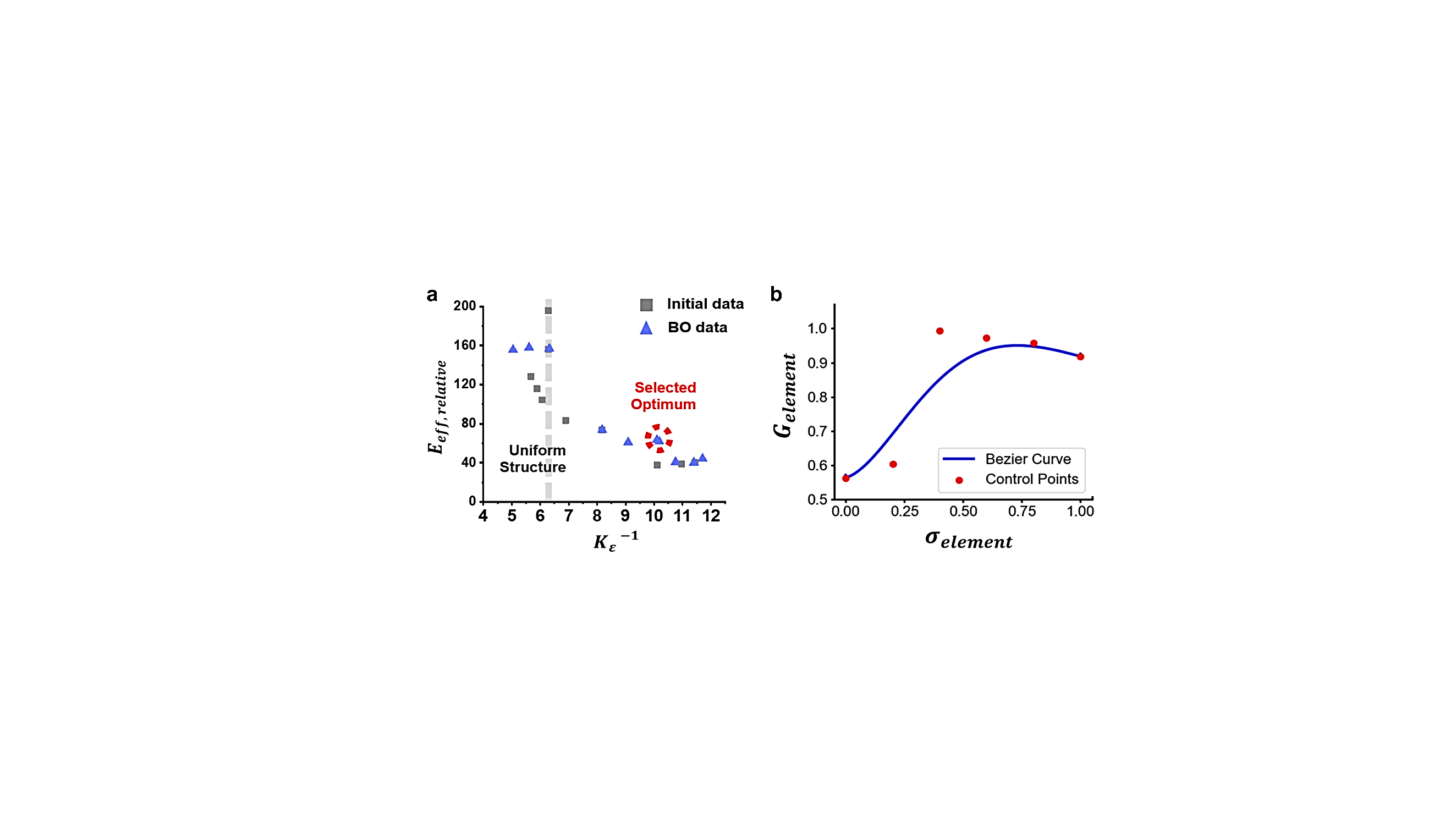


**Figure S33.** Optimization and gradient design results for the automotive energy absorption bumper beam. (a) Optimization results and optimal solution selection with a suitable elastic modulus for energy absorption. (b) Gradient function of the selected optimum design.

Static three-point bending FEA-based numerical simulations were conducted on a gyroid-based automotive energy absorption beam, referred to as the collision beam. The quasi-static simulation model, illustrated in **Figure S34**a, consists of three primary components: a bumper beam, an impactor, and support structures. Since the bumper beam is the focus for deformation and strain analysis, both the support frame and impactor were modeled as rigid bodies, while the bumper beam was designated as a deformable component.

The simulated bumper beam, measuring 56 mm in length and 9.97 mm in width, features a periodic arrangement of gyroid lattice unit cells. This gyroid-based energy-absorbing structure was modeled as a homogeneous material with the following mechanical properties: Young's modulus of 470 MPa, mass density of 1.2 g cm^-3^, and Poisson's ratio of 0.35. The rigid support frame is constrained to maintain fixed boundary conditions through remote displacement. To enhance the convergence of the simulation model while ensuring consistent deflection for strain comparison across different bending structures, a 3 mm downward displacement boundary condition is applied to the rigid indenter. This setup facilitates a static FEA of the three-point bending test for the automotive collision beam based on a homogeneous gyroid structure. To closely approximate real-world conditions, frictional contact is defined between the collision beam, the rigid support frame, and the rigid indenter. Convergence of this highly nonlinear simulation is achieved through careful management of mesh refinement, time step settings, and contact detection parameters. The strain distribution of the homogeneous gyroid-based collision beam serves as the foundation for material modulus optimization within our computational framework, allowing for the design of a gradient gyroid-based collision beam. To ensure valid comparisons, the three-point bending static FE analysis setup for the gradient gyroid-based collision beam mirrors that of the homogeneous material model. The comparison of strain distribution and maximum strain values is presented in Figure 6f of the main text. By utilizing the algorithmic framework and customized material properties, the maximum strain is effectively reduced under the same deflection. As illustrated in **Figure S34**b, the displacement-strain curves for the homogeneous and gradient gyroid-based collision beams reveal significant differences. This outcome validates the effectiveness of the computational approach and customized resin in mitigating strain concentrations during the bending of complex structural designs. The sliced images used for g-DLP 3D printing of the voxel-based gradient gyroid bumper beam are shown in **Figure S35**, where all images are generated at a resolution of 1920 × 1080 pixels.


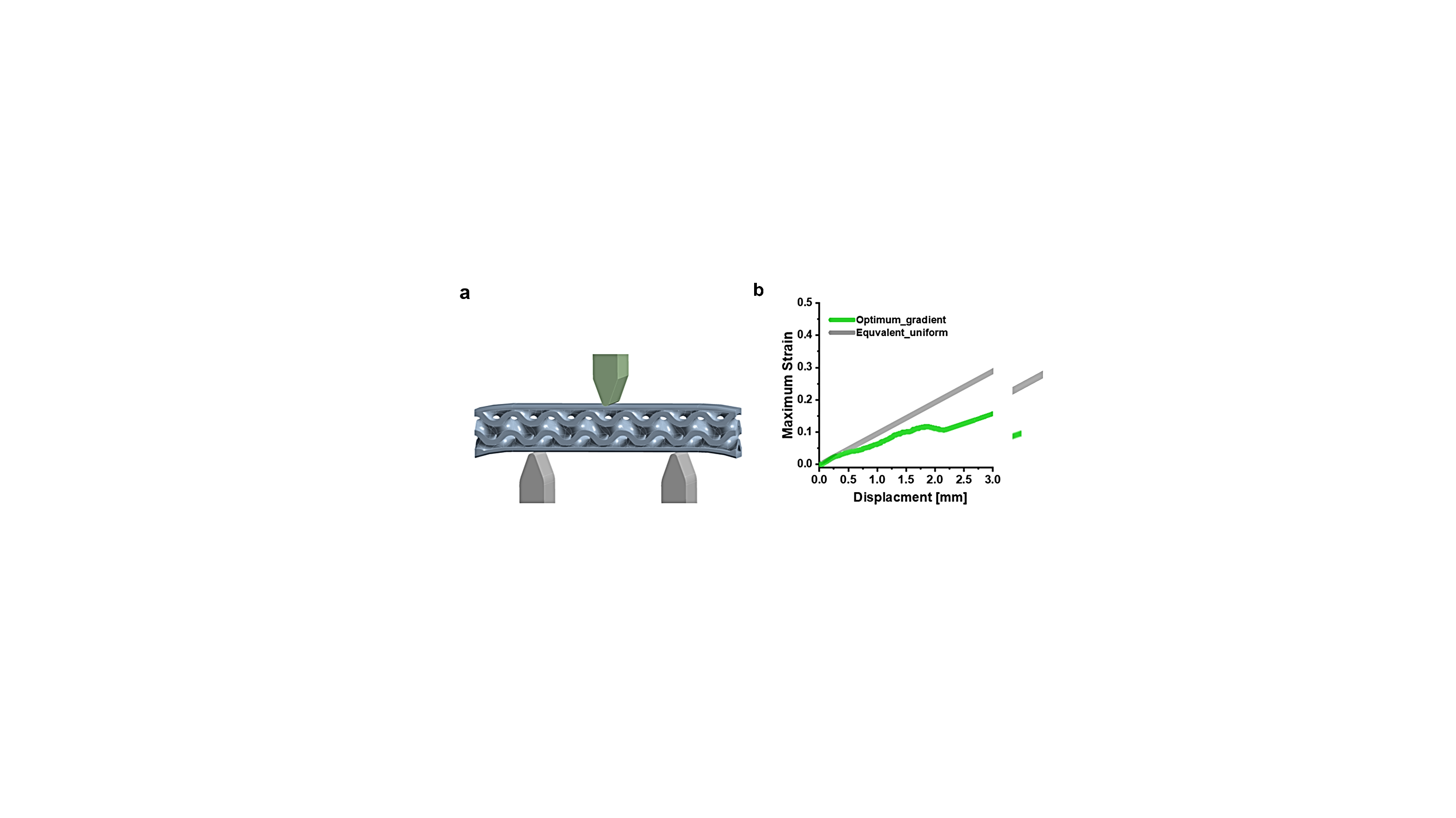


**Figure S34.** Three-point bending simulation setup for an automotive energy absorption beam. (a) Simulation setup and (b) displacement versus maximum strain plot for the uniform and gradient cases.


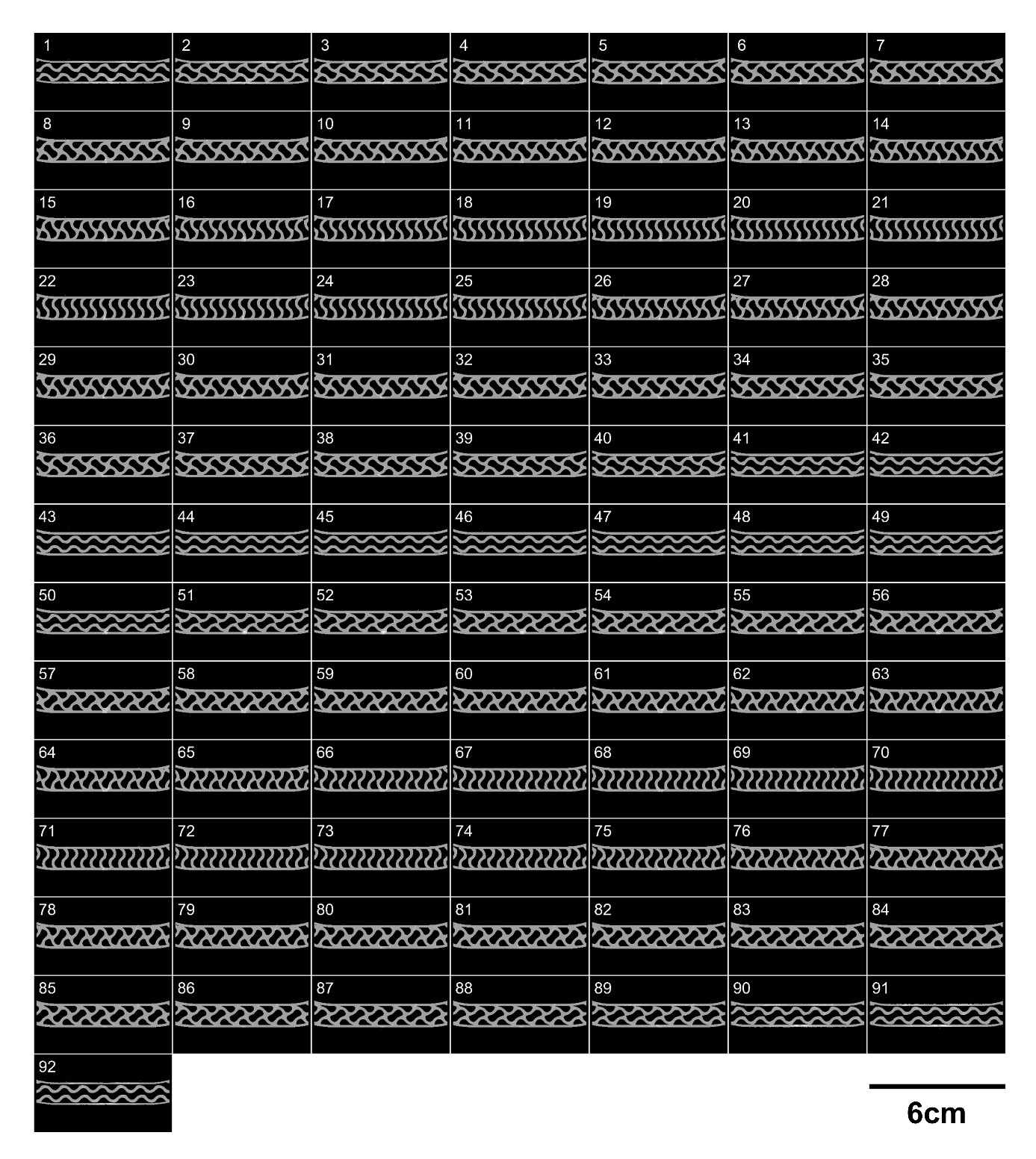


**Figure S35.** Sliced images generated for g-DLP 3D printing of voxel-based gradient structures for gyroid shell-based bumper beam. All images are 1920×1080 pixels in size.

**Figure S36** provides additional details of the drop test setup and results shown in Figure 6i. Specifically, (a) shows the experimental setup, (b) presents sequential images of the gradient structure during impacts from the 1st to the 14th cycle, and (c) shows the uniform structure from the 1st to the 9th cycle, highlighting the fracture progression.


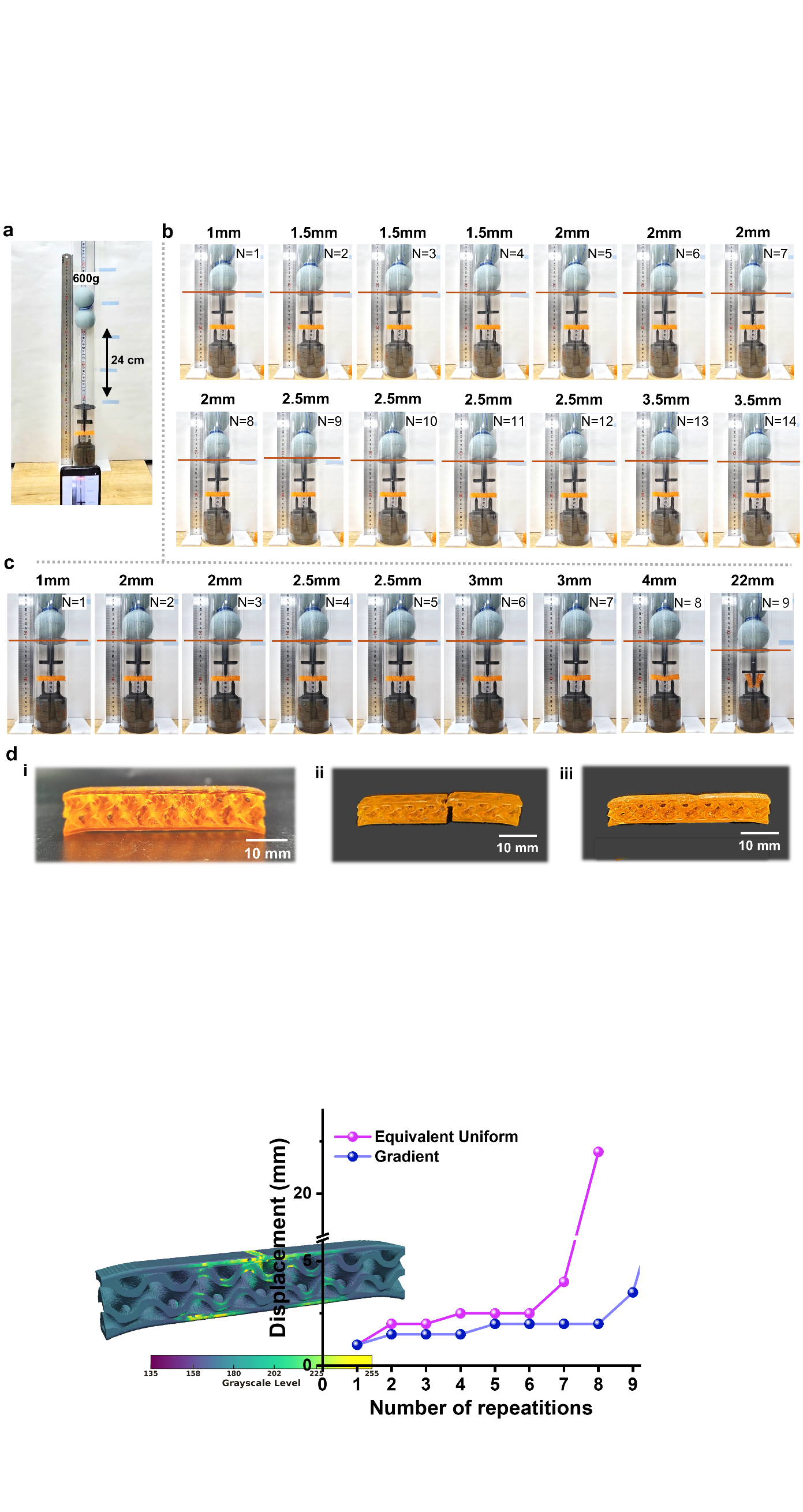


**Figure S36.** (a) Experimental setup for the repeated drop tests. (b) Sequential images showing the impact response of the gradient structure from the 1st to the 14th cycle. (c) Sequential photos of the uniform structure from the 1st to the 9th cycle. (d) 3D printed samples: (i) as-printed condition before testing; (ii) fractured uniform sample after testing; and (iii) intact gradient sample after testing.

**References**

[1] C. Hepburn, *Polyurethane Elastomers*, Dordrecht, Netherlands: Springer, 1991.
[2] G. Dong, Y. Chang, C. Li, L. Zhao, X. Tian, X. Liu, *J. Appl. Polym. Sci.* 2023, *140*, e54544.
[3] Materials Selection in Mechanical Design, 3rd ed., Elsevier Butterworth-Heinemann, 2005
[4] L. G. Ribeiro, M. A. Maia, E. Parente Jr., A. M. Cartaxo de Melo, *Compos. Struct.* 2020, *252*, 112677.
[5] H.-A. Bahr, H. Balke, T. Fett, I. Hofinger, G. Kirchhoff, D. Munz, A. Neubrand, A. S. Semenov, H.-J. Weiss, Y. Y. Yang, *Mater. Sci. Eng. A* 2003, *362*, 2–16.
[6] P. Gu, R. J. Asaro, *Int. J. Solids Struct.* 1997, *34*, 1–17.
[7] Y. Collette, P. Siarry, *Multi-objective Optimization: Principles and Case Studies*, Berlin, Germany: Springer, 2004.
[8] M. Balandat, B. Karrer, D. R. Jiang, S. Daulton, B. Letham, A. G. Wilson, E. Bakshy, *Adv. Neural Inf. Process. Syst.* 2020, *33*, 21524.
[9] C. B. Sullivan, A. A. Kaszynski, *J. Open Source Softw.* 2019, *4*, 1450.
